# Hybrid Assembly Provides Improved Resolution of Plasmids, Antimicrobial Resistance Genes, and Virulence Factors in *Escherichia coli* and *Klebsiella pneumoniae* Clinical Isolates

**DOI:** 10.3390/microorganisms9122560

**Published:** 2021-12-10

**Authors:** Abdolrahman Khezri, Ekaterina Avershina, Rafi Ahmad

**Affiliations:** 1 Department of Biotechnology, Inland Norway University of Applied Sciences, 2318 Hamar, Norway; Abdolrahman.khezri@inn.no (A.K.); ekaterina.avershina@inn.no (E.A.); 2 Faculty of Health Sciences, Institute of Clinical Medicine, UiT-The Arctic University of Norway, Hansine Hansens veg 18, 9019 Tromsø, Norway

**Keywords:** Oxford Nanopore, Illumina, short-read, long-read, hybrid assembly, antimicrobial resistance, virulence factors, clinical isolates, blood culture, plasmids

## Abstract

Emerging new sequencing technologies have provided researchers with a unique opportunity to study factors related to microbial pathogenicity, such as antimicrobial resistance (AMR) genes and virulence factors. However, the use of whole-genome sequence (WGS) data requires good knowledge of the bioinformatics involved, as well as the necessary techniques. In this study, a total of nine *Escherichia coli* and *Klebsiella pneumoniae* isolates from Norwegian clinical samples were sequenced using both MinION and Illumina platforms. Three out of nine samples were sequenced directly from blood culture, and one sample was sequenced from a mixed-blood culture. For genome assembly, several long-read, (Canu, Flye, Unicycler, and Miniasm), short-read (ABySS, Unicycler and SPAdes) and hybrid assemblers (Unicycler, hybridSPAdes, and MaSurCa) were tested. Assembled genomes from the best-performing assemblers (according to quality checks using QUAST and BUSCO) were subjected to downstream analyses. Flye and Unicycler assemblers performed best for the assembly of long and short reads, respectively. For hybrid assembly, Unicycler was the top-performing assembler and produced more circularized and complete genome assemblies. Hybrid assembled genomes performed substantially better in downstream analyses to predict putative plasmids, AMR genes and β-lactamase gene variants, compared to MinION and Illumina assemblies. Thus, hybrid assembly has the potential to reveal factors related to microbial pathogenicity in clinical and mixed samples.

## 1. Introduction

The pathogenicity of bacteria is often associated with antimicrobial resistance genes and/or virulence factors. Antimicrobial resistance (AMR) is the ability of microorganisms to defy antimicrobials, such as antibiotics. Globally, infections due to AMR bacteria are increasing and considered a threat to modern health care [1,2]. During the last two decades, scientific communities have seen a growing trend towards using next-generation sequencing (NGS) technology such as Illumina sequencing to identify AMR genes and virulence factors. Although NGS provides high depth coverage data, the output reads from NGS platforms such as Illumina are only about a few hundred base pairs long. Therefore, constructing a genome assembly based on the short-reads often results in an incomplete and fragmented assembly, which makes downstream analyses challenging [3]. 

New sequencing technologies known as third-generation sequencing technologies have been developed to overcome the short-read sequencing limitations. Pacific Biosciences (PacBio) is one of the most successful platforms for generating long reads [4]. One of the latest examples of devices that benefit from the new sequencing technology is the MinION sequencer from Oxford Nanopore Technologies (ONT). It can produce reads up to 2.3 million bases in length [5], which is sufficient to satisfy the repetitive elements flanked to the AMR genes [6]. Despite the advantages of long reads, they suffer from a high sequencing error rate [7], mainly due to older flowcells, kits and base calling algorithms. It has been shown that such reads remained error prone, even after error correction and polishing [8]. These properties restrict the usage of long reads to the study of small plasmids, which might carry AMR genes [9]. However, recent developments in flowcells and MinION sequencing chemistry, as well as more accurate neural network models used for MinION base calling, have greatly reduced the error rate [10]. 

Considering the benefits and drawbacks of both short and long reads, several attempts have been made to apply a hybrid assembly approach, which uses both type of reads [3,9,11,12,13]. For this purpose, different assemblers, such as Unicycler [3], hybridSPAdes [14], and MaSurCa [15], have been developed. All these hybrid assemblers benefit from the greater depth of short reads and increased length of long reads. Hybrid assembly offers several advantages over *de novo* assembly solely using short or long reads. For instance, hybrid assembly makes the downstream analyses, mapping, and annotation of genomic features more accurate [16]. Furthermore, it has been shown that hybrid assembly provides a better resolution for studying tandem repeats as well as gene variants [17], and it is the ideal approach for predicting the plasmids and AMR genes [13]. 

In recent years, different long, short and hybrid assemblers have been developed and tested, mainly using environmental samples [18]. The performance of different assemblers and the success of different assembly approaches for clinical isolates, especially where multiple bacteria are present, is unclear. Therefore, this study specifically focused on clinical isolates as well as blood samples spiked with bacteria species to mimic realistic clinical scenarios. Here, we aimed to compare the different tools available for constructing the short, long and hybrid assemblies and identify the top-performing assemblers for each approach. Secondly, we intended to identify plasmids, potential AMR genes, and virulence factors in assemblies produced by the top-performing assemblers for each approach.

## 2. Materials and Methods

### 2.1. Sample Collection and Characterization

In the present research, nine isolates consisting of four *E. coli* (1–4) and five *K. pneumoniae* (1–5) isolates, isolated from blood specimens of Norwegian patients, were used. The bacteria were grown overnight on agar plates, as described previously [19]. An overview of the samples and culture system is presented in Supplementary Table S1. 

### 2.2. Spiking the Blood Samples and Incubation of Blood Cultures

Spiking and culturing of the blood samples was performed using two *K. pneumoniae* and one *E. coli* isolates at Oslo University Hospital, as described previously [20]. In brief, human blood was obtained from healthy anonymous donors via the blood bank at Oslo University Hospital and were transformed to four BD BACTEC 40 mL flasks (Becton, Franklin Lakes, NJ, USA). Then, blood samples in the flasks were spiked with isolate *E. coli* 4 (A2-39) and isolates *K. pneumoniae* 4 (A2-23), *K. pneumoniae* 5 (A2-37) and both *E. coli* 4 and *K. pneumoniae* 5 (mixed culture sample). The flasks were incubated in a BD BACTEC FX blood culture instrument until the culture was flagged positive.

### 2.3. Library Preparation and Whole-Genome Sequencing

The bacterial DNA from four blood cultures and six fresh grown isolates (three *K. pneumoniae* and three *E. coli*) was isolated and the libraries for Nanopore sequencing were constructed according to previously published protocols [19,20]. In brief, purified DNA was barcoded using the Rapid Barcoding Sequencing kit SQK-RBK004 (Oxford Nanopore, Oxford, UK) and further purified using an Agencourt AMPure XP system (Beckman Coulter, Brea, CA, USA). Sequencing, data collection and base calling (high accuracy mode) were performed using MinION flow cells (R9.4.1 FLO-MIN106, Oxford Nanopore), MinKNOW software v3.6.5 and Guppy basecaller v3 (ONT), respectively. Human data were discarded, and reads were categorized based on the read quality score as pass (≥5) or fail (<5) by the basecaller. DNA libraries for Illumina sequencing were prepared using Illumina Nextera XT DNA sample preparation kit (Illumina, San Diego, CA, USA). Illumina libraries were sequenced in pair-end mode (2 × 300 bp) using the Illumina MiSeq platform.

### 2.4. Bioinformatic Analyses of Bacterial Genomics

#### 2.4.1. Quality Control and Trimming of Illumina and Nanopore Reads

Illumina reads were quality checked using FastQC (v0.11.8 for Linux) [21], adapters were removed, and low-quality reads (Phred < 25) were filtered out using Trimmomatic with default parameters [22], integrated into OmicsBox (v1.4.11 for Linux) [23]. For MinION reads, adapter and barcode trimming were performed using Porechop (v0.2.4 for Linux) with default settings [11]. Long and high-quality reads were collected using Filtlong (v0.2.0 for Linux) with default parameters [24]. Before downstream analyses, basic quality and statistics of long reads were checked using NanoPlot [25].

#### 2.4.2. Bacterial Whole-Genome Assembly and Visualization

In this study, genome assemblies from Illumina short-reads (hereafter referred to as Illum_ASM_) were created using SPAdes (v3.11.1) [26], Unicycler (v0.4.9) [3] and ABySS (v2.3.0) [27] assemblers. For the assembly of MinION long-reads (hereafter referred to as MinION_ASM_), different assemblers, including Unicycler, Flye (v2.8.2) [28], Canu (v1.7.1) [29] and Miniasm (v0.3.0) [30], were tested. Later, Illumina short-reads and MinION long-reads were combined to construct hybrid assembly (hereafter referred to as Hyb_ASM_) using Unicycler, hybridSPAdes [14] and MaSurCa [15] assemblers.

General assembly statistics and quality of the assembled genomes were calculated using QUAST (v4.6.0 for Linux) [31] and BUSCO, which evaluate assemblies for highly conserved genes and generate a completeness score for the genome [32]. Furthermore, assembly visualization was performed using Bandage (v0.8.1 for Windows) [33]. For each of the short, long and hybrid reads, only one assembly (based on QUAST and BUSCO results, as well as the circularity of assemblies) was considered for downstream analyses (in total, three assembles per isolate).

In addition to the isolates, we have considered the *E. coli* NCTC strain 13441 as a reference genome. This strain was cultured in blood and sequenced directly from blood using MinION, as described in Section 2.2 and Section 2.3. In order to create Illumina reads for *E. coli* strain NCTC 13441, assembly file for this strain, was downloaded from NCBI assembly database (https://www.ncbi.nlm.nih.gov/assembly/GCF_900119685.1, access date: 25 September 2021) and short Illumina MiSeq reads were re-generated *in silico* from assembly file using InSilicoSeq sequencing simulator [34]. Reference genome assemblies were created using the top-performing assemblers, which described and identified for other isolates. Reference genome assemblies were considered for all downstream analyses and the results were considered as ground truths for *E. coli* isolates. The basic information for sequence, assembly and downstream analyses for reference samples is presented in Supplementary Table S2.

#### 2.4.3. Bacterial Whole-Genome Annotation

Genome assemblies for each isolate were annotated using Prokka (v1.14.5 for Linux) [35], and information regarding different genomic features, such as coding sequence (CDS), tRNA, rRNA, tmRNA, and repeat regions, was extracted

#### 2.4.4. Bacterial Plasmid Identification

Generated assembly files for each isolate were used to identify plasmids. For this purpose, the PlasmidFinder online tool (software version: 2.0.1, database version: 2020-07-13) [36], with minimum identity 95% and coverage 60%, was utilized. Plasmid hits were further visually confirmed for circularity using assembly graphs constructed in Bandage.

#### 2.4.5. Detection of Antimicrobial Resistance Genes

In this study, AMR genes associated with mobile elements on chromosome/plasmids were identified using ResFinder online tool (v4.1, software version: 2020-10-21, database version: 2020-12-01) [37]. Only hits showing ≥95% identity and length coverage were considered as true AMR genes. To identify AMR genes associated with a chromosomal point mutation, the PointFinder online tool (software version: 2020-10-21, database version: 2019-07-02) [38], with the same search criteria as ResFinder, was used.

#### 2.4.6. Bacterial Virulence Factor Identification

To identify virulence factors (VFs) hosted either by plasmids or chromosomes, the nucleotide virulence factor database (VFDB) was downloaded (database version: 2020-11-18) [39]. Then, the assembled genomes were BLAST-searched against the downloaded VFDB. Only hits with identity and alignment coverage ≥95% and e-values of 0 were considered as virulence factors.

## 3. Results

### 3.1. Basic Statistics of Short and Long Reads

Basic read information for both MinION and Illumina reads is presented in Table 1. Isolates *E. coli* 3 and *K. pneumoniae* 1 showed low read coverage, and isolate *K. pneumoniae* 2 showed remarkably high read coverage for their respective MinION long reads. These isolates had comparable read coverage for their Illumina short reads. Overall, Illumina reads clearly had higher coverage compared to MinION reads for *E. coli* isolates, whereas for *K. pneumoniae*, an opposite trend was observed.

### 3.2. Unicycler Performed Better Than SPAdes and ABySS for the Assembly of Short-Reads

In this study, short reads were assembled using Unicycler, SPAdes and ABySS assemblers. According to QUAST and BUSCO results, Unicycler and SPAdes performed similarly and better than ABySS (Supplementary Table S3 and Supplementary Figure S5). The coverage fraction of reference genome and N50 average value for *E. coli* isolates, indicated a better performance of SPAdes over Unicycler (85.5% genome fraction vs. 82.5% and 237,038 bp vs. 225,244 bp N50). However, for *K. pneumoniae*, an opposite trend was observed, and the average N50 value was higher for Unicycler as compared to SPAdes (292,361 vs. 259,498). Although the core algorithm in Unicycler for the assembly of short reads is still SPAdes, the Unicycler assembler produced better assemblies compared to SPAdes alone. For instance, the assemblies from Unicycler had fewer contigs (on average, 138 for *E. coli* and 78 for *K. pneumoniae* in Unicycler vs. 243 for *E. coli* and 585 for *K. pneumoniae* in SPAdes). Furthermore, more circularized chromosomes and/or plasmids were observed in assemblies from Unicycler and the number of dead ends (number of occurrences where an end of a node does not connect to any other nodes) was also fewer (on average, 4 for *E. coli* and 9 for *K. pneumoniae* in Unicycler vs. 440 for *E. coli* and 1654 for *K. pneumoniae* in SPAdes) (Supplementary Figure S1). A similar better performance of Unicycler over SPAdes was documented for mixed culture sample (Supplementary Table S3). Therefore, all downstream analyses for short reads were performed using assemblies from Unicycler.

### 3.3. Flye as a Top-Performing Assembler for MinION Long-Reads

We compared different assemblers to assemble the MinION long-reads. Based on the QUAST assembly statistics, Flye and Canu clearly outperformed the other assemblers. Although the *E. coli*-assembled genomes using Flye covered a smaller portion of the reference genome compared to assemblies made by Canu (68.2% for Canu and 55.5% for Flye), Flye statistics were higher compared to Canu for other parameters. For instance, the average N50 value for *K. pneumoniae* isolates was 1,996,100 bp (Flye) and 1,789,477 bp (Canu). For *E. coli* isolates, the average N50 value was 343,234 bp (Flye) and 435,539 bp (Canu) (Supplementary Table S3). Furthermore, after the visualization of assembly files, more circularized chromosomes and/or plasmids and fewer dead-ends (Supplementary Figure S2) were observed for Flye. The average dead-end number was 77 for *E. coli* and 44 for *K. pneumoniae* isolates for Flye vs. 287 for *E. coli* and 135 for *K. pneumoniae* isolates using Canu (Supplementary Figure S2). The BUSCO analyses (Supplementary Figure S5), showed that assemblies constructed using Flye had better average BUSCO results compared to Canu (27.7% complete, 22.6% fragmented and 49.7% missing for Flye vs. 22.7% complete, 25% fragmented and 53.3% missing for Canu). Therefore, all downstream analyses for long-read sequences were performed using assemblies from Flye.

### 3.4. Unicycler Produced Superior Hybrid Assemblies over hybridSPAdes and MaSurCa

To make hybrid assemblies, we have tested three different tools. According to QUAST and BUSCO results, Unicycler and hybridSPAdes showed comparable and better performance than MaSurCa (Supplementary Table S3 and Supplementary Figure S5). For the mixed sample, MaSurCa performed excellently and displayed a higher genome fraction and N50 as well as a lower number of contigs. For *E. coli* isolates, the fraction of the reference genome which was covered by *E. coli* isolates assembly, was marginally higher for hybridSPAdes compared to Unicycler (83.2% vs. 82.9%). The average N50 value for *E. coli* isolates was 1,474,667 bp for hybridSPAdes, followed by Unicycler (1,005,273 bp). For *K. pneumoniae* isolates, the average N50 value was 3,880,247 bp for Unicycler, followed by hybridSPAdes (3,737,967 bp). Moreover, Unicycler produced fewer fragmented assemblies as compared to hybridSPAdes. For instance, assembly graphs indicated more circularized chromosomes and plasmids for Unicycler assemblies compared with hybridSPAdes (Supplementary Figure S3). Furthermore, assemblies produced using Unicycler had fewer dead ends (four and one dead ends for *E. coli* and *K. pneumoniae* isolates using Unicycler vs. 6072 and 5143 dead ends using hybridSPAdes, respectively). Therefore, all downstream analyses for hybrid assemblies were performed using hybrid genomes assembled using Unicycler.

### 3.5. Assembly Comparison between the Top-Performing Long, Short and Hybrid Read Assemblers

An overview of assembly statistics for the best Illum_ASM_ (using Unicycler), MinION_ASM_ (using Flye), and Hyb_ASM_ (using Unicycler) are presented in Table 2. Results for individual isolates can be found in Supplementary Table S4. Overall, Hyb_ASM_ provided more complete and circular genomes. For both mono- and mixed culture isolates, Illum_ASM_ was more fragmented (higher number of contigs), followed by MinION_ASM_ and Hyb_ASM_. Furthermore, the N50 value was higher in Hyb_ASM_ followed by MinION_ASM_ and Illum_ASM_.

**Figure 1:**
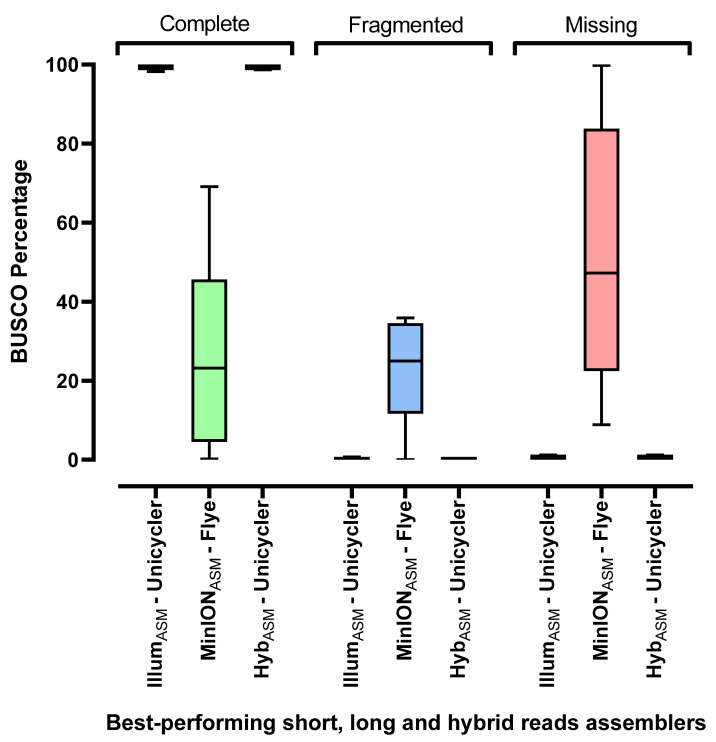
Box plots for BUSCO results of the best-performing assemblers. Illum_ASM_ was produced using Unicycler, MinION_ASM_ using Flye and Hyb_ASM_ was created using Unicycler. Each box extends from Min to Max values in each group and the middle black line in each box indicates the mean value. The BUSCO percentage for mixed samples is not included in the graph.

The BUSCO results (Figure 1) showed a similar performance of Hyb_ASM_ and Illum_ASM_ (on average, 0.6% BUSCO missing rate for both Hyb_ASM_ and Illum_ASM_ and 99.3% and 99.2% BUSCO complete for Hyb_ASM_ and Illum_ASM_, respectively). Interestingly, MinION_ASM_ performed worst in comparison to both Hyb_ASM_ and Illum_ASM_. For instance, 22.6% of candidate genes in BUSCO were fragmented and only 27.7% were complete, whereas 49.7% of BUSCO genes were reported as missing in MinION_ASM_.

**Figure 2:**
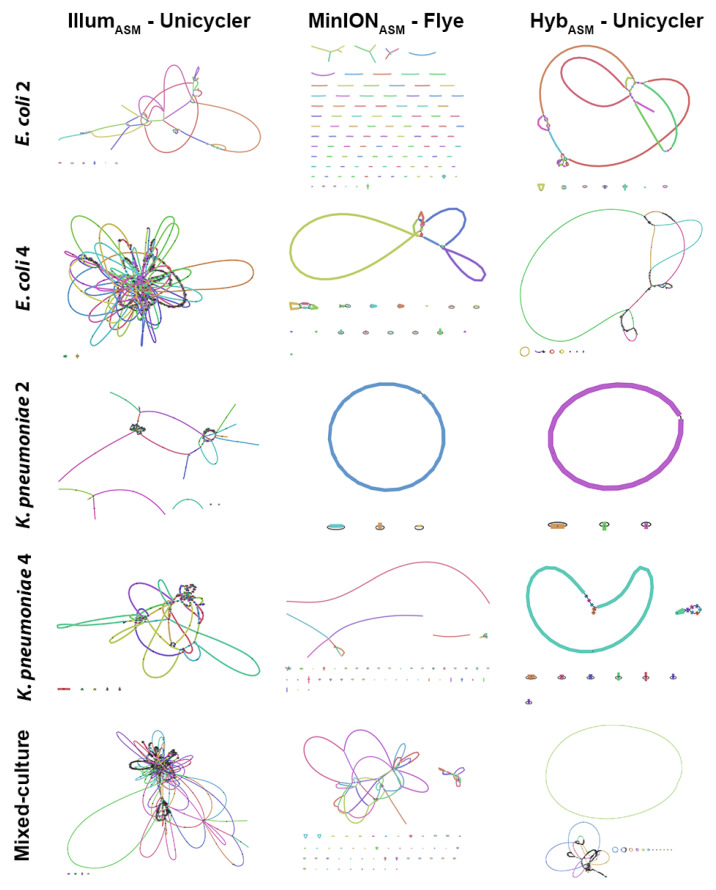
Representative assembly graphs for some of the isolates including *E. coli* 2 and 4, *K. pneumoniae* 2 and 4 as well as a mixed sample from the co-culturing of *E. coli* 4 and *K. pneumoniae* 5 isolates. The GFA files produced by the top-performing assemblers (Unicycler for Illumina short reads, Flye for MinION long reads and Unicycler for hybrid reads) were used to construct the assembly graphs using Bandage. Illumina assemblies were fragmented, and putative plasmids were limited. MinION produced much larger contigs and more putative plasmids. However, proper circular chromosomes were not observed for the majority of isolates using either Illum_ASM_ or MinION_ASM_. However, hybrid assemblies provided us with clear and close chromosome/putative plasmids.

Using MinION_ASM_ alone, we were able to close the chromosome for *K. pneumoniae* 2, similar to Hyb_ASM_ for the same isolate. In contrast, the Illum_ASM_ was fragmented for the same isolate (Figure 2). Overall, using Hyb_ASM_, we were able to close the chromosome structure for three *K. pneumoniae* isolates (2, 3, and 4), whereas no circularized chromosome was obtained for *E. coli* isolates (Supplementary Figure S4). For the isolate from the mixed sample, two clear chromosomes were reconstructed using Hyb_ASM_, including one circular chromosome. The circular contig sequence was BLAST-searched using PATRIC [40], and the results showed 94% identity to the complete *K. pneumoniae* subsp. pneumoniae genome.

### 3.6. Whole-Genome Annotation of the Short, Long and Hybrid Assemblies

**Figure 3:**
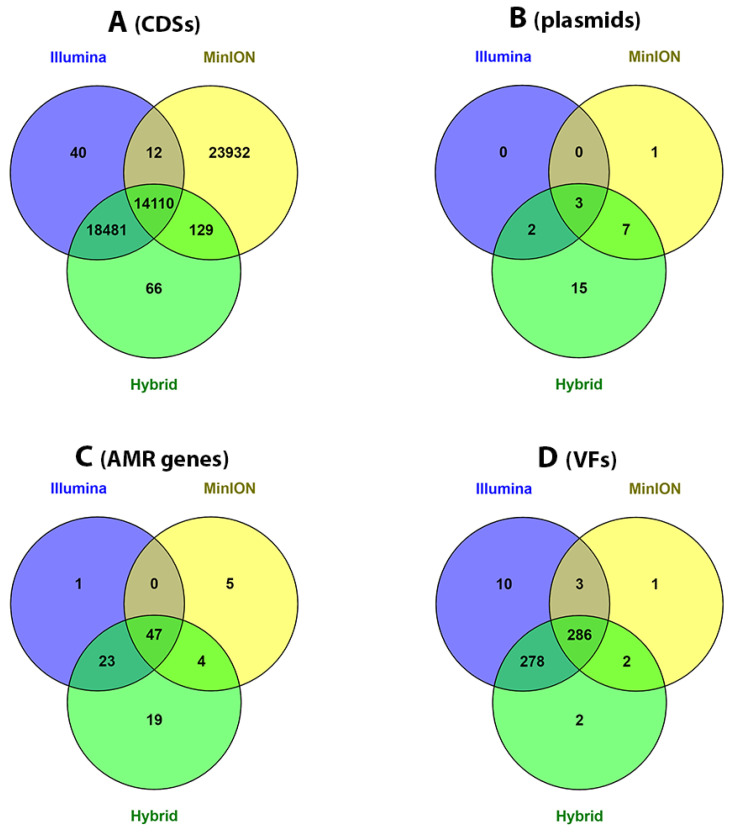
An overview of downstream analysis results for different assemblies created using the top-performing assemblers. Venn diagrams prepared using the Venny online platform to plot differences in the number of annotations obtained, in which data for four *E. coli* and five *K. pneumoniae* isolates were merged. Numbers in the overlap area indicate the mutual hit names (hits identified in the exact same isolates). (**A**) The number of annotated CDSs (putative and hypothetical proteins not plotted). (**B**) The number of identified and confirmed plasmid contigs using PlasmidFinder and Bandage visualization tools, respectively. (**C**) The number of AMR genes, including both acquired and point mutations. (**D**) The number of identified VFs.

Results of the genome annotation are presented in Table 3 and Supplementary Table S5. The MinION data were not sufficient to capture all tRNA in the isolates as compared to Illum_ASM_ and Hyb_ASM_. The overlaps between annotated CDSs from various assemblies for all the isolates (except the isolate from mixed culture) is presented in Figure 3A. Both Illum_ASM_ and Hyb_ASM_ exhibited comparable results, whereas MinION_ASM_ showed divergent results compared to Illum_ASM_ and Hyb_ASM_. For instance, using MinION_ASM_, a total number of 23,932 annotated CDSs were exclusively identified in isolates and, on average, MinION data had up to two times more annotated CDS. However, when we searched for which CDSs contributed to such high difference, the majority of these ‘extra’ CDSs belonged to duplicates of genes detected in Hyb_ASM_ (Supplementary Table S6). Annotations of Illum_ASM_, MinION_ASM_ and Hyb_ASM_ for *E. coli* isolates, on average, resulted in identifying of 3632, 4212 and 3684 CDSs, respectively (hypothetical and putative proteins were not considered). This corresponded to 0.55% fewer CDSs in Illum_ASM_ compared to the Illumina assembly of the reference genome (*E. coli* NCTC 13441) (Supplementary Table S2). Furthermore, on average, MinION_ASM_ and Hyb_ASM_ predicted 3.8% and 0.21% more CDSs, respectively, as compared with corresponding assemblies of the reference genome. Results for annotated rRNA and tRNA indicated that Hyb_ASM_ showed closer association with corresponding data from the reference genome than Illum_ASM_ and MinION_ASM_.

### 3.7. Plasmid Identification in Short, Long and Hybrid Assemblies

As can be seen from Figure 3B, more plasmids (confirmed using Bandage) were identified in Hyb_ASM_ (11 plasmids for *E. coli* isolates, 16 plasmids for *K. pneumoniae* isolates), followed by MinION_ASM_ (3 plasmids for *E. coli* isolates, 8 plasmids for *K. pneumoniae* isolates) and Illum_ASM_ (3 plasmids for *E. coli* isolates, 2 plasmids for *K. pneumoniae* isolates). The majority of detected plasmids hosted *IncF* replicons in both *E. coli* and *K. pneumoniae* isolates. Only three plasmids (Col156, Col8282 and ColpVC), ranging from 1981 to 5146 bp in length, were detected in all three assembles. All three types of assembled genomes for the reference isolate (*E. coli* NCTC 13441), indicated that the reference genome (Supplementary Table S2) could have up to two plasmids (IncFIA and IncFII). The Illum_ASM_, MinION_ASM_ and Hyb_ASM_ results showed that *E. coli* isolates could have up to three, two and four putative plasmids, respectively. The complete list of plasmids and replicons is presented in Supplementary Table S7.

### 3.8. Identification of Acquired Antimicrobial Resistance Genes and Mutations

As shown in Figure 3C, using Hyb_ASM_, we were able to identify more antimicrobial resistance genes (16 genes for *E. coli* isolates, 77 genes for *K. pneumoniae* isolates) than Illum_ASM_ (16 genes for *E. coli* isolates, 55 genes for *K. pneumoniae* isolates). MinION_ASM_ demonstrated the worst performance in predicting the AMR genes (15 genes for *E. coli* isolates and 43 genes for *K. pneumoniae* isolates). Overall, 47% of identified AMR genes were found to be common between all types of assemblies.

Furthermore, we have identified chromosomal mutations conferring resistance to antibiotics for all the different assemblies. For all isolates from both mono- and mixed cultures, Hyb_ASM_ and Illum_ASM_ results were entirely identical (genes such as gyrA, parC, parE, acrR, ompK37 and ramR were identified at the identical isolates using both Illum_ASM_ and Hyb_ASM_). Results from MinION_ASM_ showed partial overlap (only ompK37 and ramR genes) with Hyb_ASM_ and/or Illum_ASM_ (Supplementary Table S8).

We were particularly interested in identifying different variants of β-lactamase genes in different assemblies. Using Hyb_ASM_ and not Illum_ASM_ or MinION_ASM_, we were able to identify a variety of β-lactamase genes mostly belonging to different variants of blaTEM (1C, 29, 55, 57, 122, 135, 141 and 209) and blaSHV (28, 31, 40, 56, 76, 79, 85, 89, 106, 164 and 172) genes. AMR genes such as blaTEM-1B, blaSHV-187, blaCTX-M (14, 15) and blaOXA-9 were the only β-lactamase genes identified in the same isolates using all types of assemblies (Supplementary Table S8).

Data from *E. coli* reference genome (Supplementary Table S2) showed that the reference genome could have up to 14 AMR genes (in Illumina and hybrid assemblies) and 9 AMR genes in the MinION assembly. Here, and on average, we identified four AMR genes per isolate (in each of the assemblies for *E. coli* isolates).

### 3.9. Identification of Virulence Factors in Short, Long and Hybrid Assemblies

Using the VFDB core database, we identified bacterial virulence factors. In all three different assemblies, the number of identified virulence factors was higher in *E. coli* than in *K. pneumoniae*. As shown in (Figure 3D), the majority of identified VFs were mutual between Hyb_ASM_ and Illum_ASM_; therefore, Illum_ASM_ and Hyb_ASM_ showed almost similar performance. Similar results were observed for the reference sample (Supplementary Table S2). MinION_ASM_ covered fewer VFs (136 VFs for *E. coli*, 156 VFs for *K. pneumoniae*). However, all the hits (291 VFs), except just one VF in MinION_ASM_, were detected using either hybrid or Illumina assemblies. Results for each individual isolate are presented in Supplementary Table S9.

The reference genome for *E. coli* showed 46 (based on long read assembly) and 85 (based on short read and hybrid assemblies) VFs (Supplementary Table S2). In comparison with the reference genome, and on average, we identified 76, 34 and 74 VFs for *E. coli* isolates using Illum_ASM_, MinION_ASM_ and Hyb_ASM_, respectively_._

### 3.10. Hyb_ASM_ Enables the Complete Recovery of Plasmid Replicons, AMR Genes, and Virulence Factors from the Mixed Culture Sample

According to MinION data, Isolate *E. coli* 4 and *K. pneumoniae* 5 possessed p0111 and IncFII replicons, respectively. In the mixed culture sample, the MinION_ASM_ failed to recover the IncFII plasmid replicon from *K. pneumoniae* 5. In contrast, hybrid data revealed replicons such as IncHI2, IncHI2A, and p0111 in *E. coli* 4, as well as replicons such as IncFIA(HI1), IncFIB(K), IncFII, IncFII(pKP91) in *K. pneumoniae* 5. Interestingly, Hyb_ASM_ recovered all mentioned plasmid replicons in mixed samples too. Regarding recovering the AMR genes, although Hyb_ASM_ was able to recover all the AMRs identified in both *E. coli* 4 and *K. pneumoniae* 5 for mixed samples, Illum_ASM_ and MinION_ASM_ each missed one gene (sul1 and 16S_rrsC in *E. coli* 4 for Illum_ASM_ and MinION_ASM_, respectively). Similar to recovering the AMR genes, Hyb_ASM_ recovered all the VFs (plus two more VFs) in the mixed culture sample. Illum_ASM_ also was able to recover complete VFs in the mixed sample, which were identified individually in *E. coli* 4 and *K. pneumoniae* 5. Although MinION_ASM_ identified 19 unique VFs in the mixed culture sample, it missed 10 VFs in *E. coli* 4 (data corresponding to annotation, plasmid replicons, AMR and VF for *E. coli* 4, *K. pneumoniae* 5 and the mixed sample are presented in Supplementary Table S10).

## 4. Discussion

In the current study, we tested different short, long and hybrid read assemblers. The assembled genomes from the top-performing assemblers in each approach were subjected to downstream analyses.

For short read assembly, ABySS, Unicycler and SPAdes were tested; based on both QUAST and BUSCO results, ABySS performed worse than Unicycler and SPAdes. In line with our results, a better performance of SPAdes over ABySS previously has been documented for the *de novo* assembly of small RNA-Seq samples taken from plant species [41]. This observation might be explained by the fact that SPAdes takes advantages of various Kmer sizes simultaneously, whereas in ABySS, one must specify the Kmer cut-off size. In this study, assembly statistics and graphs indicated a slightly better performance of Unicycler over SPAdes to assemble the short reads. Although SPAdes is the main algorithm implemented in Unicycler, the better performance of Unicycler might be explained by the implementation of additional steps such as strict filtering steps, repeat resolution algorithm and polishing [3].

In this study, we observed a comparable result between Flye and Canu to assemble the long reads. However, a higher degree of genome circularization was observed in assemblies produced by the Flye assembler. Similar conclusions between Flye and Canu assemblers were made in previous research, where the authors tested different assemblers for prokaryote whole-genome sequencing [42]. Following the present results, previous studies have demonstrated that both Flye and Canu assemblers could be considered as the first choice to assemble not only prokaryote genomes, but also plant and crop genomes based on long reads [43,44]. We observed a low BUSCO score using long reads. A similar low BUSCO score for assemblies based on MinION reads has previously been observed [45]. This might be explained by the low coverage of ONT reads. Overall, according to the BUSCO results, the Flye assembler performed best. This might be explained by the five polishing steps performed using integrated Pilon software with Flye; prior studies have reported that polishing the MinION assembly increases the BUSCO completeness score [45,46]. Although the Canu assembler takes advantage of polishing using both Racon and Pilon (two rounds each), the BUSCO completeness score for Canu was considerably lower than Flye. At the same time, QUAST statistics were similar for both Canu and Flye. Therefore, to draw the conclusion regarding choosing the appropriate assembler for long reads, it may be necessary to evaluate the assemblies using both QUAST and BUSCO. Here, using both tools, we observed superior long-reads assembly for Flye as compared to Canu. Although the BUSCO score for Miniasm indicated an acceptable performance, the QUAST statistics demonstrated weak performance for this assembler. However, it must be kept in mind that Miniasm still is in the development phase, and it does not perform any polishing or read correction processes for MinION data [30].

Furthermore, we have tested three different tools for making a hybrid assembly. Both QUAST and BUSCO documented a similar performance in Unicycler and hybridSPAdes and a less efficient performance in MaSurCa. In accordance with the current study, previous research has revealed that both hybridSPAdes and Unicycler produce more accurate hybrid assemblies compared with MaSurCa [47]. In this study, Unicycler produced less fragmented Hyb_ASM_ as compared with hybridSPAdes. Similar observations have previously been reported for clinical samples [47]. Differences between Unicycler and hybridSPAdes might be partially explained by different integrated polishers (i.e., Unicycler uses Pilon and SPAdes uses Racon for polishing) and the step where polishing is implemented. The average N50 values for *E. coli* Hyb_ASM_ using both Unicycler and hybridSPAdes were remarkably lower compared to the average *K. pneumoniae* N50 value. This might be explained by the low coverage of ONT data for *E. coli* (22.4× compared to *K. pneumoniae* (52.2×) isolates. Surprisingly, in our study, the MaSurCa assembler provided remarkably lower quality hybrid assembly (10 times lower N50 values) for isolates *E. coli* 3 and *K. pneumoniae* 1 compared to both Unicycler and hybridSPAdes. Our results documented a low MinION coverage for mentioned isolates. Therefore, the current finding suggest that low-quality long reads could greatly affect the hybrid assembly produced by MaSurCa, and both Unicycler and hybridSPAdes can tolerate more low-quality long reads. It is worth mentioning that the application of MaSurCa for bacterial hybrid genome assembly is limited thus far; therefore, applications of the MaSurCa assembler for clinical sampling deserve further investigation. 

In this study, we observed a considerable size variation in assembled genomes following the use of long reads from MinION. For instance, isolates *E. coli* 3 and *K. pneumoniae* 1 had a remarkably smaller genome size than Hyb_ASM_ or Illum_ASM_ (Supplementary Table S4). Inaccuracy in genome size using Nanopore technology has previously been reported for a conjugated test plasmid [13], and might be explained by the technology’s greater sequencing error [7,48]. In addition, inaccuracy in genome size can be explained by lower MinION coverage for the mentioned isolates. Lower MinION coverage might be related to a lower quantity and quality of isolated DNA. Due to the complexity of the samples, the DNA extraction could have compromised the recovery of long DNA molecules, thus affecting the N50 read length. Another reason which might explain the lower coverage for some of the samples is that the data for corresponding samples were generated during a rapid barcoding run, with six samples per run. According to rapid barcoding protocols, isolated DNA will not undergo PCR amplification during MinION library preparation. Notably, the *E. coli* 3 and *K. pneumoniae* 1 isolates showed remarkable examples where even a minimal quantity of long reads effectively contributed to improved Hyb_ASM_ results. The minimal quantity of long reads further reflected in QUAST results: *E. coli* 3 and *K. pneumoniae* 1 long reads only contributed as much as 46% and 3% in Hyb_ASM_ of corresponding isolates. However, and despite the minimal quantity of long reads, MinION data provided 74 and 248 Kb improvements in N50 (Supplementary Table S4) in the Hyb_ASM_ of mentioned isolates, respectively. These results provide proof for previous hypotheses suggesting that combining even a few long reads with short reads could be the most cost-effective way to map a complete bacterial genome [3].

Regarding the prediction of plasmids, more putative plasmids were detected using MinION_ASM_ as compared with Illum_ASM_. The poor performance of Illum_ASM_ to predict the plasmids is likely to be related to a higher level of fragmentation, which makes the reconstruction of plasmids difficult. The better performance of Hyb_ASM_ to resolve the putative plasmids in the current study agrees with a previous study, where small plasmids were absent from long-read assemblies but not from Hyb_ASM_ [49]. Furthermore, the superiority of hybrid assemblies (assembled using Unicycler) in the plasmid detection of clinical pathogens has previously been reported [50,51]. The numbers of both AMR genes and mutations, predicted here using Hyb_ASM_ for *E. coli* isolates (four AMRs/isolate), were lower than previously reported results for Hyb_ASM_ of clinical *E. coli* isolates in Canada (eight AMRs/isolate) [52] and less than the *E. coli* reference genome. This might be due to Norway’s lower antibiotic resistance occurrence; it is a country with one of the lowest drug resistance indexes [53].

Furthermore, our results showed that nanopore sequencing is not suitable for studying gene variants and/or predicting chromosomal mutations. For instance, using MinION_ASM_, we were not able to predict the AMR gene variants for β-lactamase genes (blaTEM and blaSHV variants), which only differ by one or a few base pairs. This is in line with our previous findings [20]. Although Illum_ASM_ performed marginally better in predicting these gene variants, Hyb_ASM_ performed best. MinION_ASM_ results for predicting chromosomal mutations also indicated poor performance, whereas Illum_ASM_ and Hyb_ASM_ yielded similar results. It seems possible that these results are due to the low sensitivity and high error rate of nanopore sequencing technology [7,48].

VF predictions showed that both Illum_ASM_ and Hyb_ASM_ were performed similar and comparable, whereas MinION_ASM_ performed worse in predicting the VFs. Therefore, one must interpret the data with care when studying the VFs solely using MinION_ASM_. Results for the current study are in contrast with previously published results for VFs detected in Shiga toxin-producing *E. coli*, where authors reported better performance for MinION_ASM_ over Illum_ASM_ [54]. These differences could be explained by using different assemblers or technology used for library preparation and sequencing. 

One must consider that AMR and VF profiles are not stable over the isolates and plasmid-mediated AMR genes can be horizontally transferred between isolates. Hence, identifying the exact number of AMR or VFs and comparing the results with reference samples might be challenging. Although here we included *E. coli* strain NCTC 13441 as a reference isolate and annotation results correlated well with the reference genome, this study was limited by the absence of reference genome for *K. pneumoniae*. 

Translating the current finding for long reads to the output from other platforms such as PacBio is challenging. Previous research showed that PacBio generated both longer and more accurate reads compared to ONT [55,56]. However, the applicability of the long-read sequencer is largely depending on the type of the research. For instance, it has been shown that ONT performance for quantitative analyses such as transcriptome studies was better than PacBio [55]. Furthermore, the superiority of ONT over PacBio for the rapid identification of pathogens has been shown previously [57]. Our data show that MinION data assembly is faster compared to both Illum_ASM_ and Hyb_ASM_. Our analyses indicated that all type of assemblies can be performed using a Linux machine with standard computational resources. We tested the elapsed time for the assembly of the reference genome (*E. coli* strain NCTC 13441). The data showed that the assembly took 1 h and 13 min for Hyb_ASM_ (using Unicycler), 40 min for Illum_ASM_ (using Unicycler) and 10 min for MinION_ASM_ (using Flye). A shorter turnaround time for assembly, in parallel with a shorter turnaround time for MinION sequencing as compared to Illumina, which we have previously shown [20], make MinION the favorable sequencing platform, especially for field and diagnostic research. 

In our previous research, we identified AMR genes and plasmids in clinical isolates, using solo plasmid assembly [58]. We concluded that results are heavily dependent on the database of choice therefore, Hyb_ASM_ might be a better approach for in depth analyses of WGS data. Although Hyb_ASM_ has been used to study hospital *Mycobacterium chelonae* infections [59], extraintestinal pathogenic *E. coli* isolates [52] and one pan-drug-resistant *K. pneumoniae* isolate [60], the application of Hyb_ASM_ for studying pathogenic factors in clinical samples is somewhat limited. In the current study, Hyb_ASM_ showed reliable results for studying the clinical samples, specifically for the mixed culture as compared to Illum_ASM_ and MinION_ASM_. Compared to MinION_ASM_ and Illum_ASM_, downstream analyses using Hyb_ASM_ was accurate and more informative. These findings are in agreement with previous research, where the authors suggested that combining the ONT and PacBio data with Illumina data and generating a hybrid assembly greatly improved the accuracy and mappability of long reads [56]. Promising results of Hyb_ASM_ have facilitated the annotation of clinically relevant genomic elements. Interestingly, similar conclusions using Hyb_ASM_ have previously been drawn for assemblies from environmental samples [18,61] and clinical samples [62,63]. 

Taken all together, prior to sequencing, one might consider the experiment need; for Hyb_ASM_, the same sample needs to be sequenced in two different platforms, which is resource demanding. The MinION sequencer demonstrated an acceptable performance to study the genome of clinical isolates. However, solely long reads might not be ideal for predicting gene variants, point mutation, and virulence factors. Moreover, it seems possible that the source of the samples and the method for library preparation for long-read sequencing might play an important role in the quality and the amount of collected data. Nanopore technology is evolving, and one may be optimistic that the current weaknesses could be overcome with technology improving in the near future. Despite all uncertainty regarding the long reads, and although we did not use exactly the same DNA for both platforms, the data showed that even a low quantity of long reads in combination with short reads could greatly improve the assembly. Therefore, Hyb_ASM_ should be considered a successful approach to overcome uncertainty caused by Oxford Nanopore technology. Considering the cost of experiments, extended data analyses, and the possibility of mixed infections in clinical samples, application of Hyb_ASM_ for the study the genome, AMR and VF genes, and potential plasmid, could be justified. Otherwise, Illumina assembly could be considered as sufficient.

## 5. Conclusions

In conclusion, Hyb_ASM_ is a good approach for the in-depth analysis of clinically relevant samples, including blood cultures, as demonstrated here. It is recommended to benefit from the advantages of Hyb_ASM_ for genome study of complicated mixed isolates, the in-depth determination of pathogenicity and epidemiological studies. The present findings emphasize the fact that selecting the appropriate approach for sequencing and assembly could have great impact on the results and could indirectly shorten the times required to detect pathogenicity factors in clinical settings.

## Figures and Tables

**Table 1:** An overview of basic sequence information statistics and quality of reads after trimming and filtering. The mixed culture was obtained from the co-culturing of *E. coli* 4 and *K. pneumoniae* 5 isolates. Coverage of *E. coli* isolates was calculated by dividing the number of bp in each read over the number of bp in reference genome (*E. coli* NCTC 13441). Coverage of *K. pneumoniae* isolates was calculated by dividing the number of bp reads over the number of bp reads in the *K. pneumoniae* reference genome (median genome size of all *K. pneumoniae* isolates in NCBI database). Coverage of mixed culture sample was calculated by dividing the number of bp in mix culture sample over the sum of pb of *E. coli* NCTC 13441 and median genome size of all *K. pneumoniae* isolates in NCBI database.

	MinION Long Reads					Illumina Short Reads		
	Read Length N50 (bp)	Mean Read Quality (Q)	Number of Reads	Total bp	Coverage (X)	Number of Reads	Total bp	Coverage (X)
*E. coli* 1	2520	11.4	63,036	92,626,571	17.4	670,985	91,989,902	17.2
*E. coli* 2	1466	11.3	67,331	88,553,163	16.6	597,154	141,802,031	26.6
*E. coli* 3	1384	11.5	41,103	39,979,342	7.5	1,786,471	396,985,933	74.4
*E. coli* 4	5956	9.8	81,317	256,369,935	48.0	1,419,582	353,790,894	66.3
*E. coli* (mean ± SD)	2832 ± 2147	11 ± 0.8	63,197 ± 16,669	119,382,253 ± 94,404,708	22.4 ± 18	1,118,548 ± 579,915	246,142,190 ± 151,648,580	46.1 ± 28
*K. pneumoniae* 1	1428	11.3	13,694	25,125,702	4.5	889,410	222,836,627	39.8
*K. pneumoniae* 2	7302	11.5	199,822	859,067,656	153.5	559,060	131,573,009	23.5
*K. pneumoniae* 3	4250	11.5	51,624	136,843,964	24.5	744,422	111,911,073	20.0
*K. pneumoniae* 4	2044	9.9	329,042	375,495,020	67.1	1,302,920	313,973,441	56.1
*K. pneumoniae* 5	3941	9.3	48,463	64,316,017	11.5	712,218	178,050,866	31.8
*K. pneumoniae* (mean ± SD)	3793 ± 2302	11 ± 1	128,529 ± 133,041	292,169,672 ± 344,844,995	52.2 ± 62	841,606 ± 283,335	191,669,003 ± 80,759,064	34.2 ± 14
Mixed culture sample	4200	9.8	143,076	387,311,832	35.4	2,131,800	531,841,759	48.7

**Table 2:** An overview of statistics for different *E. coli* and *K. pneumoniae* assemblies produced by the top-performing assemblers. Illum_ASM_ was produced using Unicycler, MinION_ASM_ using Flye and Hyb_ASM_ created using Unicycler. The top values are highlighted in bold. The mixed culture was obtained from the co-culturing of *E. coli* 4 and *K. pneumoniae* 5 isolates. Numbers show the average ± SD.

		Number of Dead Ends	Number of Contigs	Total Length (bp)	N50 (bp)
*E. coli*	Illum_ASM_	4 ± 4	138 ± 90	5,232,982 ± 335,084	225,244 ± 82,435
	MinION_ASM_	77 ± 94	**49 ± 47**	3,870,499 ± 2,664,510	343,234 ± 504,598
	Hyb_ASM_	**4 ± 2**	50 ± 28	**5,317,286 ± 426,129**	**1,005,273 ± 476,961**
*K. pneumoniae*	Illum_ASM_	10 ± 7	78 ± 13	5,577,253 ± 181,931	247,095 ± 138,114
	MinION_ASM_	44 ± 48	35 ± 32	4,694,978 ± 2,235,357	1,996,101 ± 2,279,327
	Hyb_ASM_	**1 ± 3**	**20 ± 17**	**5,648,111 ± 211,443**	**3,880,248 ± 2,149,256**
Mixed culture sample	Illum_ASM_	2	371	11,193,506	147,235
	MinION_ASM_	65	120	**11,827,293**	344,695
	Hyb_ASM_	**0**	**117**	11,495,693	**1,245,846**

**Table 3:** Average values for annotating the genomic features of different assemblies from monocultures and mixed cultures of *E. coli* and *K. pneumoniae* isolates. Illum_ASM_ was produced using Unicycler, MinION_ASM_ using Flye and Hyb_ASM_ was created using Unicycler. The mixed culture was obtained from the co-culturing of *E. coli* 4 and *K. pneumoniae* 5 isolates. Numbers show the average ± SD.

		CDS	rRNA	tRNA	tmRNA
*E. coli*	Illum_ASM_	4952 ± 392	5 ± 1	83 ± 5	1 ± 0
	MinION_ASM_	6715 ± 4615	12 ± 10	63 ± 44	1 ± 1
	Hyb_ASM_	5042 ± 532	15 ± 9	88 ± 11	1 ± 0
*K. pneumoniae*	Illum_ASM_	5201 ± 185	4 ± 1	79 ± 1	1 ± 0
	MinION_ASM_	8120 ± 3933	20 ± 10	67 ± 36	1 ± 1
	Hyb_ASM_	5261 ± 217	21 ± 8	84 ± 4	1 ± 0
Mixed culture sample	Illum_ASM_	10,660	8	164	2
	MinION_ASM_	20,158	47	181	2
	Hyb_ASM_	10,995	44	184	2

## Data Availability

The data that support the findings of this study are available from The European Nucleotide Archive (ENA) under primary accession number PRJEB45084 and secondary accession number ERP129212. An overview of submitted reads is provided in Supplementary Table S1.

## Supplementary Materials

The following are available online at https://www.mdpi.com/article/10.3390/microorganisms9122560/s1. Figure S1_Assembly graphs for Illum_ASM_; Figure S2_Assembly graphs for MinION_ASM._; Figure S3_Assembly graphs for Hyb_ASM._; Figure S4_Assembly graphs for assemblies from top performed assemblers; Figure S5_BUSCO graphs for all assemblies produced by different assemblers.; Table S1_Isolates ID, ENA accession number, source, and culture method; Table S2_Reference genome information; Table S3_Assembley statistics for all assemblies produced by all assemblers; Table S4_Assembly statistics for assemblies produced by top performing assemblers; Table S5_Summary of Prokka annotation results for top performing assemblers; Table S6_Full Prokka table for top performing assemblers; Table S7_Identified plasmids in assemblies produced by top performing assemblers; Table S8_Identified AMR genes in assemblies produced by top performing assemblers; Table S9_Identified VFs in assemblies produced by top performing assemblers; Table S10_Mixed cultured results.
