# Supplementary material for: Hybrid Assembly Provides Improved Resolution of Plasmids, Antimicrobial Resistance Genes, and Virulence Factors in Escherichia coli and Klebsiella pneumoniae Clinical Isolates
Source: Microorganisms. 2021 Dec 10;9(12):2560. doi: 10.3390/microorganisms9122560 (PMC8704702; doi:10.3390/microorganisms9122560)
Supplement: Supplementary file 1 [file microorganisms-09-02560-s001.zip › Supplementary Figure S1_Assembly graphs for IllumASM.pptx]

## Slide 1
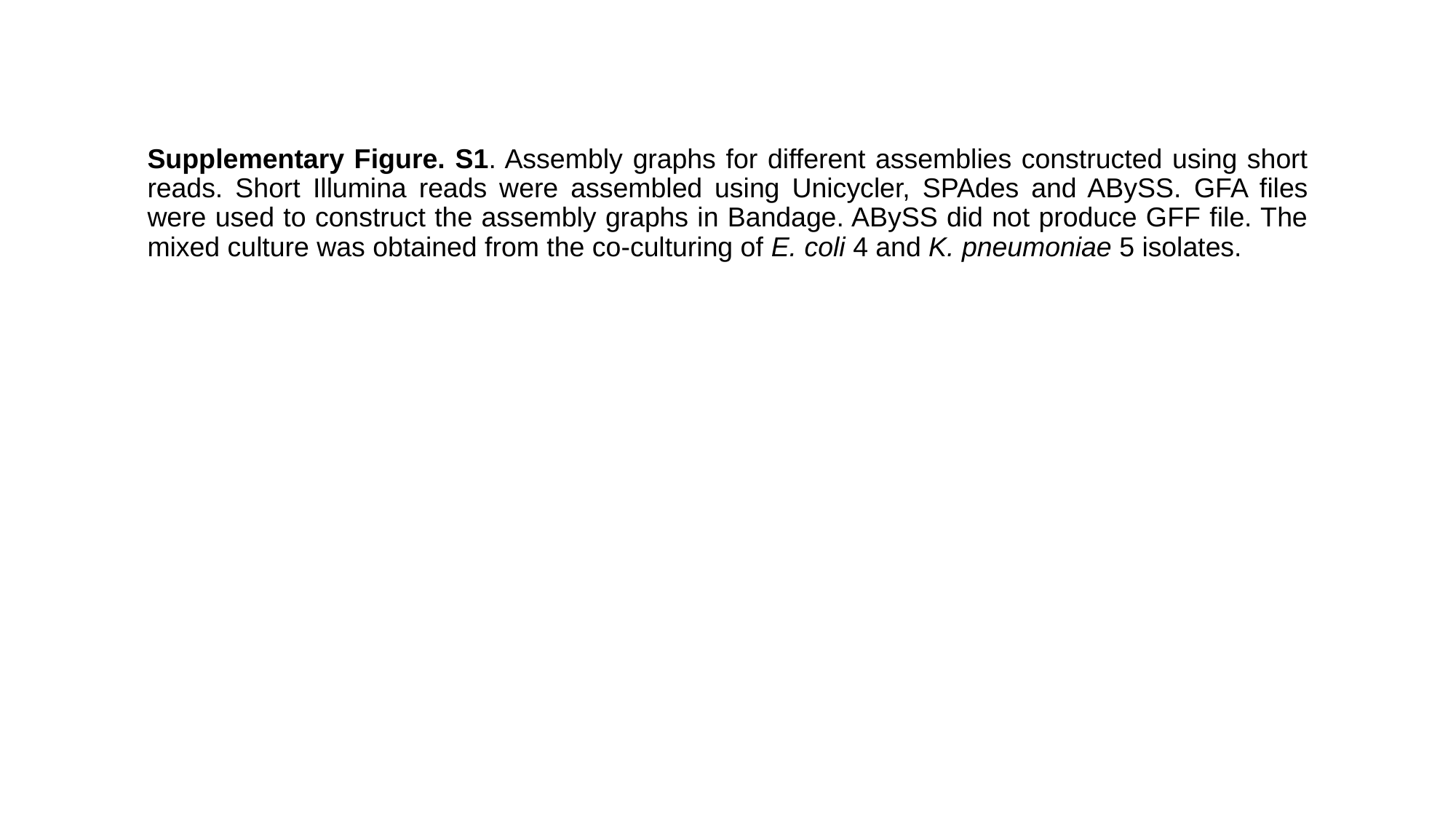

Supplementary Figure. S1. Assembly graphs for different assemblies constructed using short reads. Short Illumina reads were assembled using Unicycler, SPAdes and ABySS. GFA files were used to construct the assembly graphs in Bandage. ABySS did not produce GFF file. The mixed culture was obtained from the co-culturing of E. coli 4 and K. pneumoniae 5 isolates.

## Slide 2
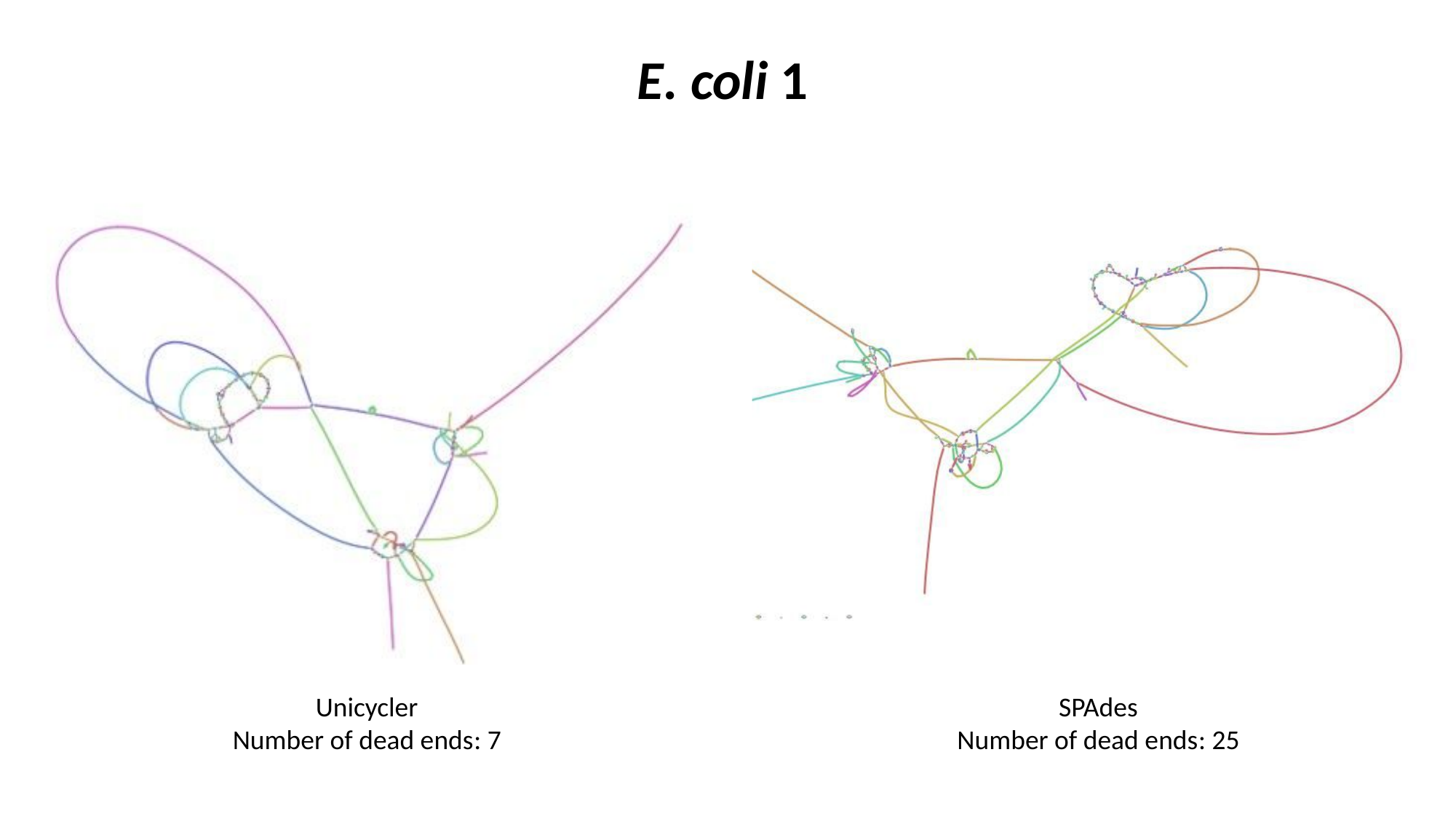

E. coli 1
Unicycler
Number of dead ends: 7
SPAdes
Number of dead ends: 25

## Slide 3
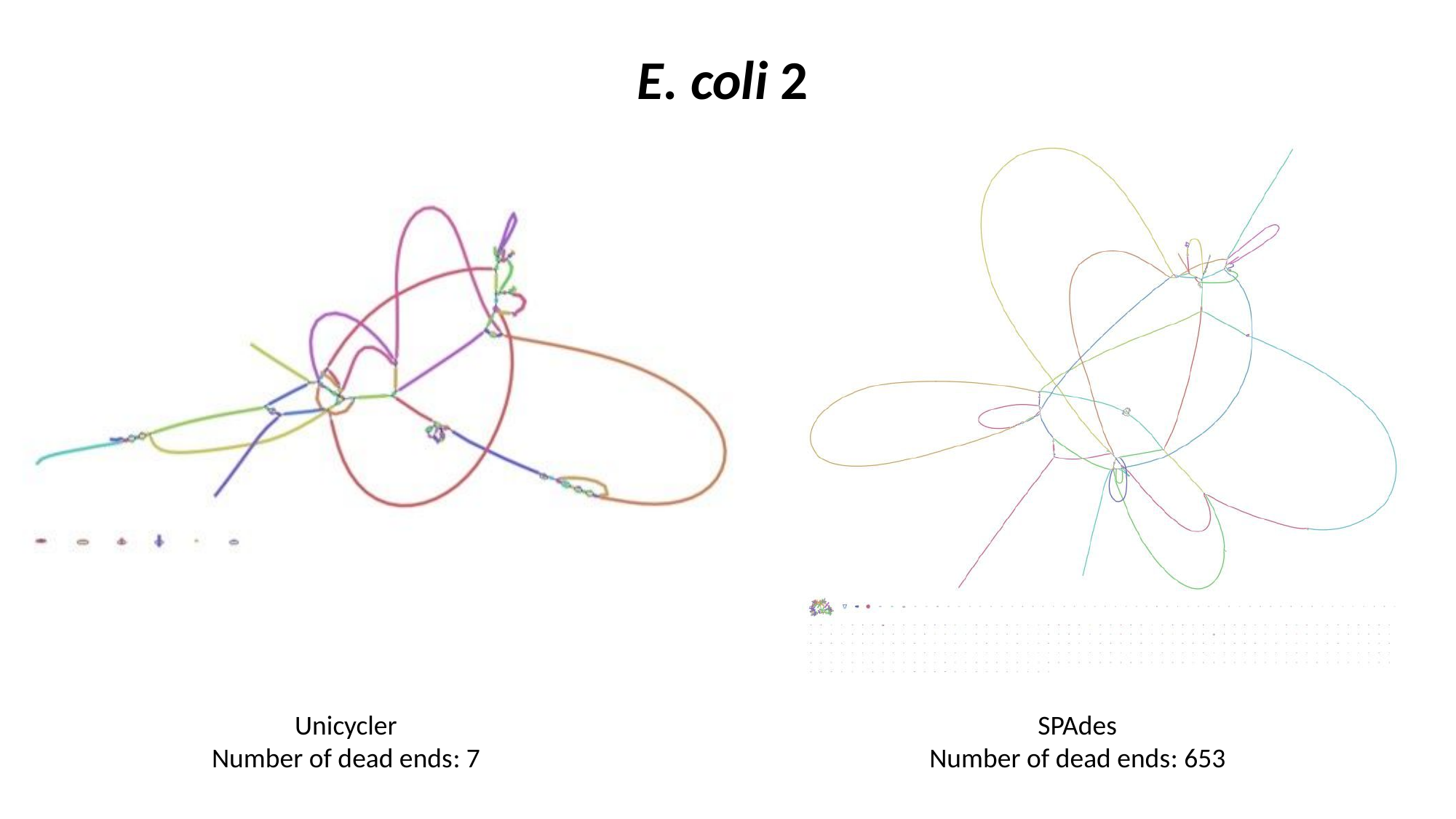

E. coli 2
Unicycler
Number of dead ends: 7
SPAdes
Number of dead ends: 653

## Slide 4
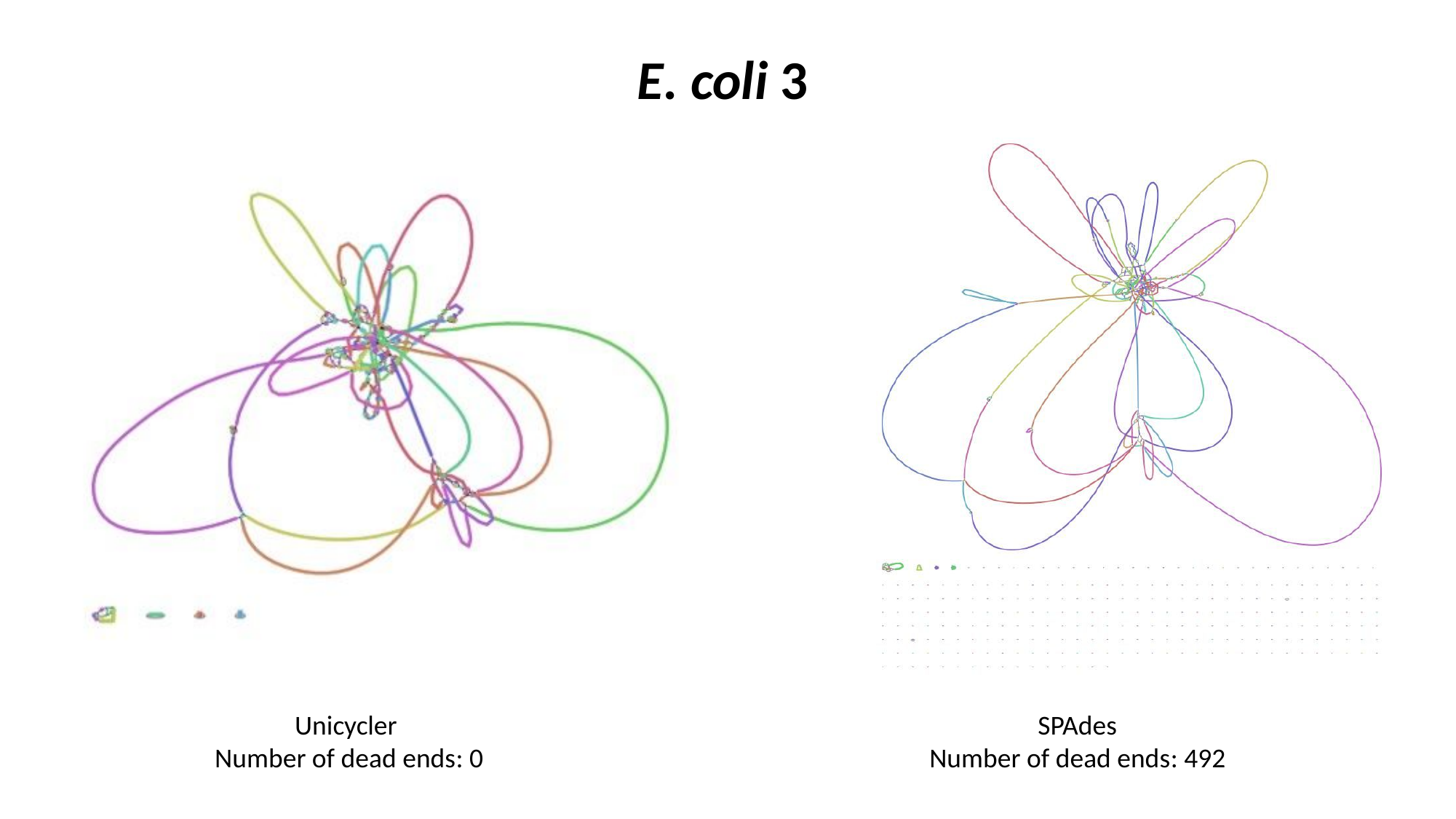

E. coli 3
Unicycler
Number of dead ends: 0
SPAdes
Number of dead ends: 492

## Slide 5
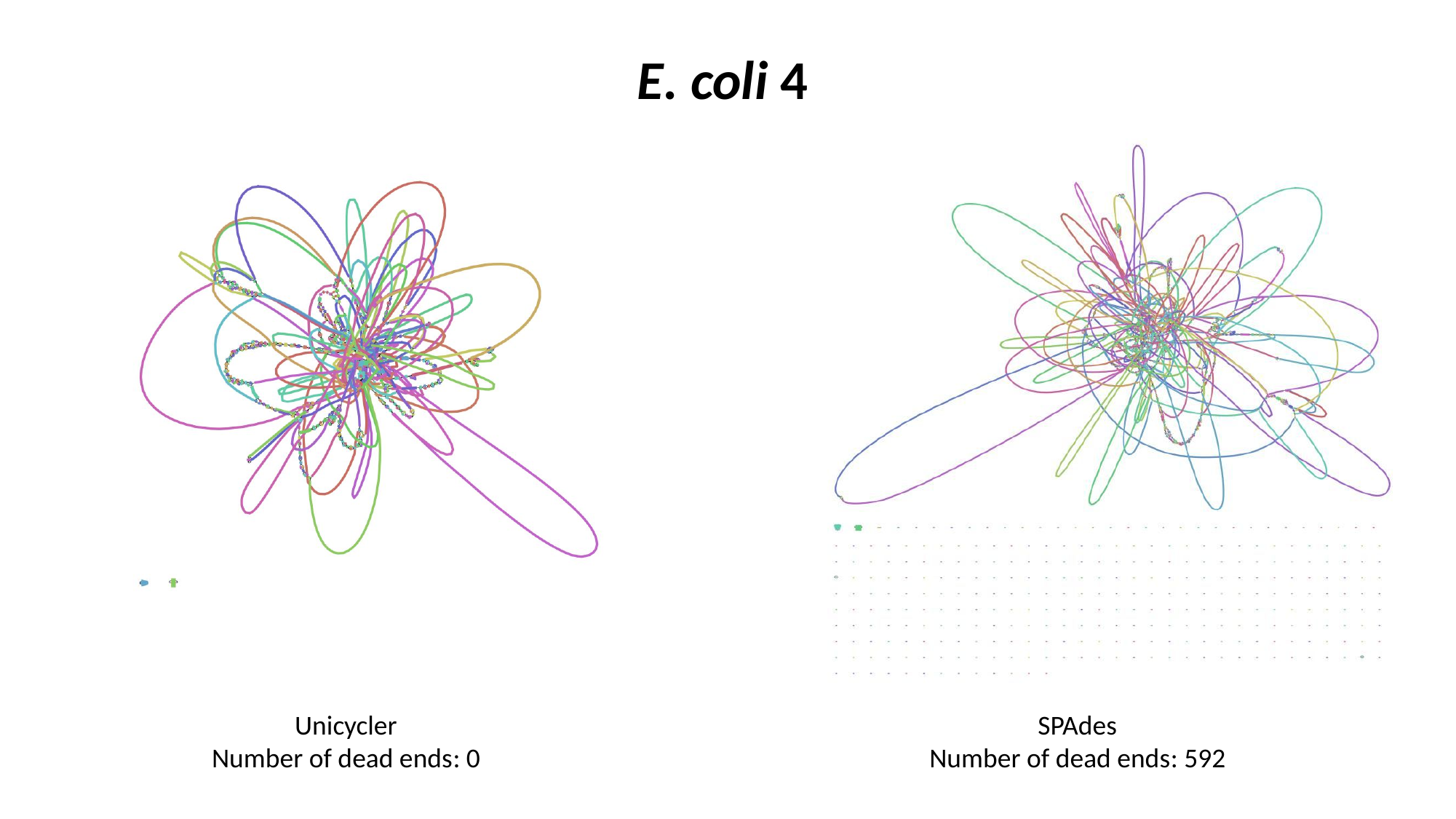

E. coli 4
Unicycler
Number of dead ends: 0
SPAdes
Number of dead ends: 592

## Slide 6
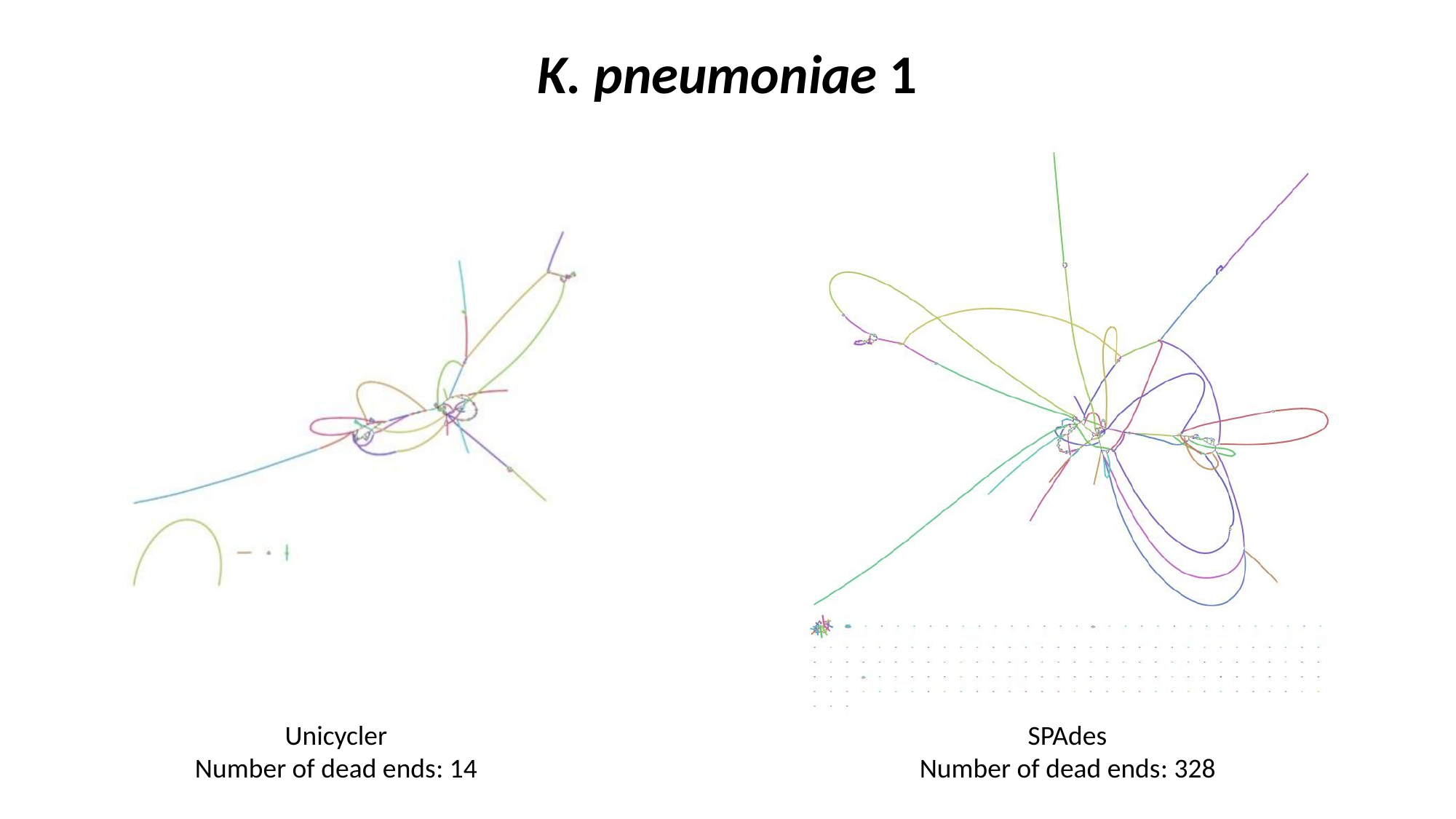

K. pneumoniae 1
Unicycler
Number of dead ends: 14
SPAdes
Number of dead ends: 328

## Slide 7
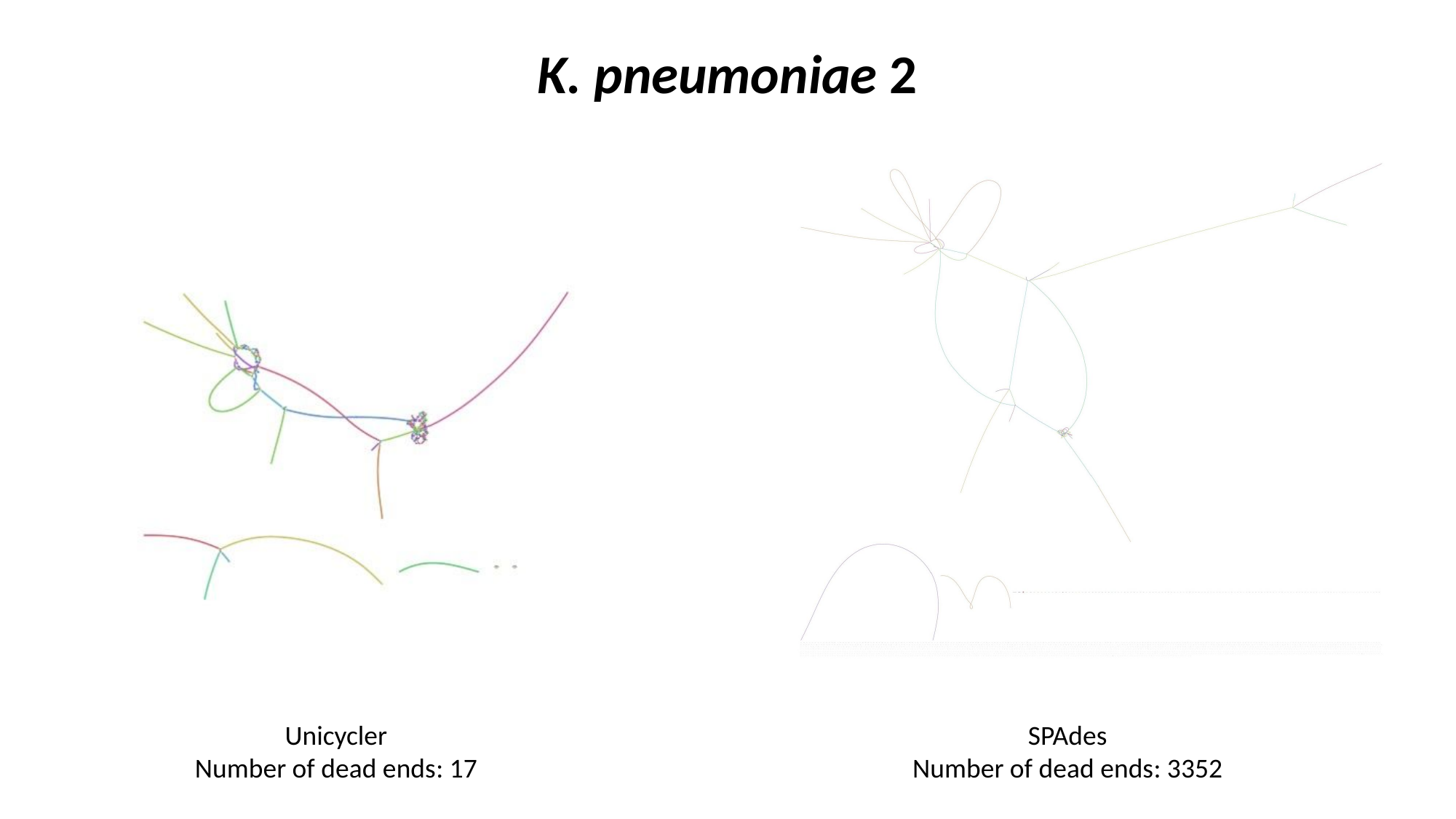

K. pneumoniae 2
Unicycler
Number of dead ends: 17
SPAdes
Number of dead ends: 3352

## Slide 8
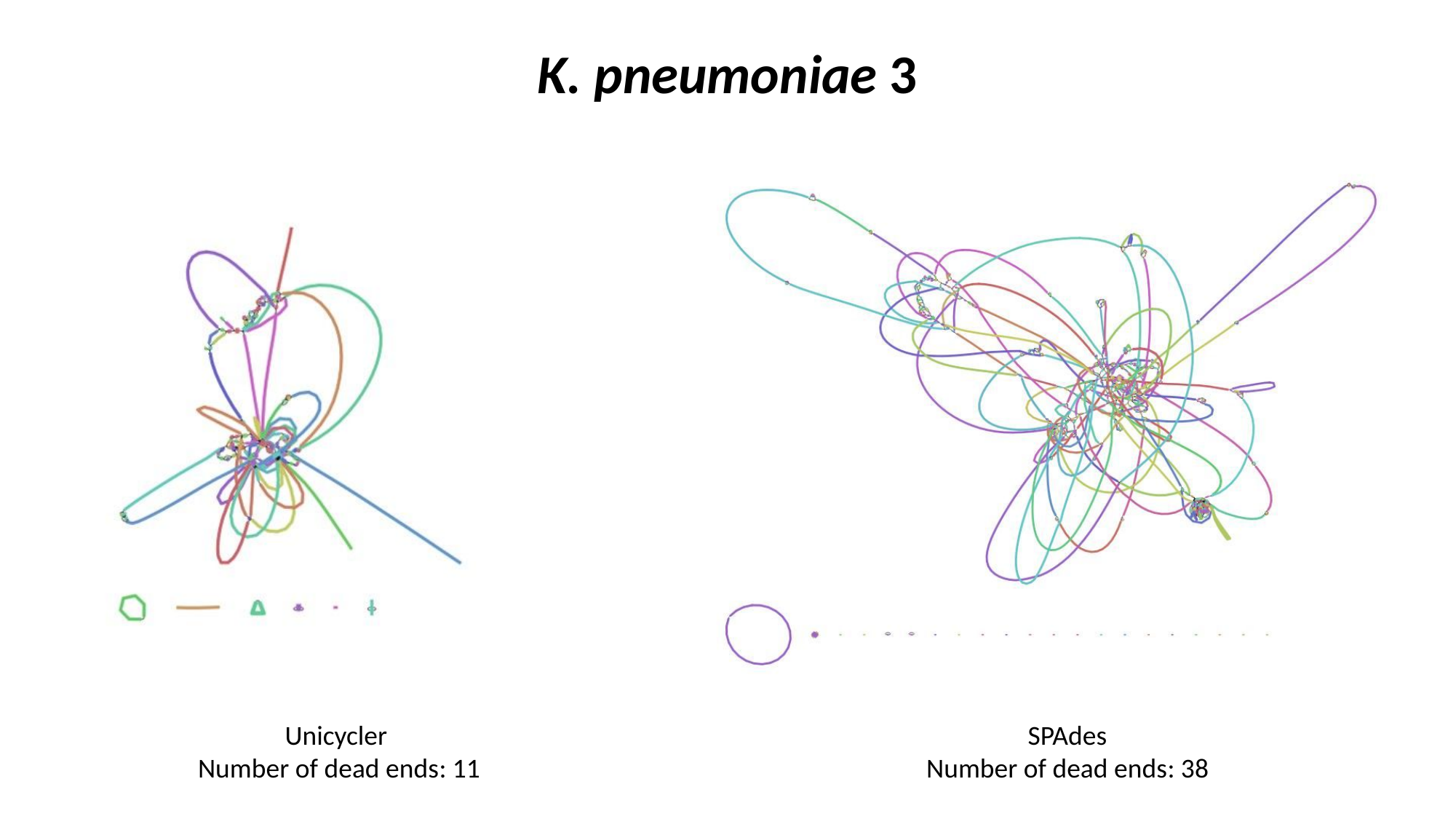

K. pneumoniae 3
Unicycler
Number of dead ends: 11
SPAdes
Number of dead ends: 38

## Slide 9
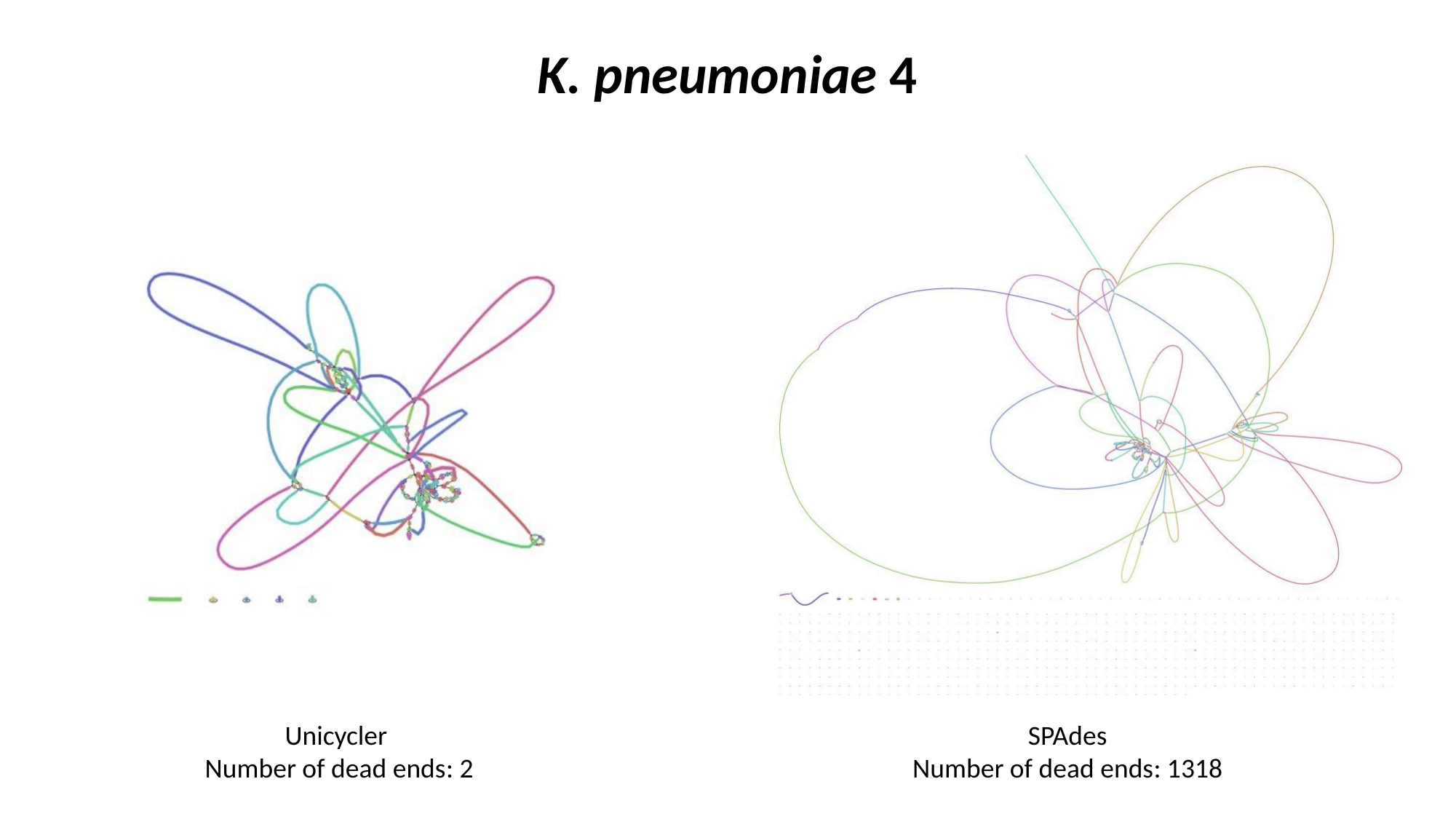

K. pneumoniae 4
Unicycler
Number of dead ends: 2
SPAdes
Number of dead ends: 1318

## Slide 10
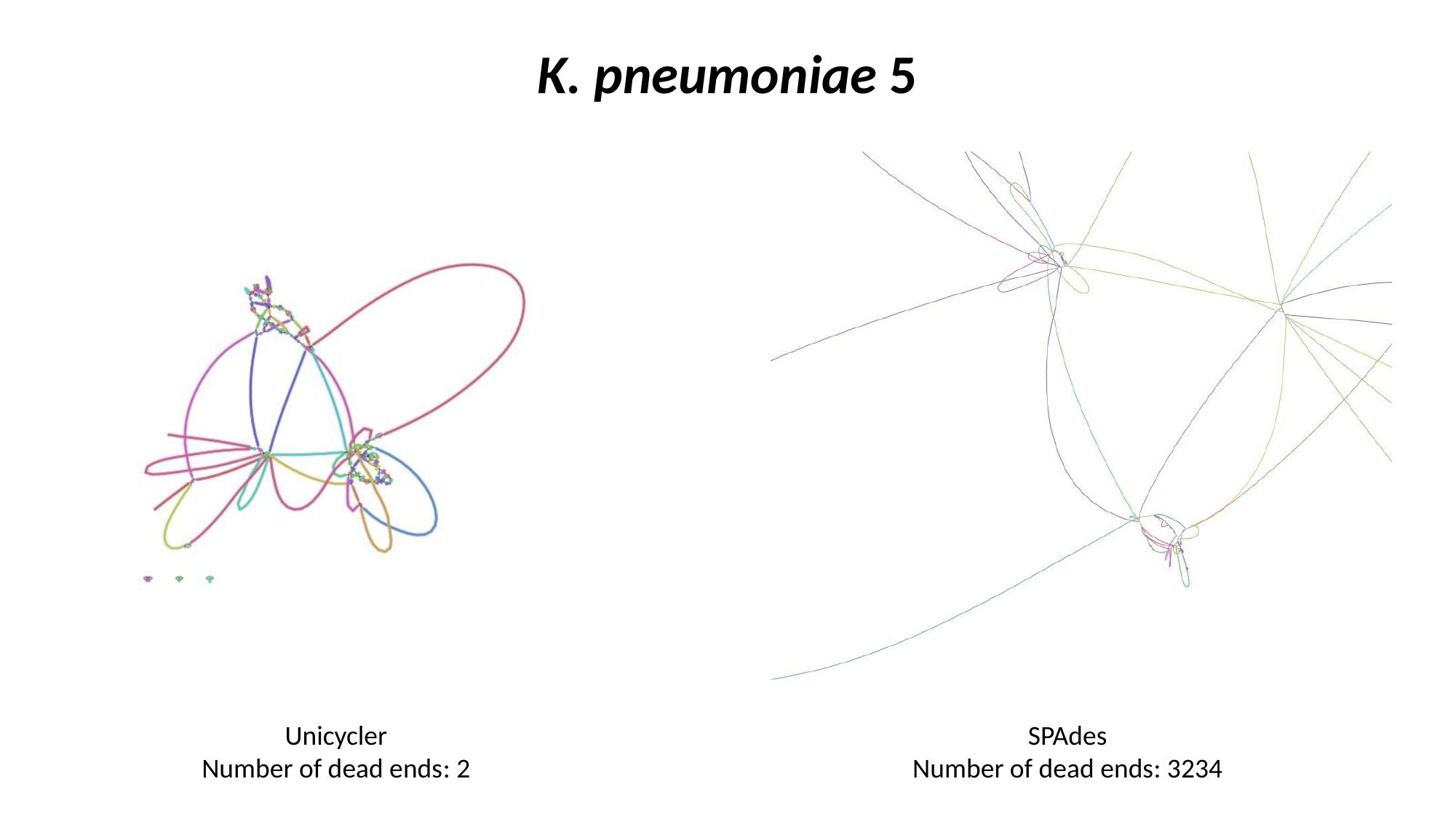

K. pneumoniae 5
Unicycler
Number of dead ends: 2
SPAdes
Number of dead ends: 3234

## Slide 11
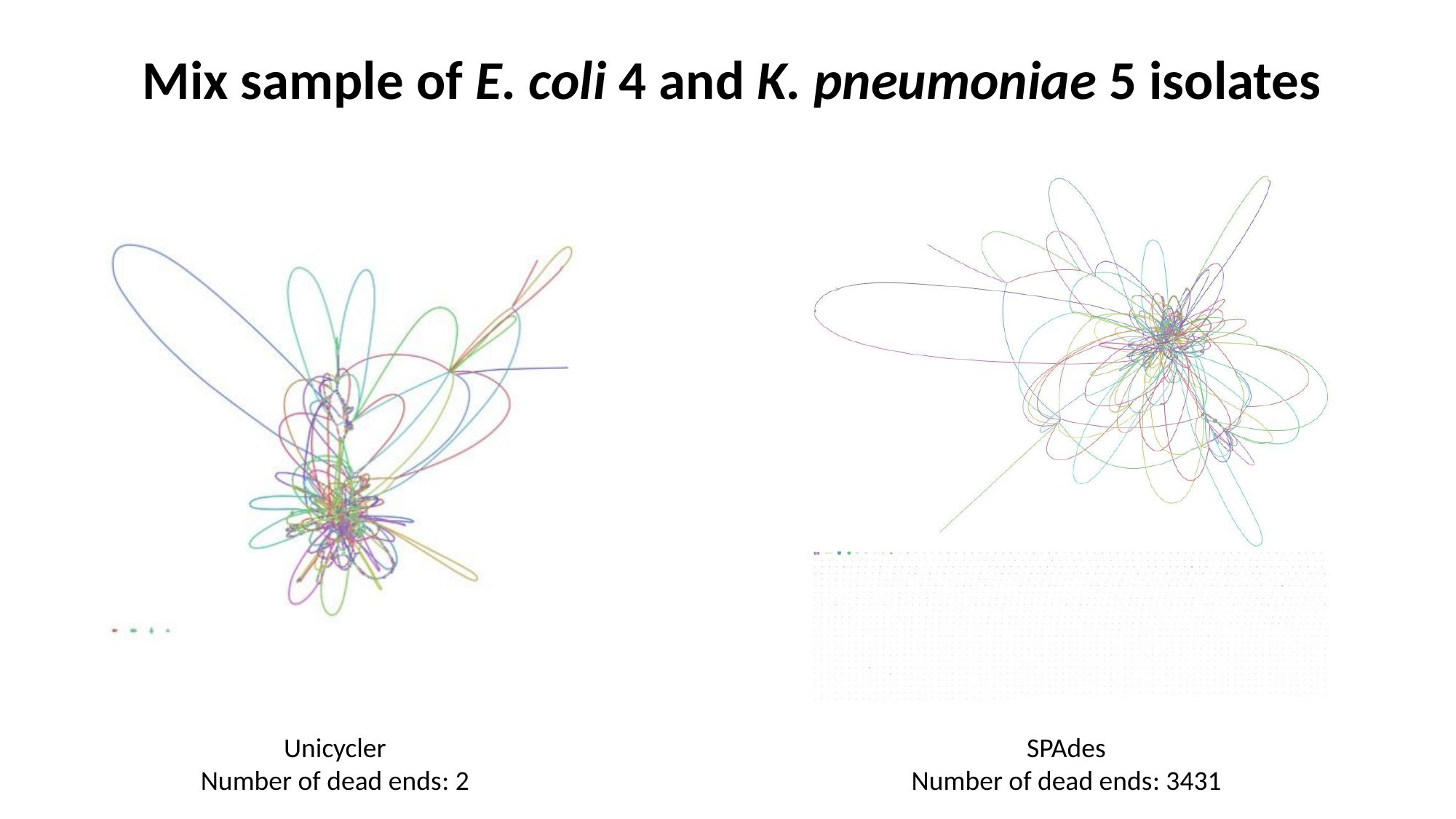

Mix sample of E. coli 4 and K. pneumoniae 5 isolates
Unicycler
Number of dead ends: 2
SPAdes
Number of dead ends: 3431
