# Supplementary material for: Hybrid Assembly Provides Improved Resolution of Plasmids, Antimicrobial Resistance Genes, and Virulence Factors in Escherichia coli and Klebsiella pneumoniae Clinical Isolates
Source: Microorganisms. 2021 Dec 10;9(12):2560. doi: 10.3390/microorganisms9122560 (PMC8704702; doi:10.3390/microorganisms9122560)
Supplement: Supplementary file 1 [file microorganisms-09-02560-s001.zip › Supplementary Figure S2_Assembly graphs for MinIONASM.PPTX]

## Slide 1
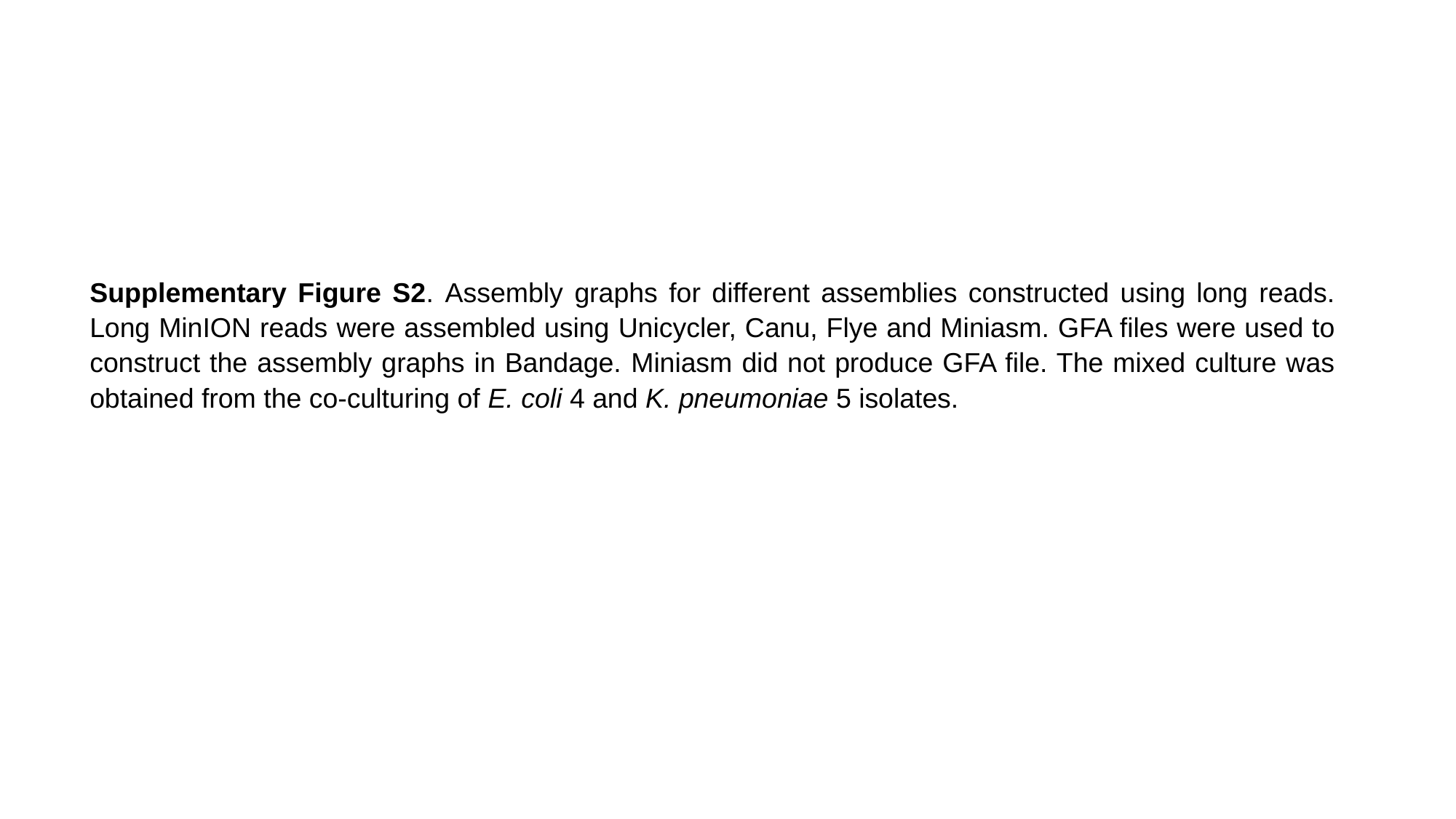

Supplementary Figure S2. Assembly graphs for different assemblies constructed using long reads. Long MinION reads were assembled using Unicycler, Canu, Flye and Miniasm. GFA files were used to construct the assembly graphs in Bandage. Miniasm did not produce GFA file. The mixed culture was obtained from the co-culturing of E. coli 4 and K. pneumoniae 5 isolates.

## Slide 2
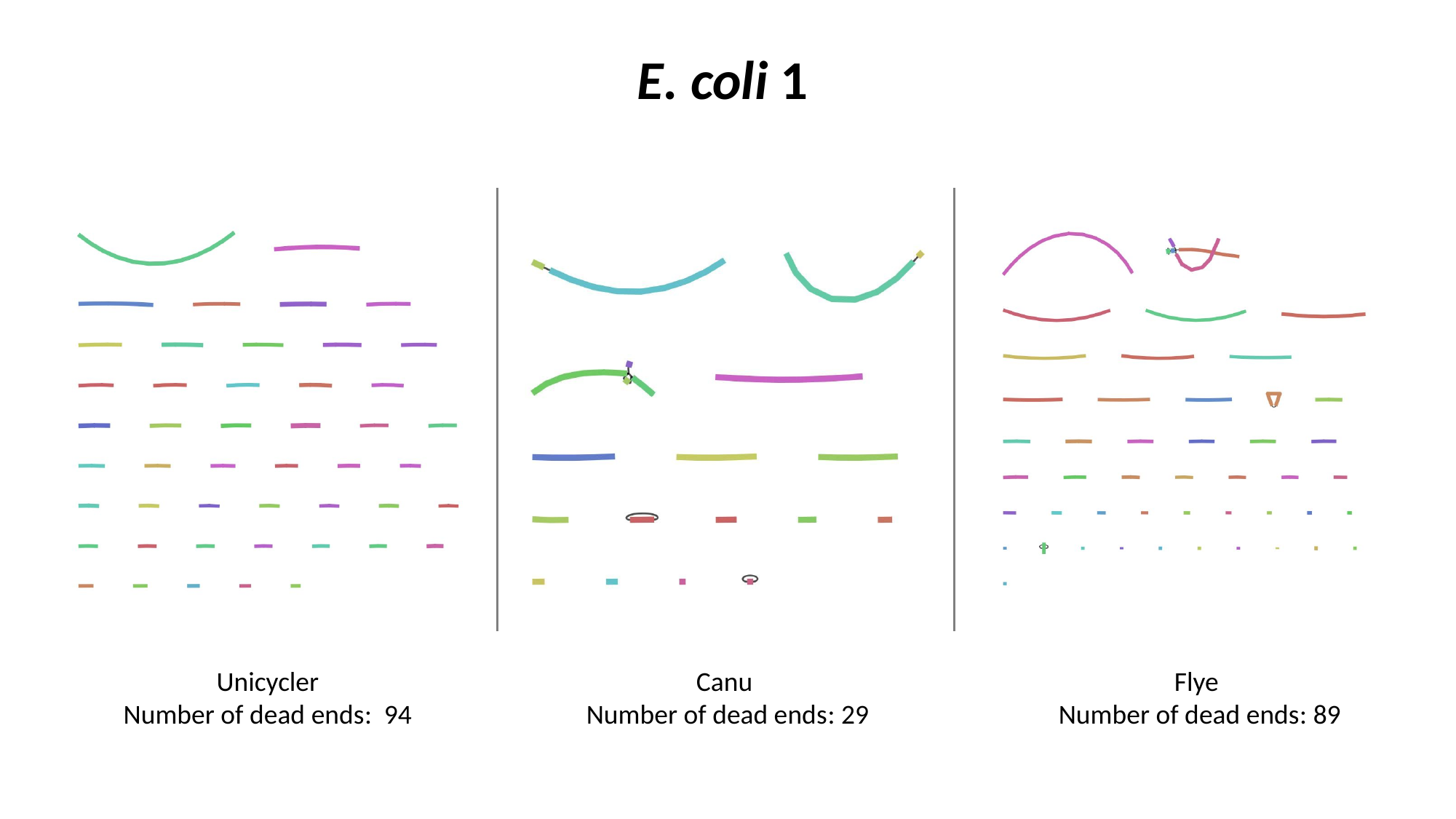

E. coli 1
Flye
Number of dead ends: 89
Unicycler
Number of dead ends: 94
Canu
Number of dead ends: 29

## Slide 3
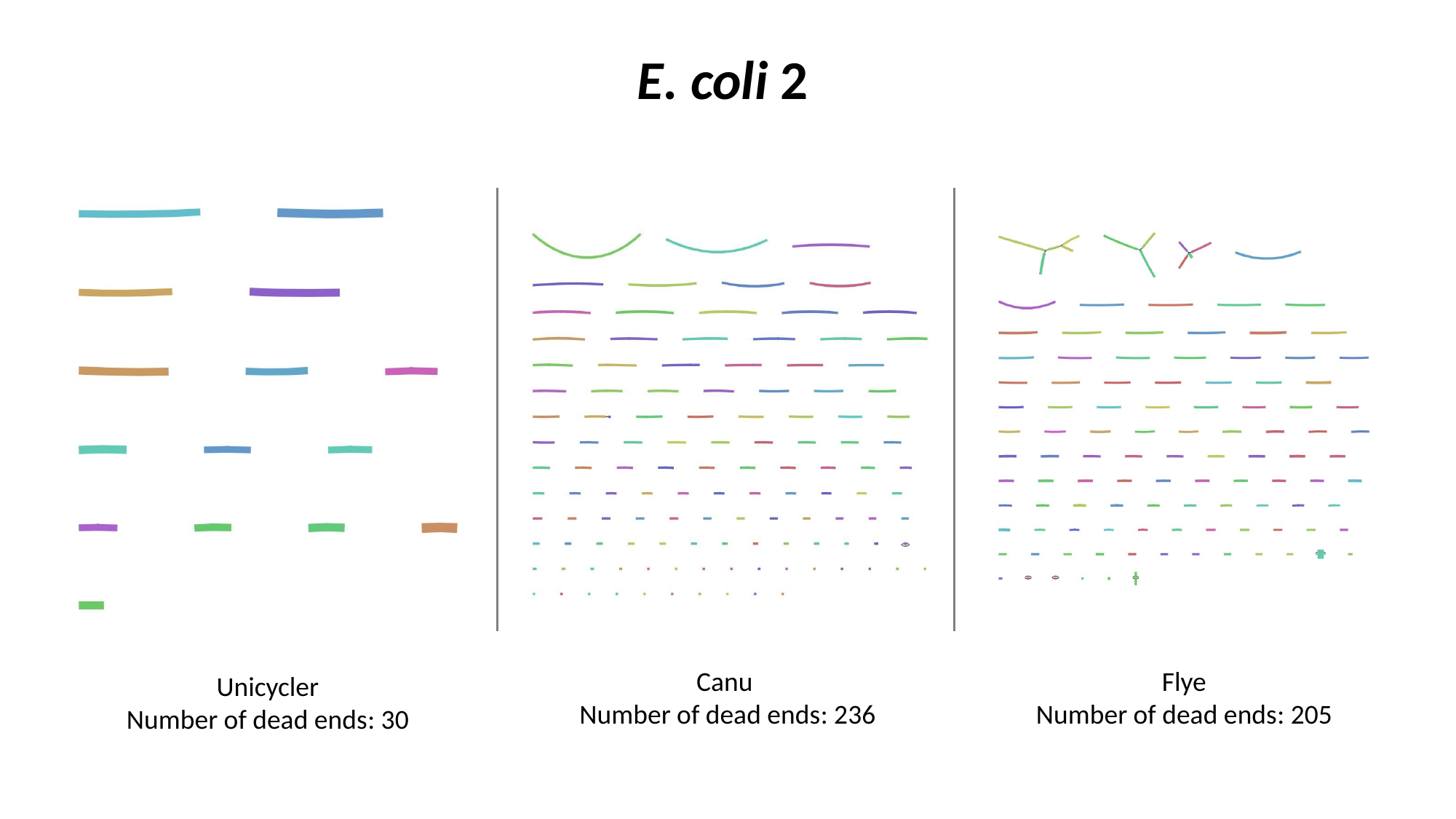

E. coli 2
Flye
Number of dead ends: 205
Canu
Number of dead ends: 236
Unicycler
Number of dead ends: 30

## Slide 4
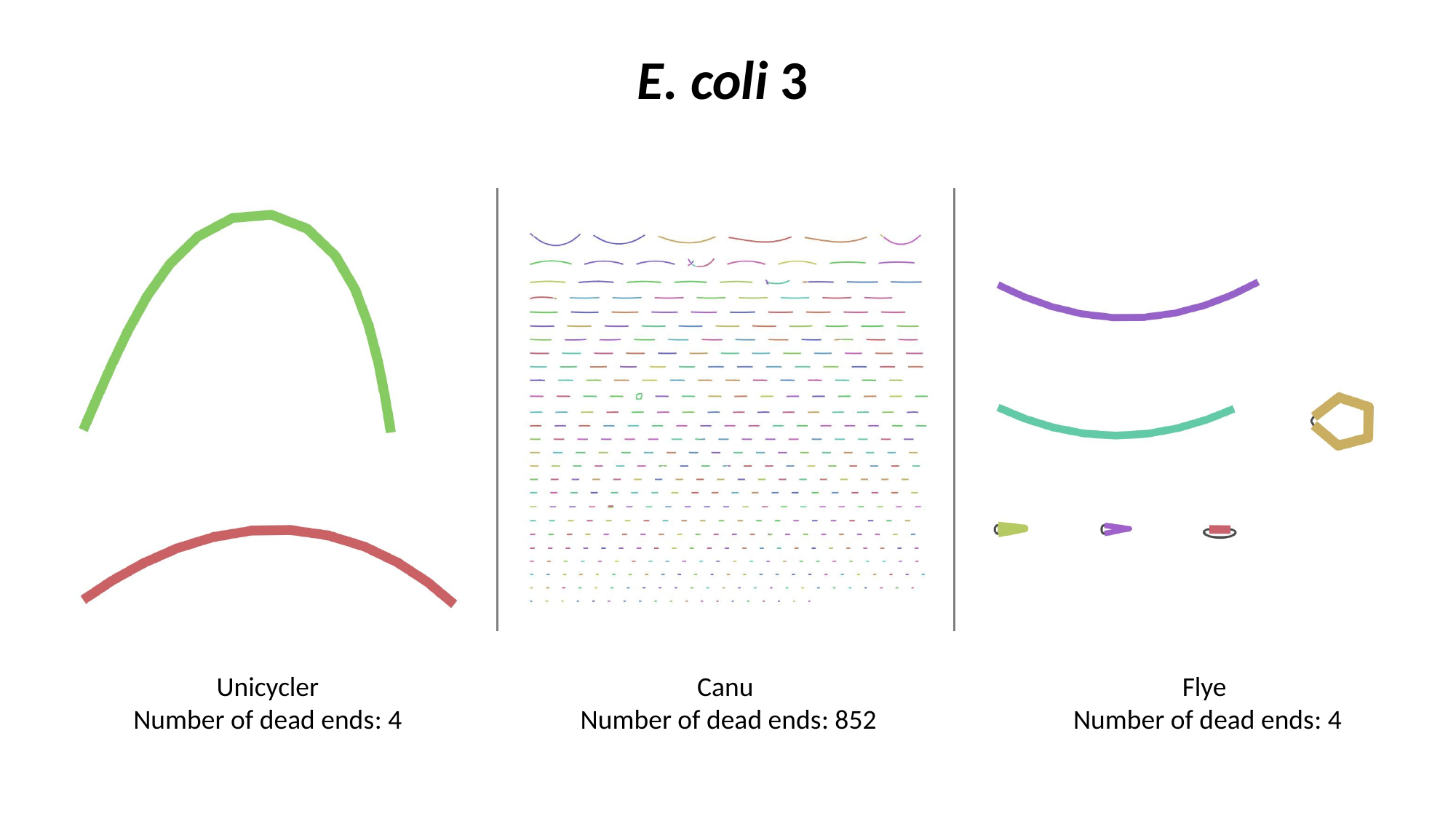

E. coli 3
Flye
Number of dead ends: 4
Canu
Number of dead ends: 852
Unicycler
Number of dead ends: 4

## Slide 5
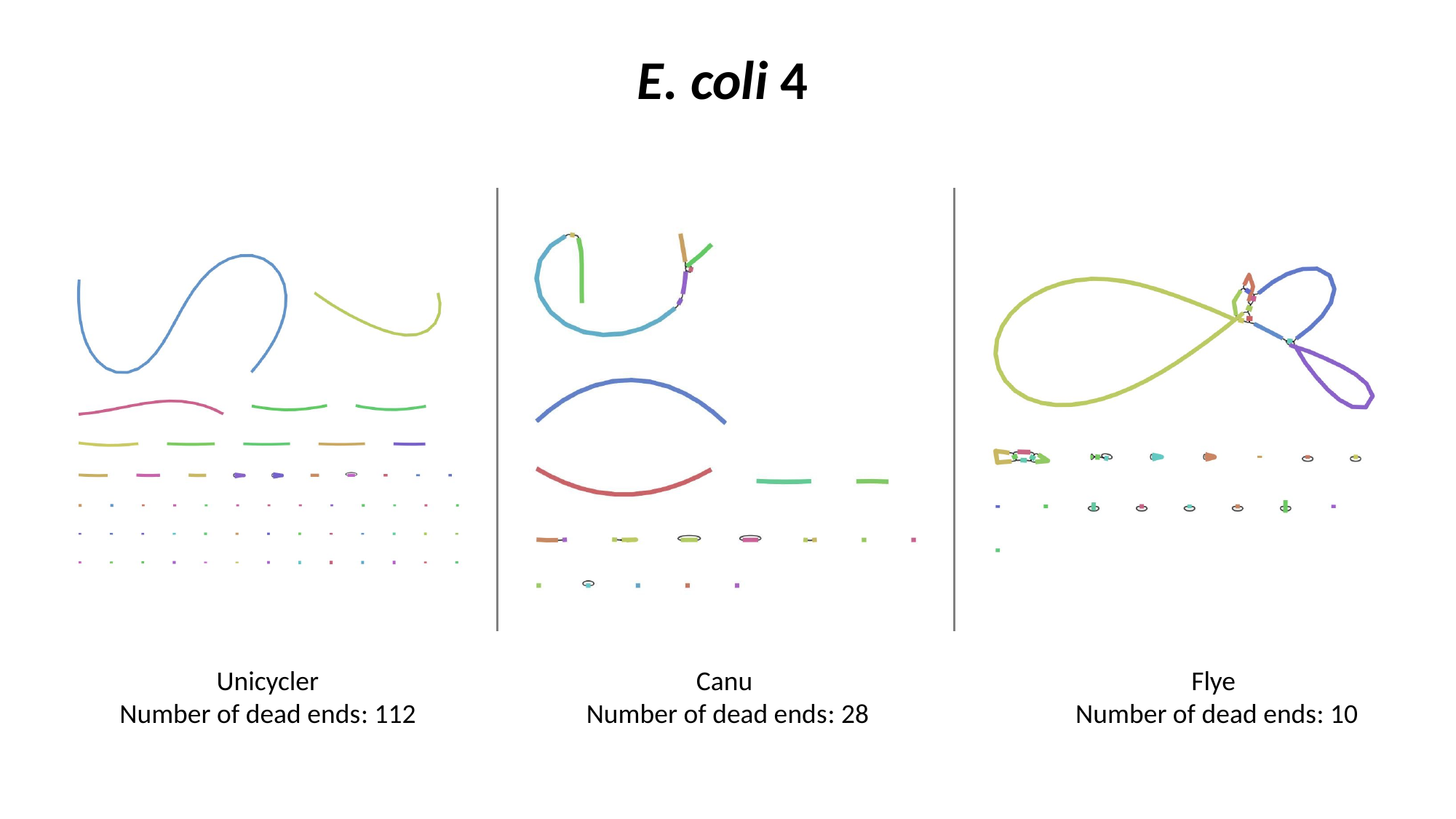

E. coli 4
Flye
Number of dead ends: 10
Unicycler
Number of dead ends: 112
Canu
Number of dead ends: 28

## Slide 6
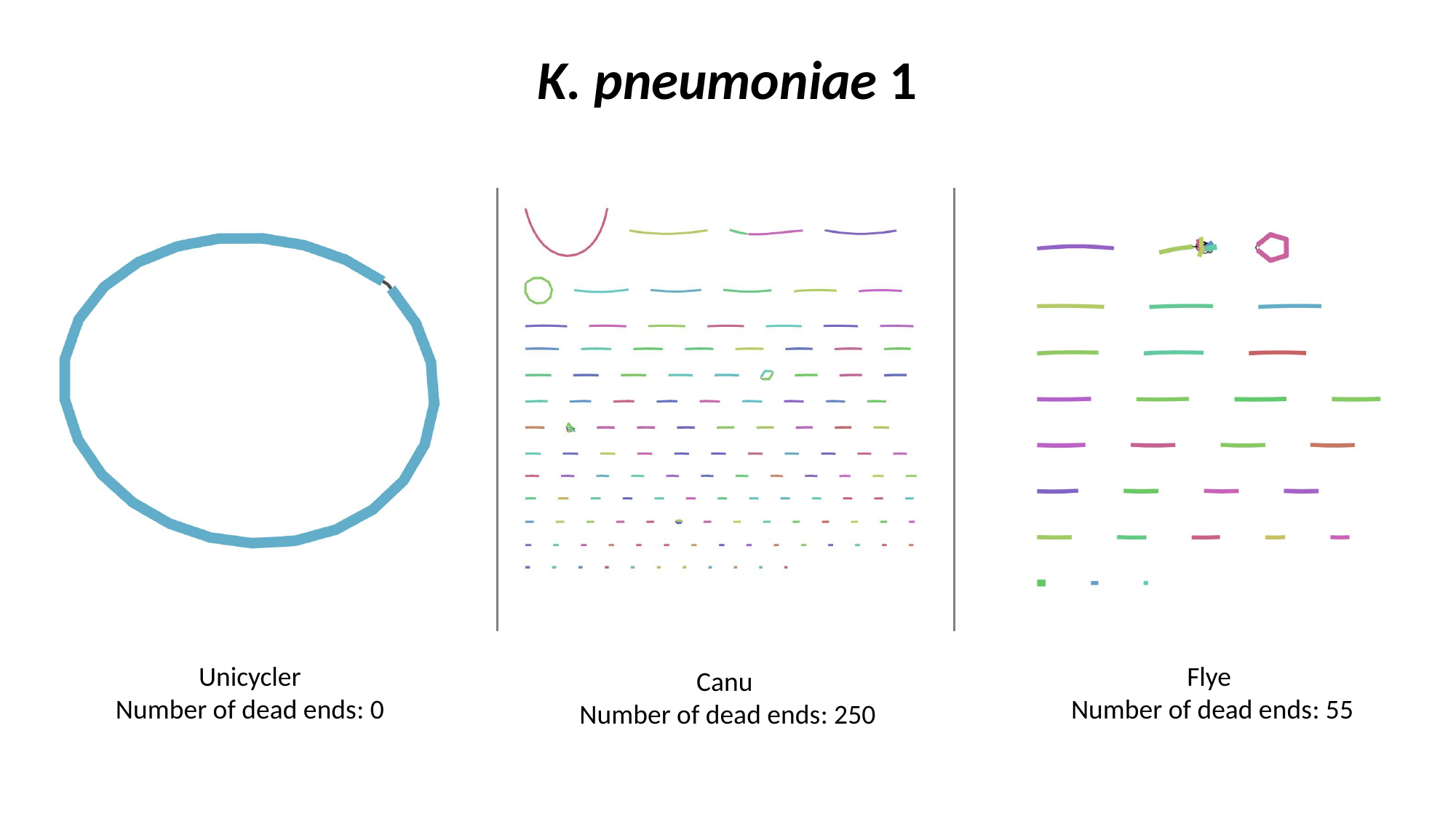

K. pneumoniae 1
Unicycler
Number of dead ends: 0
Flye
Number of dead ends: 55
Canu
Number of dead ends: 250

## Slide 7
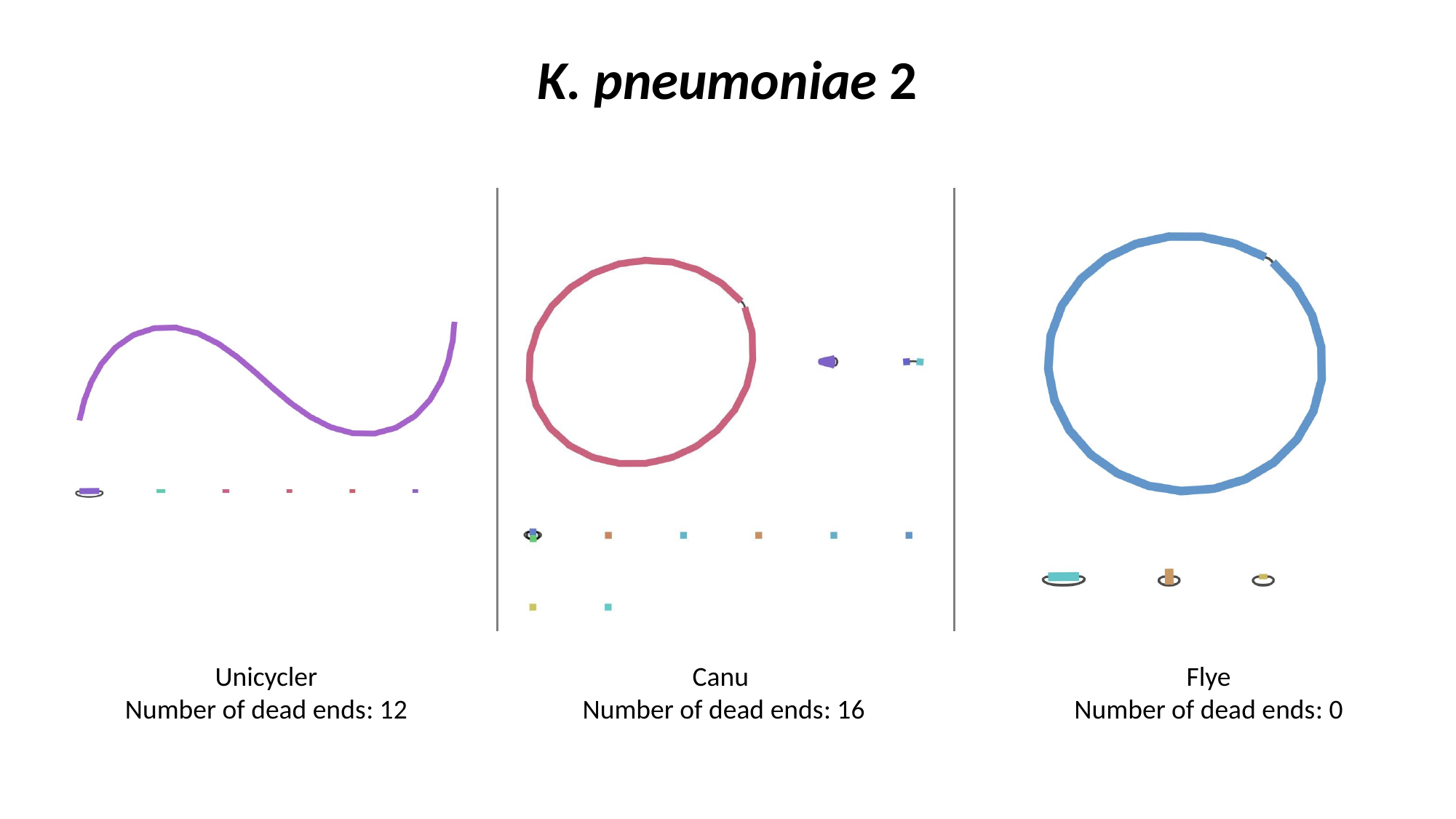

K. pneumoniae 2
Flye
Number of dead ends: 0
Canu
Number of dead ends: 16
Unicycler
Number of dead ends: 12

## Slide 8
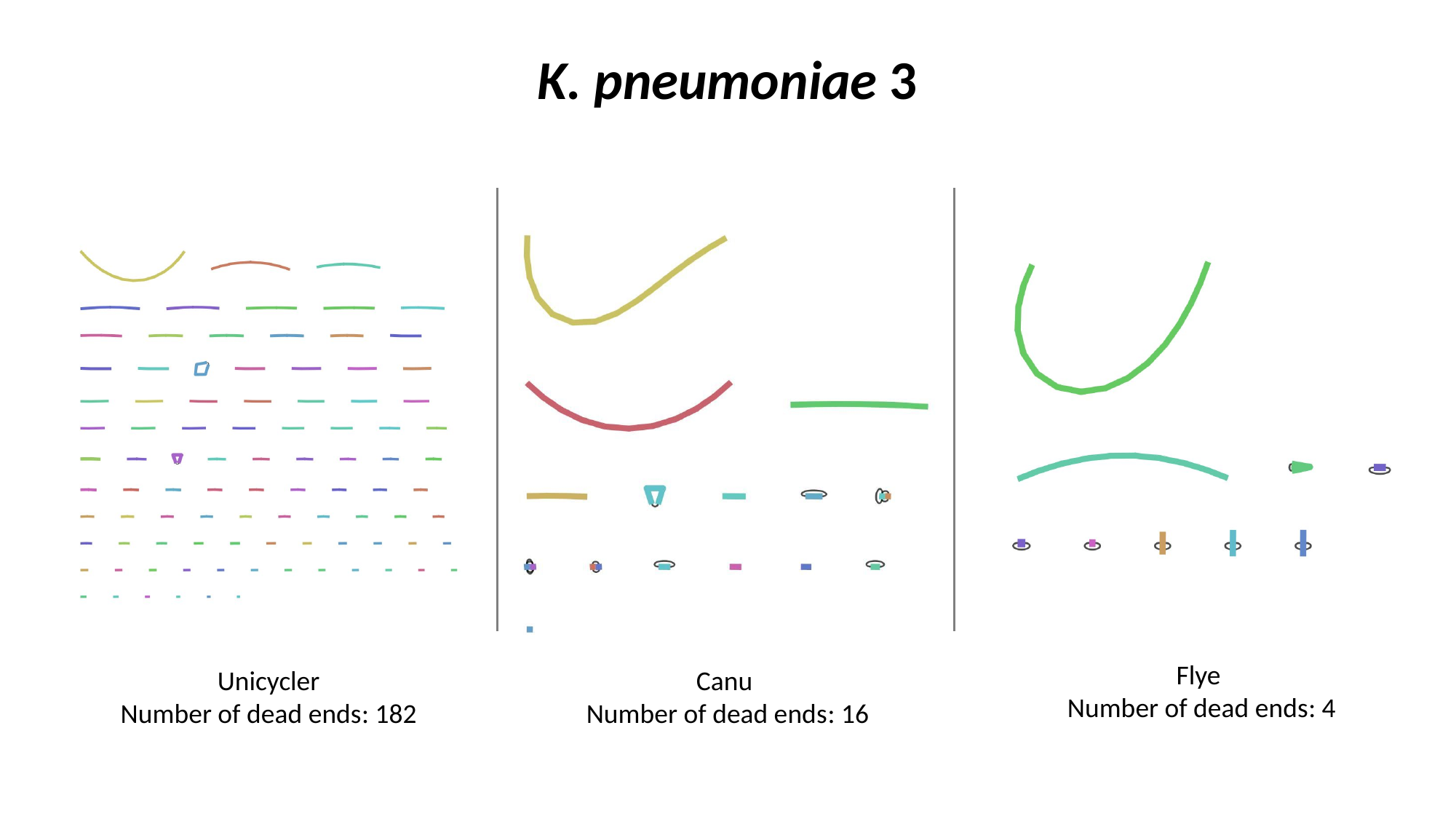

K. pneumoniae 3
Flye
Number of dead ends: 4
Unicycler
Number of dead ends: 182
Canu
Number of dead ends: 16

## Slide 9
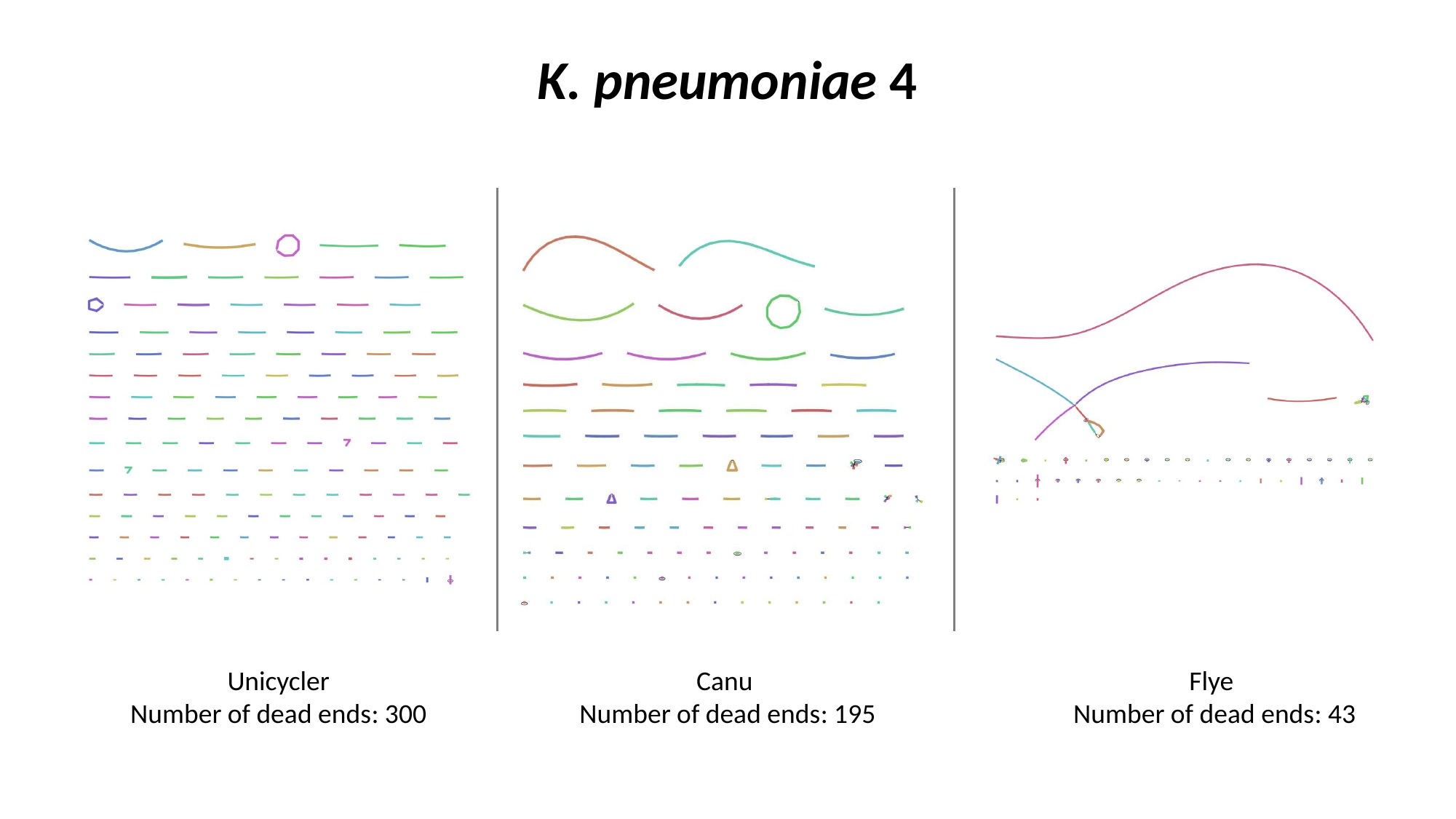

K. pneumoniae 4
Flye
Number of dead ends: 43
Unicycler
Number of dead ends: 300
Canu
Number of dead ends: 195

## Slide 10
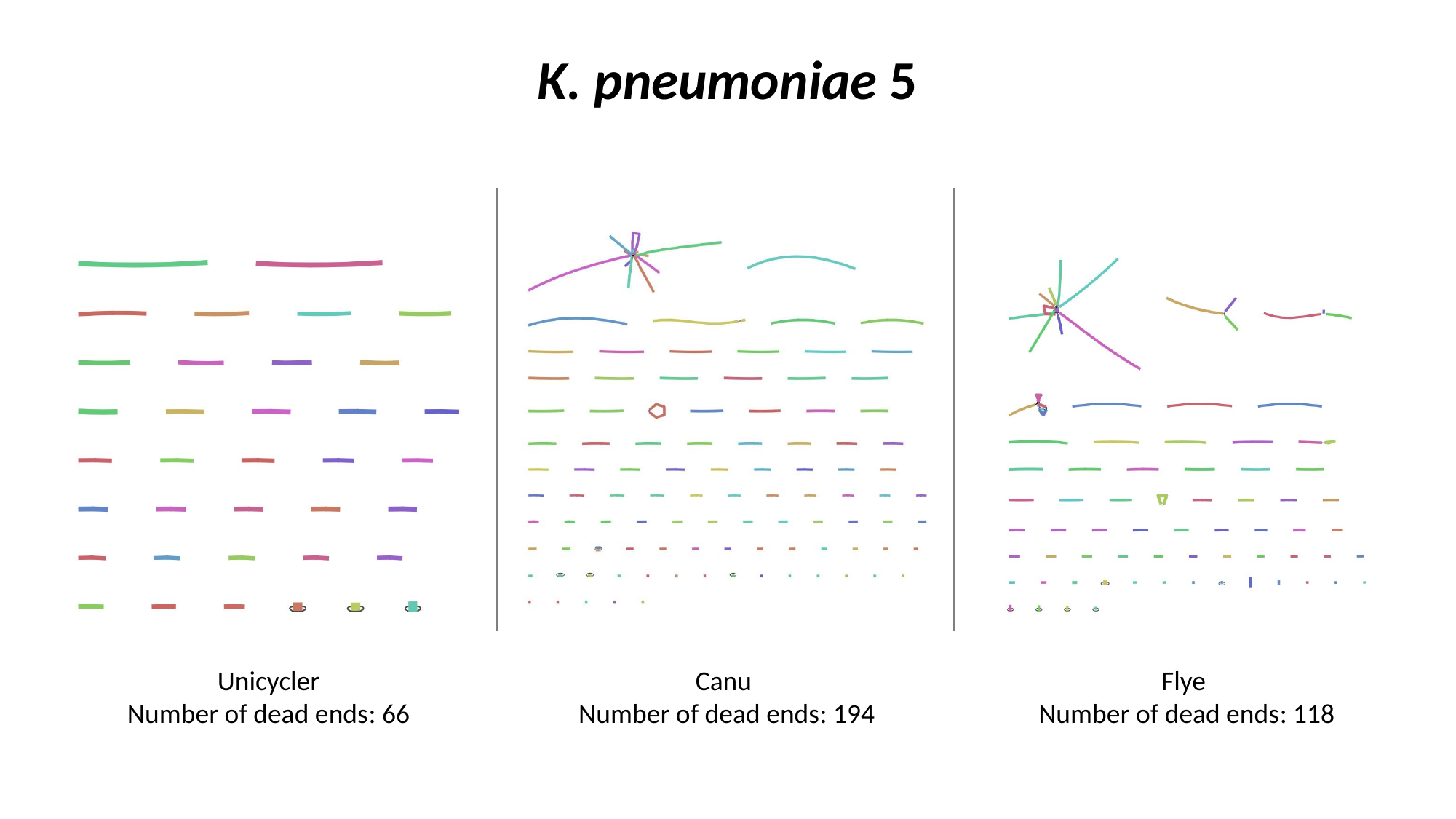

K. pneumoniae 5
Flye
Number of dead ends: 118
Unicycler
Number of dead ends: 66
Canu
Number of dead ends: 194

## Slide 11
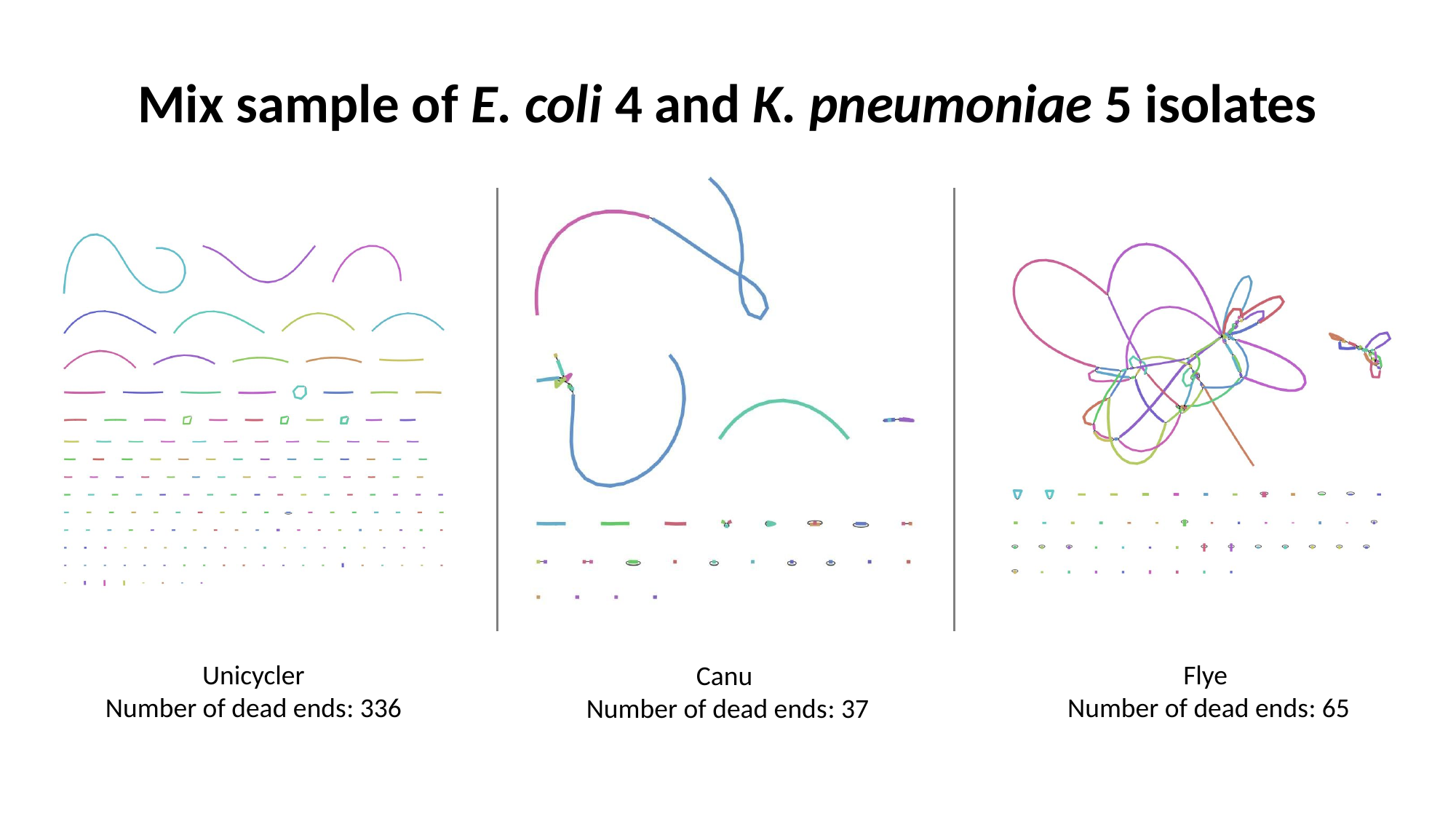

Mix sample of E. coli 4 and K. pneumoniae 5 isolates
Unicycler
Number of dead ends: 336
Flye
Number of dead ends: 65
Canu
Number of dead ends: 37
