# Supplementary material for: Hybrid Assembly Provides Improved Resolution of Plasmids, Antimicrobial Resistance Genes, and Virulence Factors in Escherichia coli and Klebsiella pneumoniae Clinical Isolates
Source: Microorganisms. 2021 Dec 10;9(12):2560. doi: 10.3390/microorganisms9122560 (PMC8704702; doi:10.3390/microorganisms9122560)
Supplement: Supplementary file 1 [file microorganisms-09-02560-s001.zip › Supplementary Figure S3_Assembly graphs for HybASM.PPTX]

## Slide 1
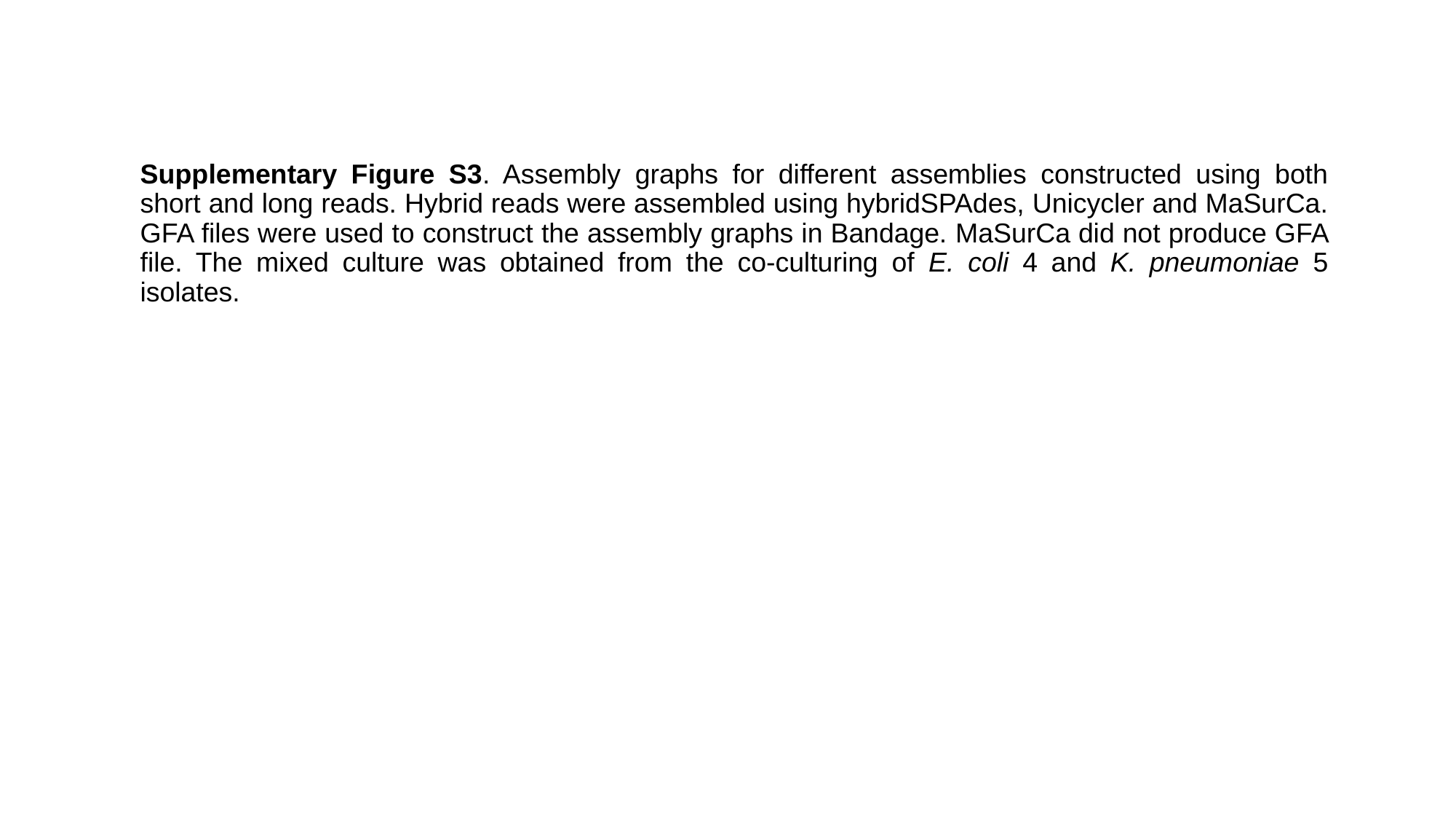

Supplementary Figure S3. Assembly graphs for different assemblies constructed using both short and long reads. Hybrid reads were assembled using hybridSPAdes, Unicycler and MaSurCa. GFA files were used to construct the assembly graphs in Bandage. MaSurCa did not produce GFA file. The mixed culture was obtained from the co-culturing of E. coli 4 and K. pneumoniae 5 isolates.

## Slide 2
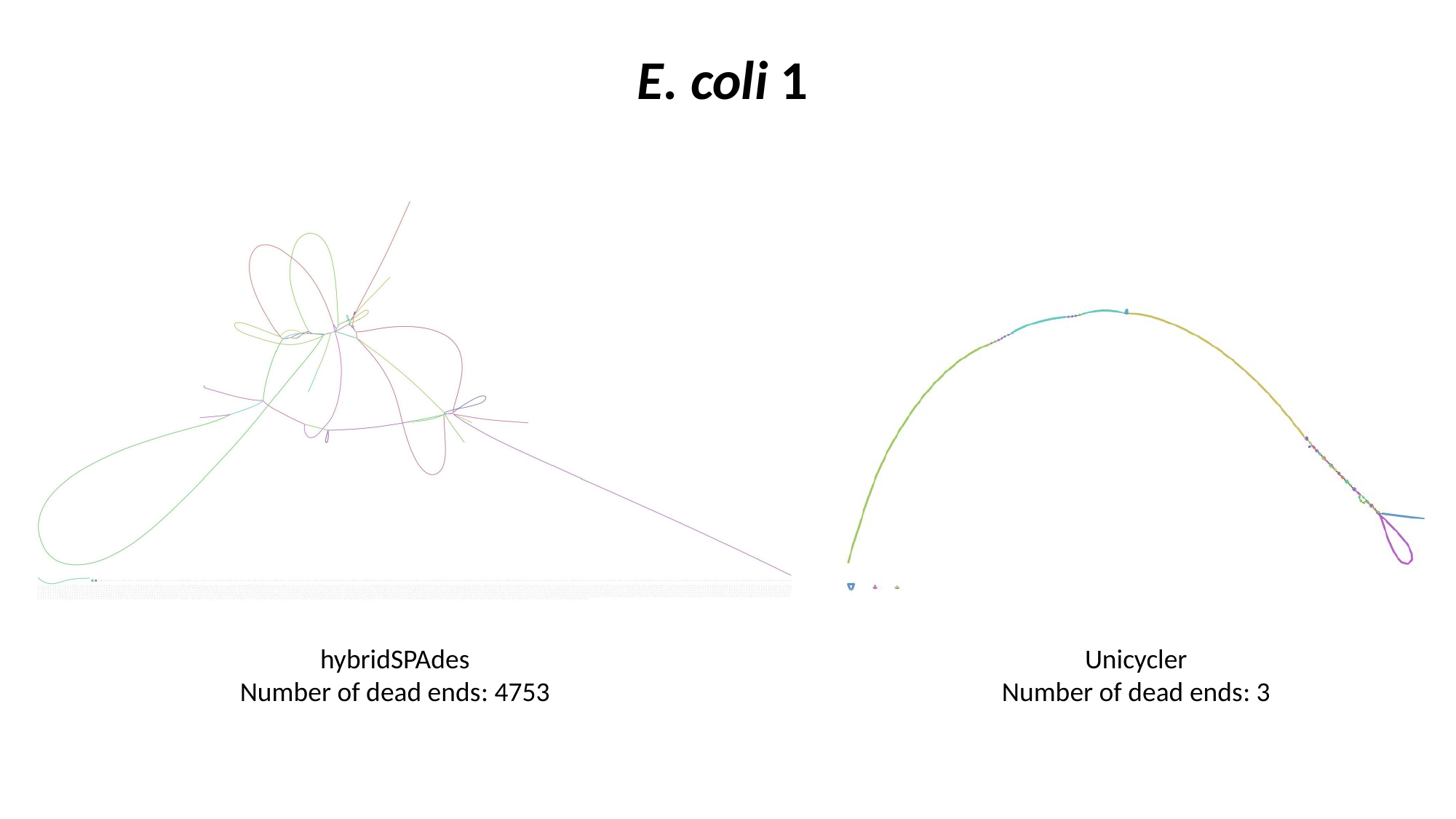

E. coli 1
Unicycler
Number of dead ends: 3
hybridSPAdes
Number of dead ends: 4753

## Slide 3
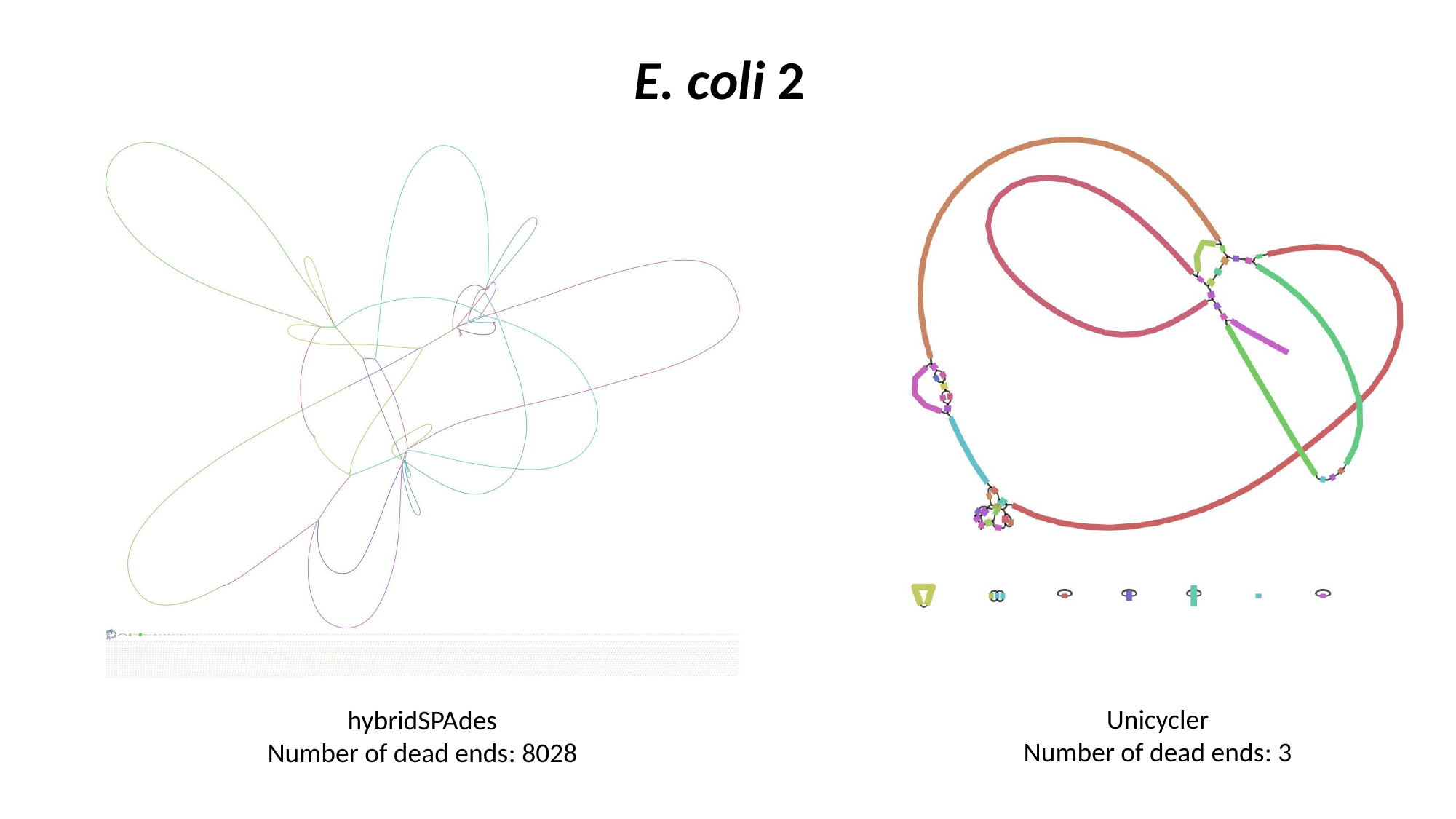

E. coli 2
Unicycler
Number of dead ends: 3
hybridSPAdes
Number of dead ends: 8028

## Slide 4
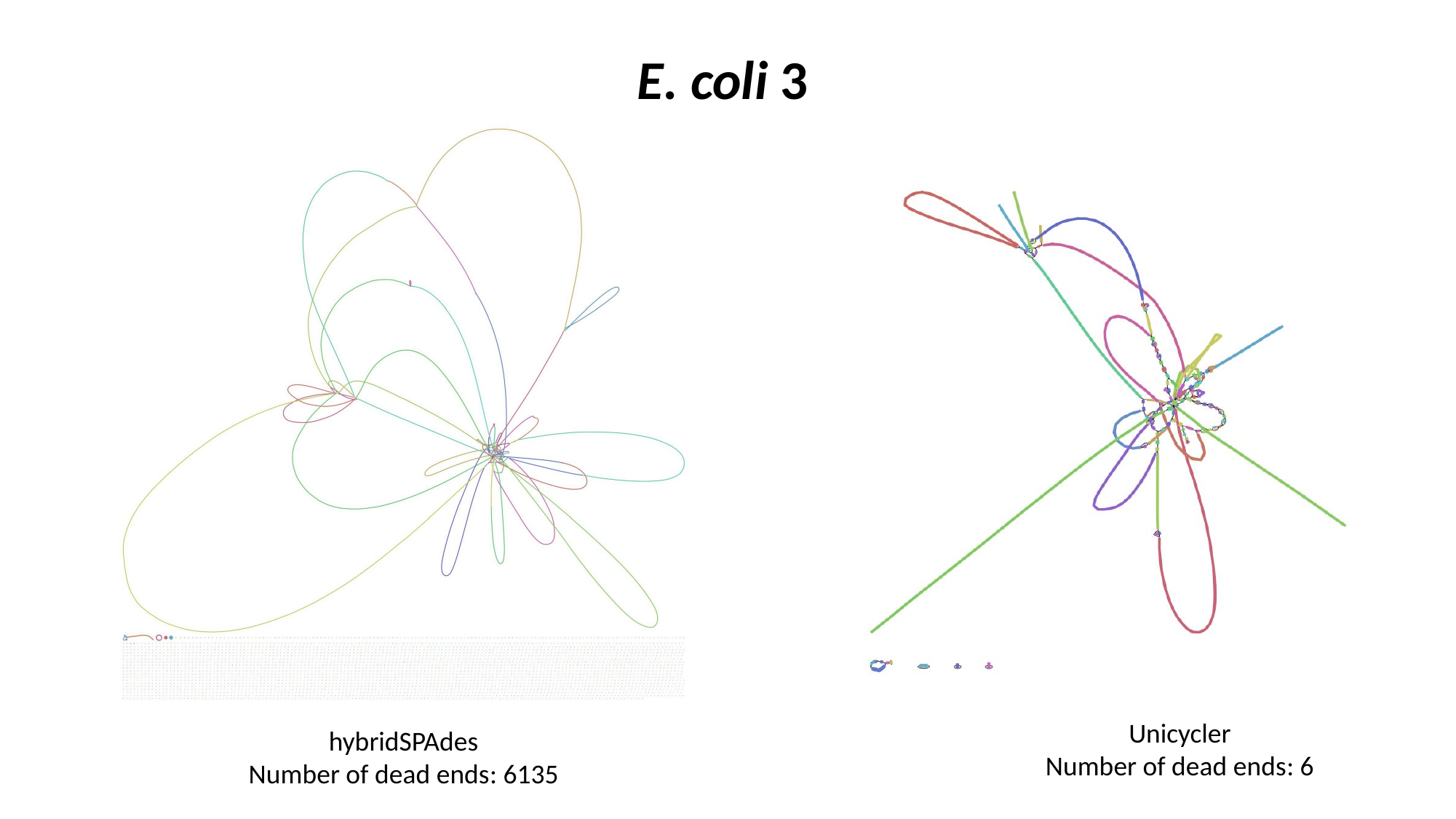

E. coli 3
Unicycler
Number of dead ends: 6
hybridSPAdes
Number of dead ends: 6135

## Slide 5
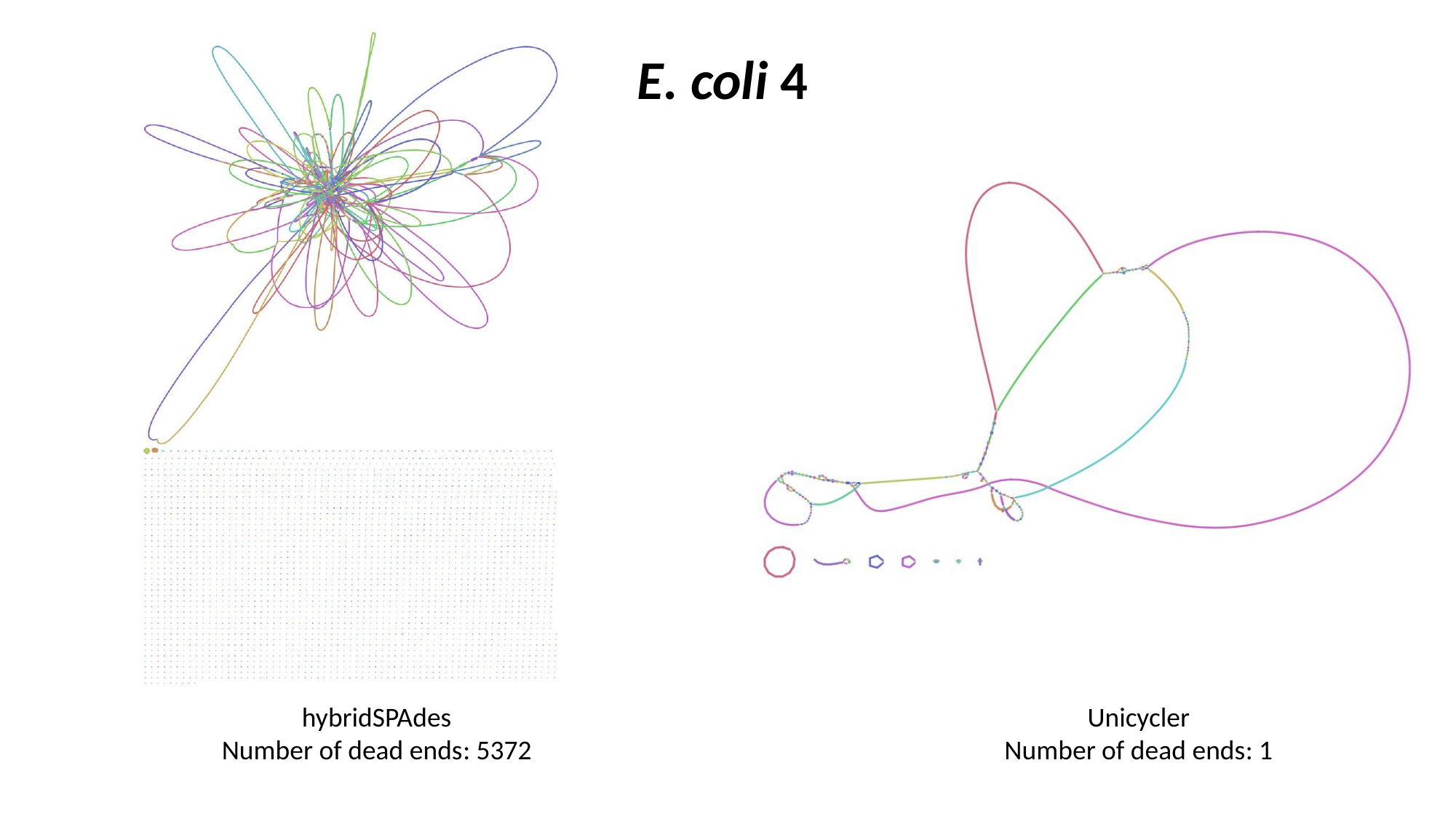

E. coli 4
hybridSPAdes
Number of dead ends: 5372
Unicycler
Number of dead ends: 1

## Slide 6
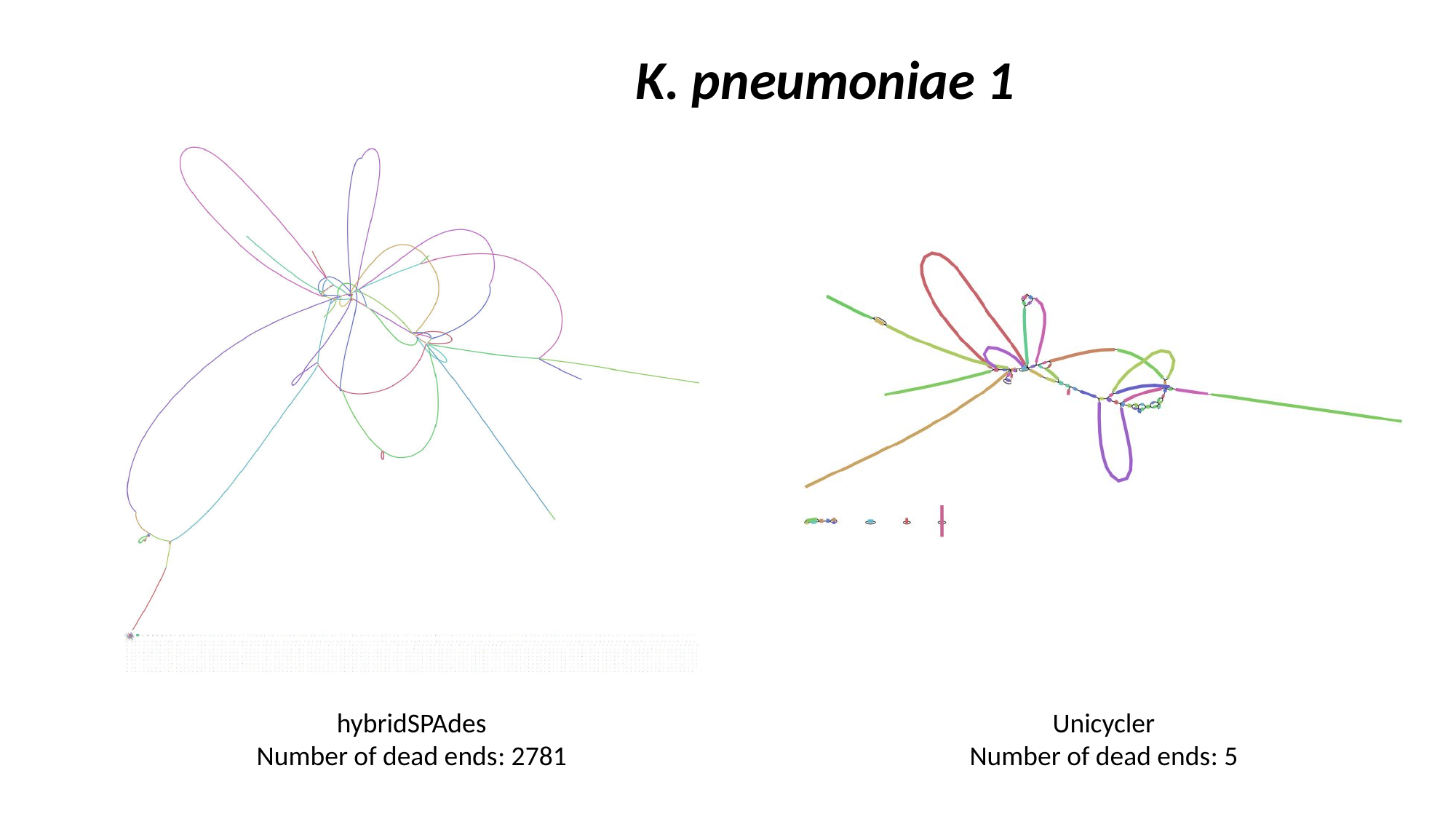

K. pneumoniae 1
Unicycler
Number of dead ends: 5
hybridSPAdes
Number of dead ends: 2781

## Slide 7
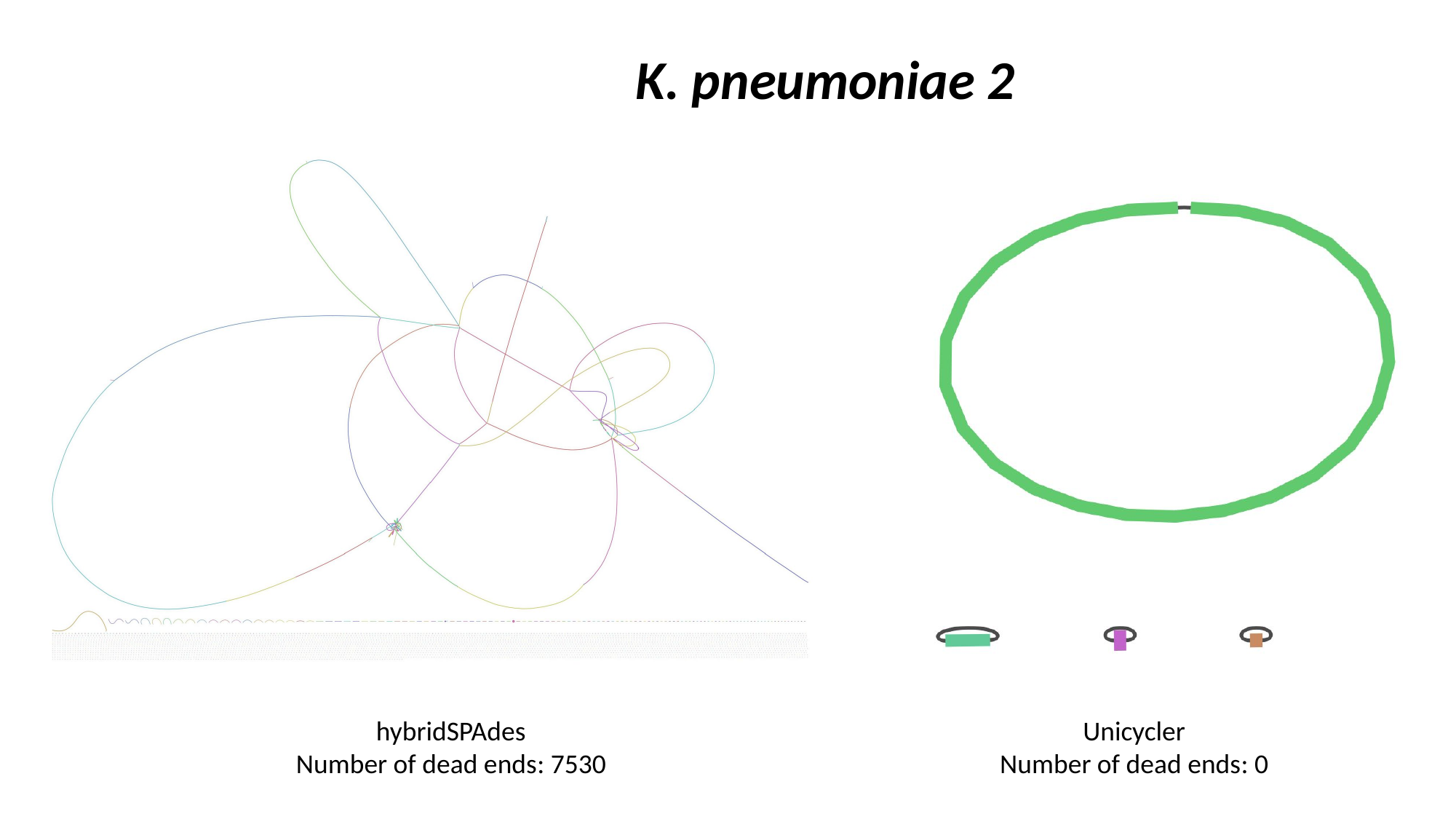

K. pneumoniae 2
hybridSPAdes
Number of dead ends: 7530
Unicycler
Number of dead ends: 0

## Slide 8
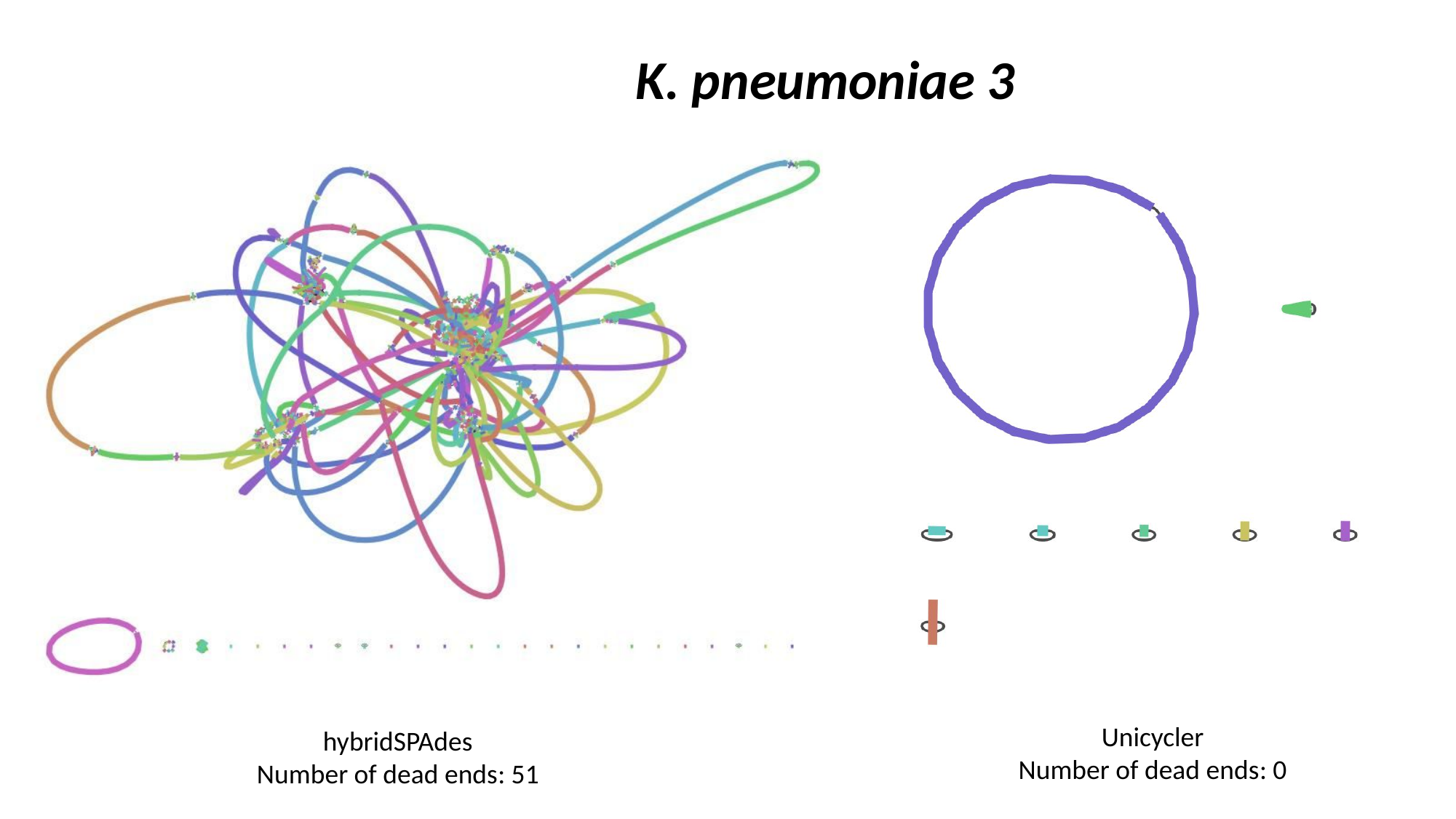

K. pneumoniae 3
Unicycler
Number of dead ends: 0
hybridSPAdes
Number of dead ends: 51

## Slide 9
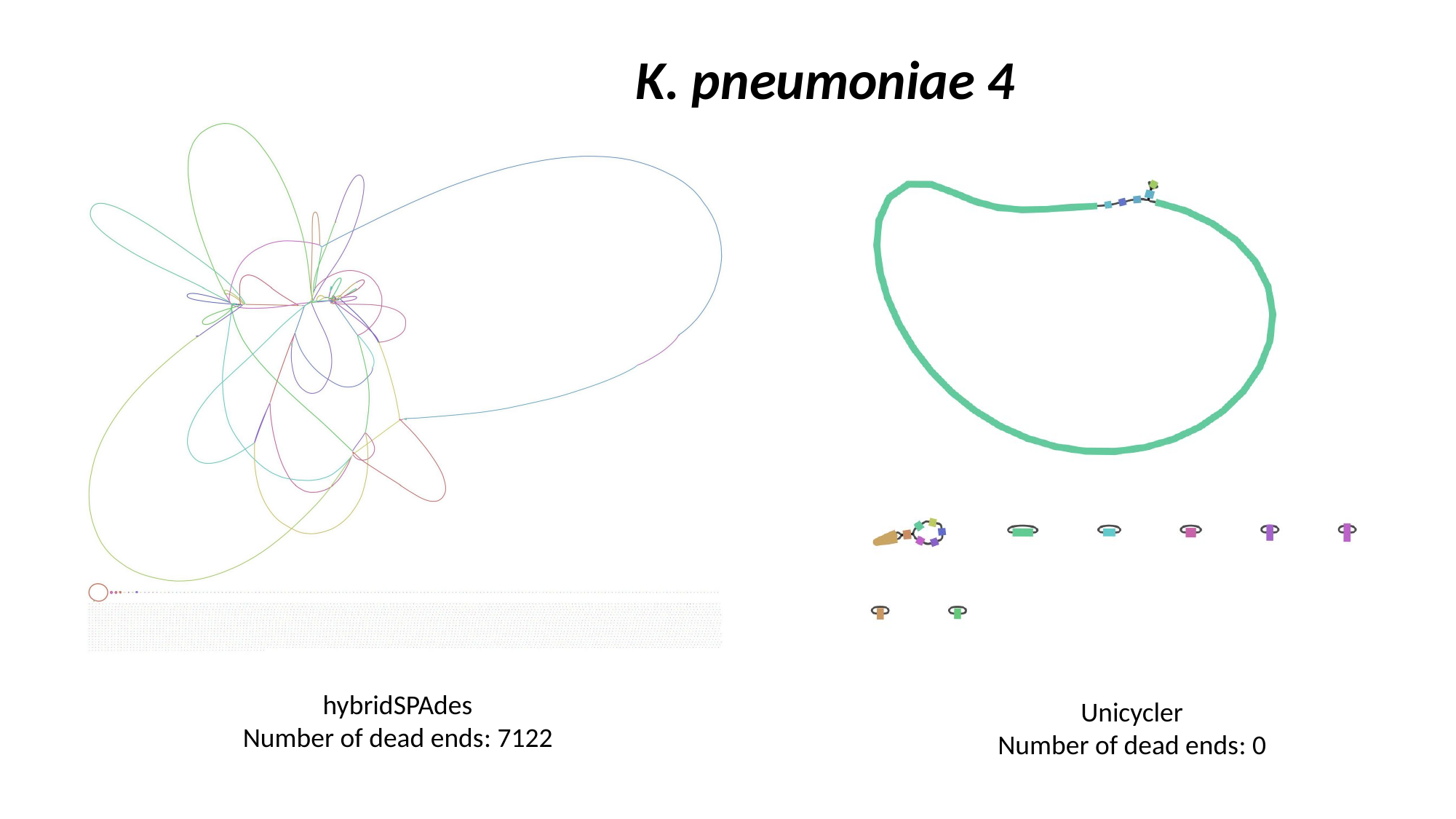

K. pneumoniae 4
hybridSPAdes
Number of dead ends: 7122
Unicycler
Number of dead ends: 0

## Slide 10
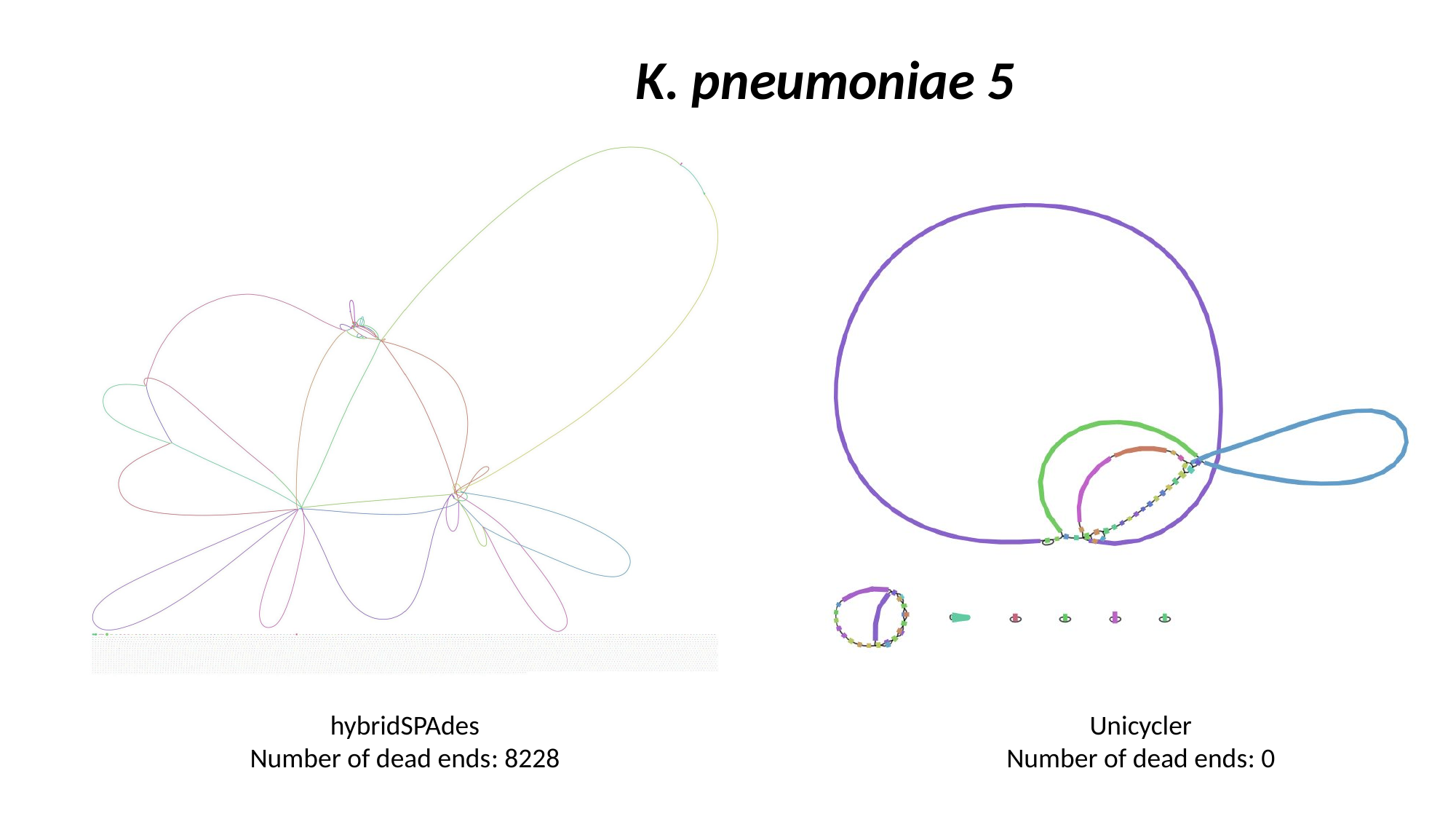

K. pneumoniae 5
hybridSPAdes
Number of dead ends: 8228
Unicycler
Number of dead ends: 0

## Slide 11
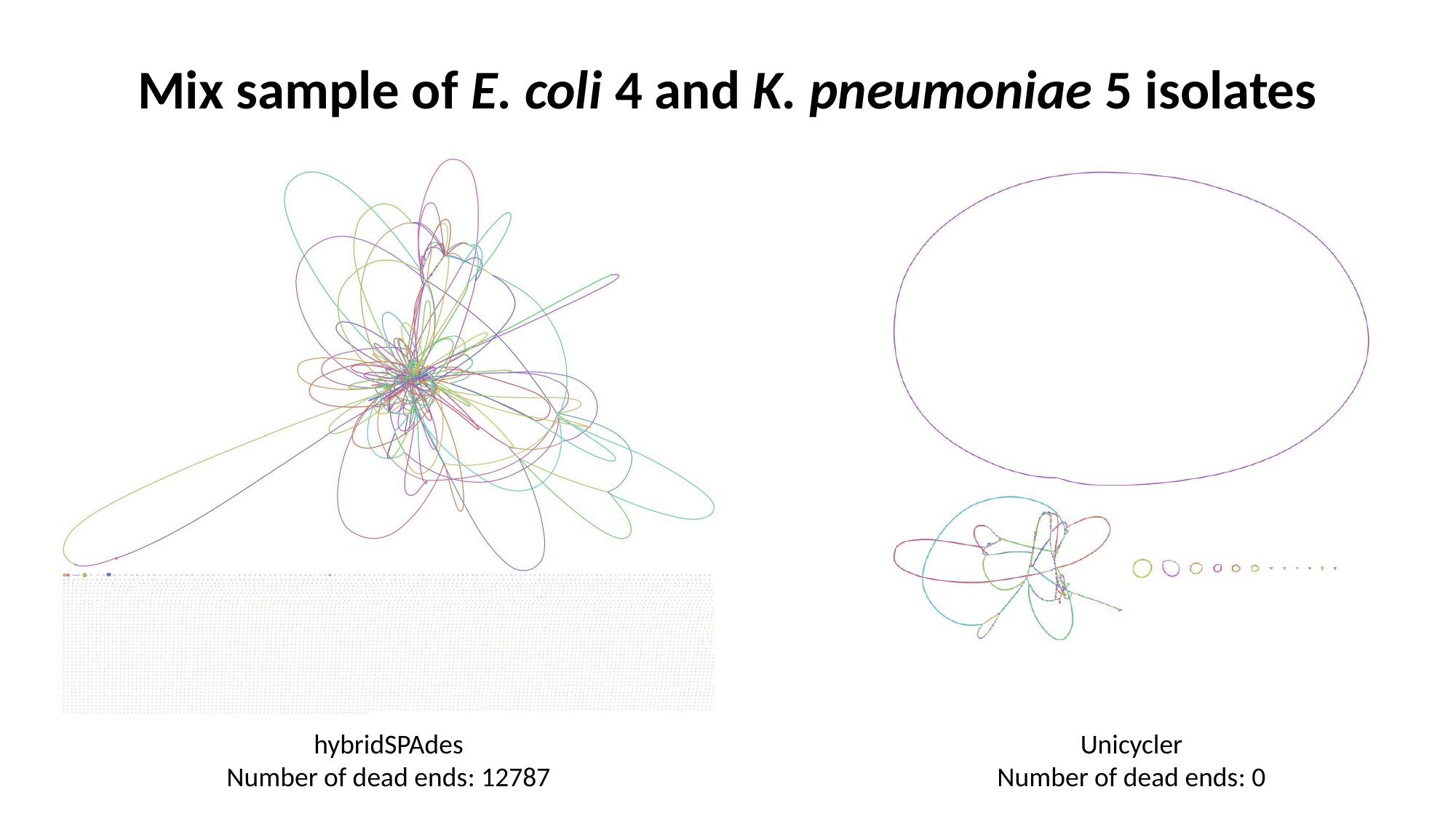

Mix sample of E. coli 4 and K. pneumoniae 5 isolates
Unicycler
Number of dead ends: 0
hybridSPAdes
Number of dead ends: 12787
