# Supplementary material for: Hybrid Assembly Provides Improved Resolution of Plasmids, Antimicrobial Resistance Genes, and Virulence Factors in Escherichia coli and Klebsiella pneumoniae Clinical Isolates
Source: Microorganisms. 2021 Dec 10;9(12):2560. doi: 10.3390/microorganisms9122560 (PMC8704702; doi:10.3390/microorganisms9122560)
Supplement: Supplementary file 1 [file microorganisms-09-02560-s001.zip › Supplementary Figure S4_Assembly graphs for assemblies from top performed assemblers.PPTX]

## Slide 1
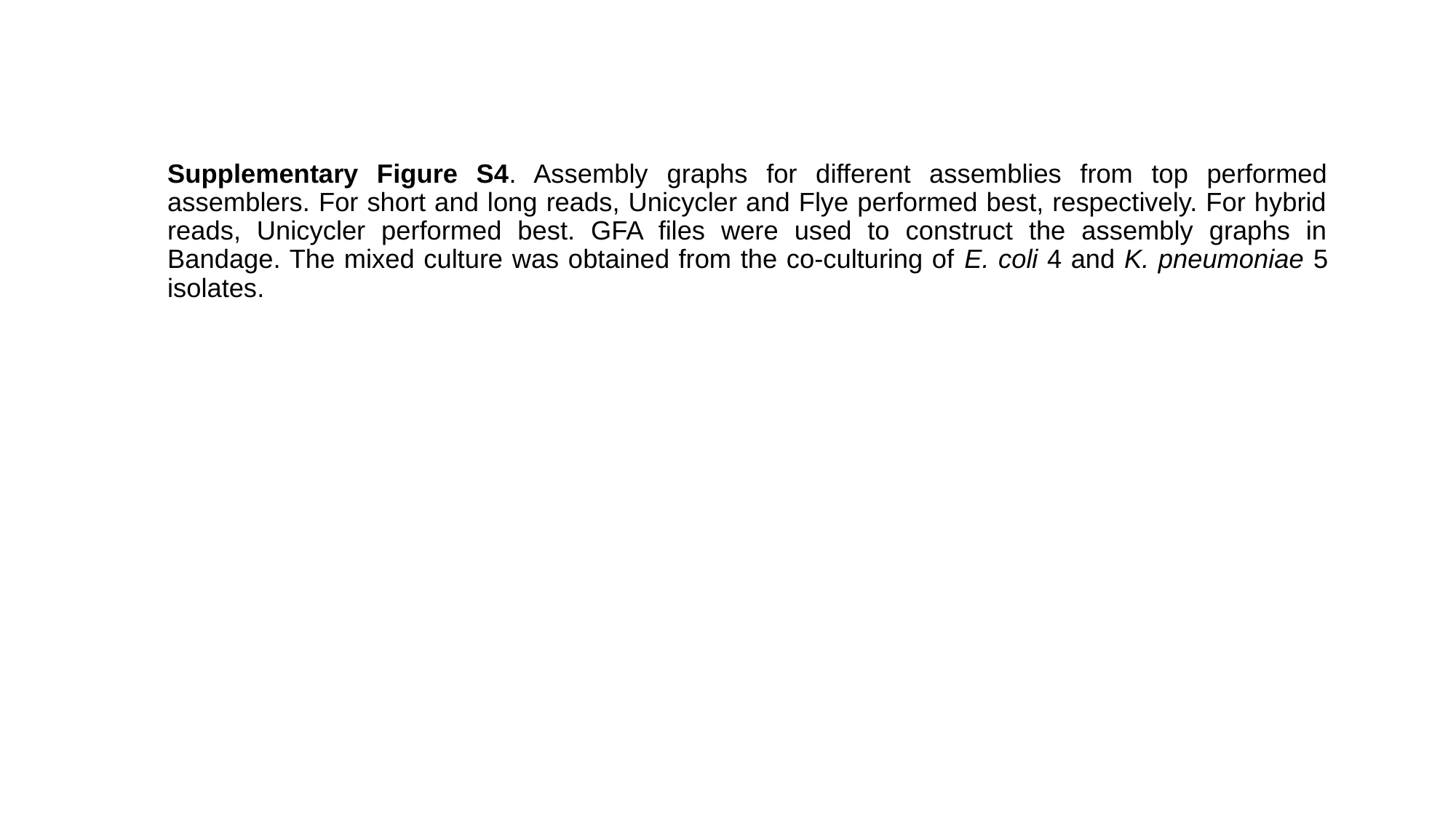

Supplementary Figure S4. Assembly graphs for different assemblies from top performed assemblers. For short and long reads, Unicycler and Flye performed best, respectively. For hybrid reads, Unicycler performed best. GFA files were used to construct the assembly graphs in Bandage. The mixed culture was obtained from the co-culturing of E. coli 4 and K. pneumoniae 5 isolates.

## Slide 2
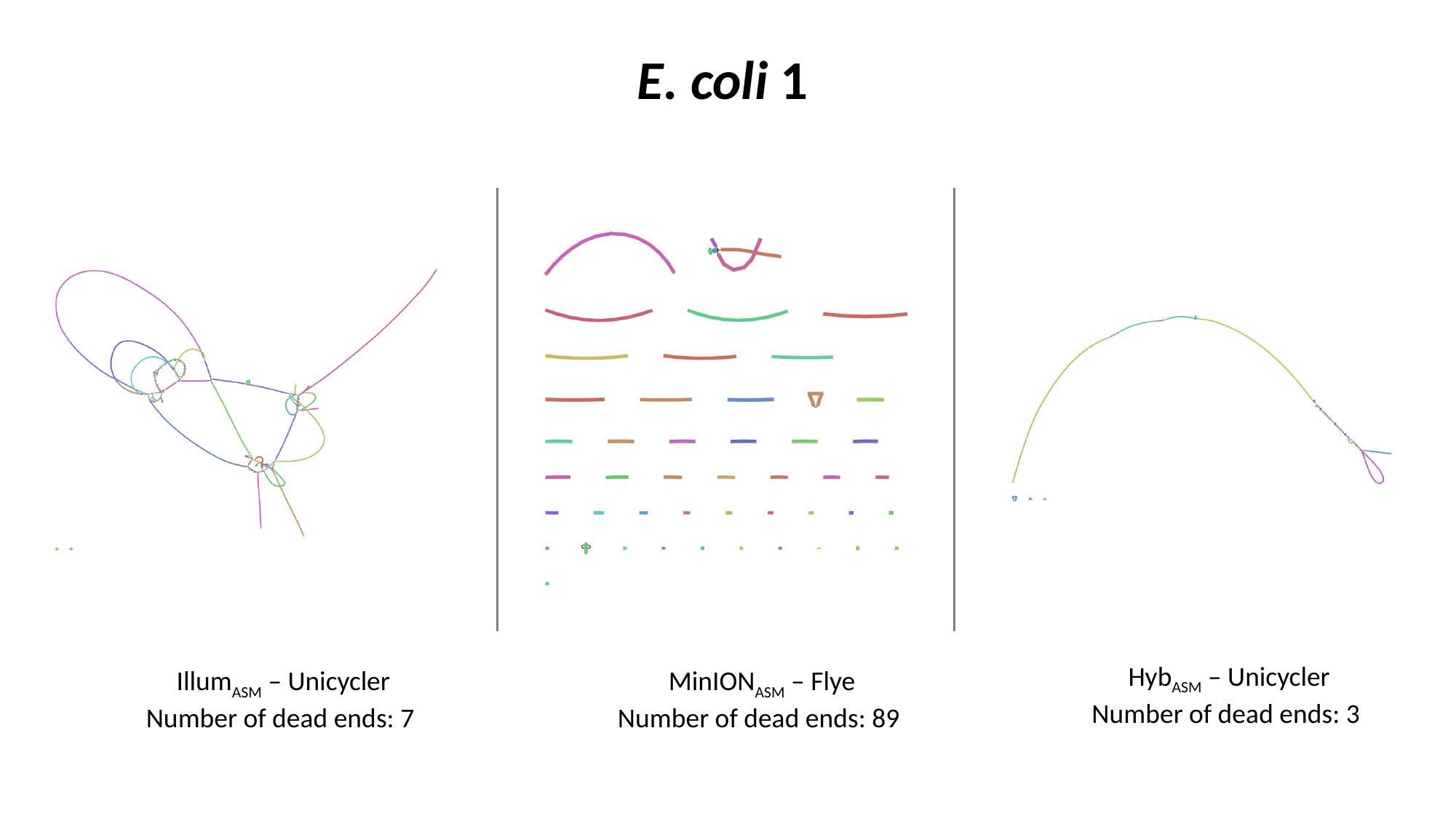

E. coli 1
HybASM – Unicycler
Number of dead ends: 3
IllumASM – Unicycler
Number of dead ends: 7
MinIONASM – Flye
Number of dead ends: 89

## Slide 3
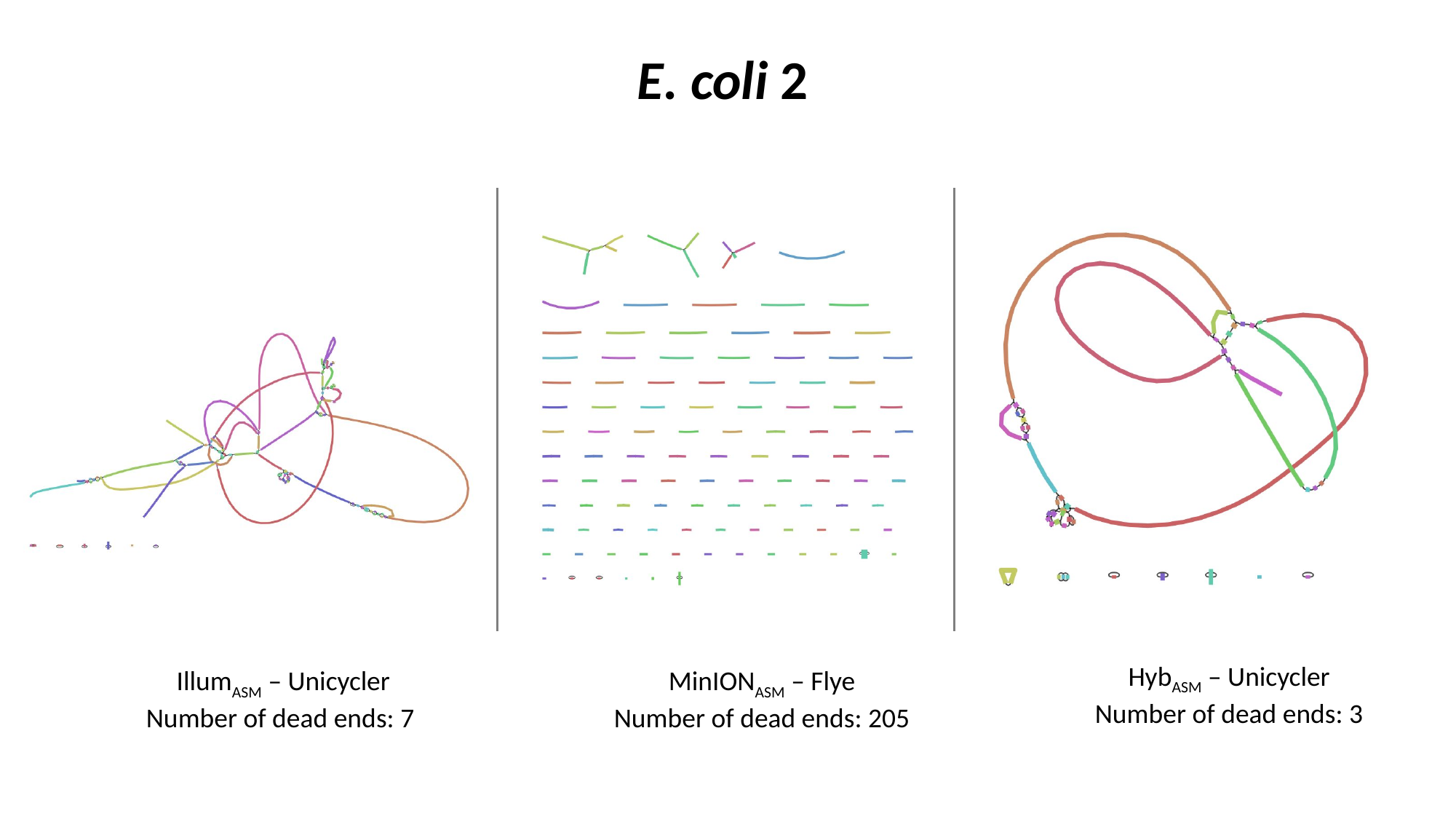

E. coli 2
HybASM – Unicycler
Number of dead ends: 3
IllumASM – Unicycler
Number of dead ends: 7
MinIONASM – Flye
Number of dead ends: 205

## Slide 4
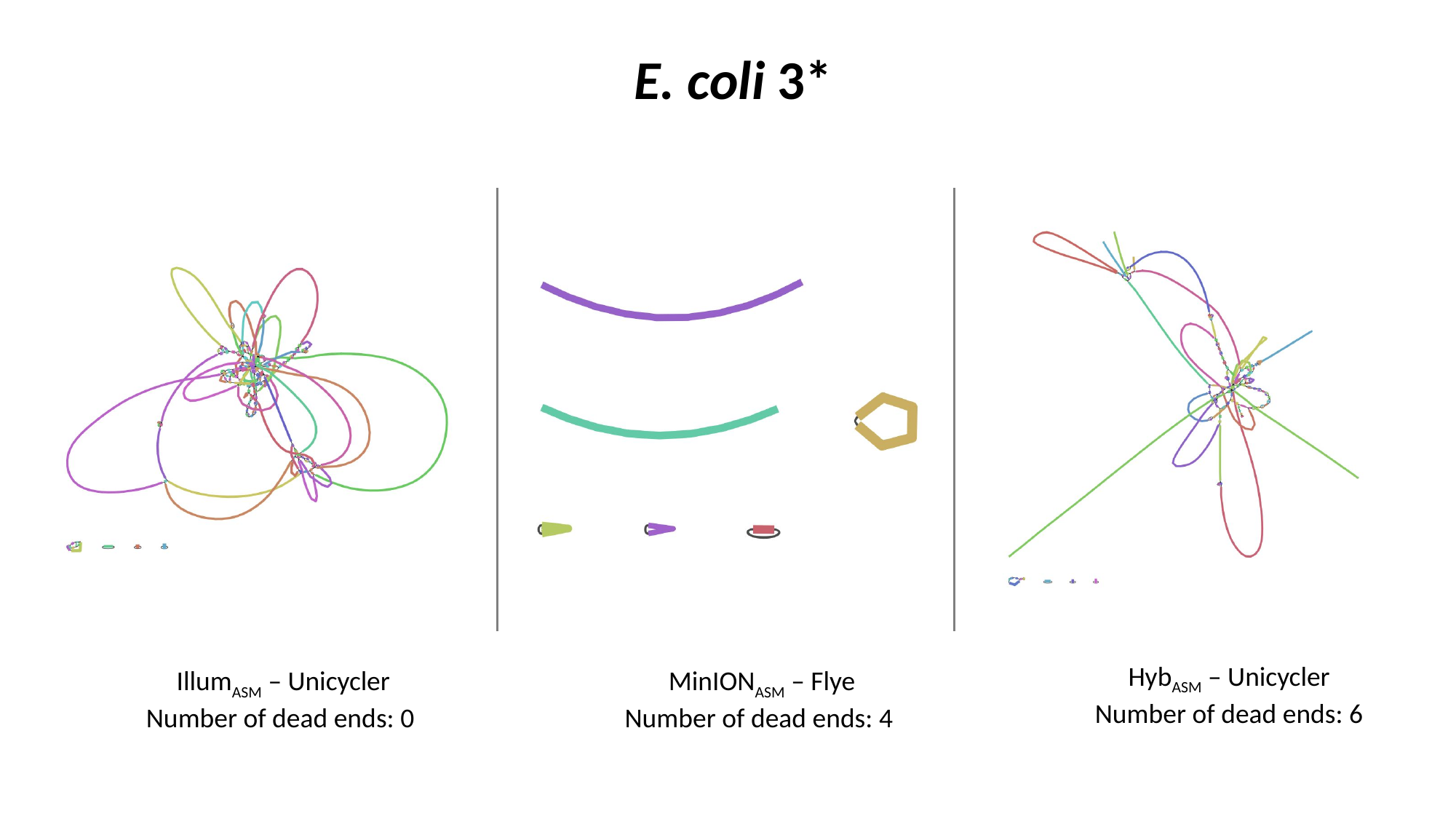

E. coli 3*
HybASM – Unicycler
Number of dead ends: 6
IllumASM – Unicycler
Number of dead ends: 0
MinIONASM – Flye
Number of dead ends: 4

## Slide 5
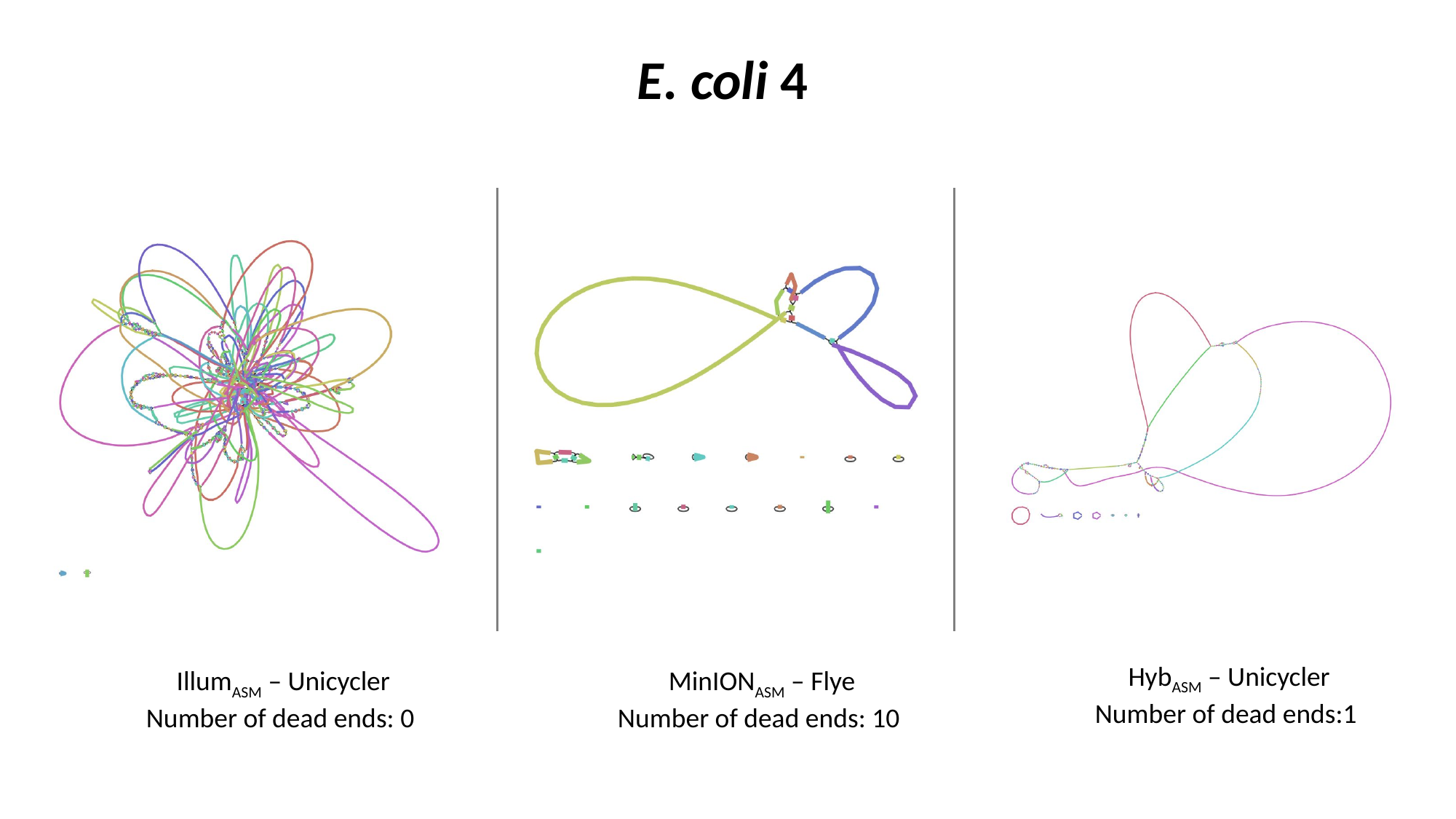

E. coli 4
HybASM – Unicycler
Number of dead ends:1
IllumASM – Unicycler
Number of dead ends: 0
MinIONASM – Flye
Number of dead ends: 10

## Slide 6
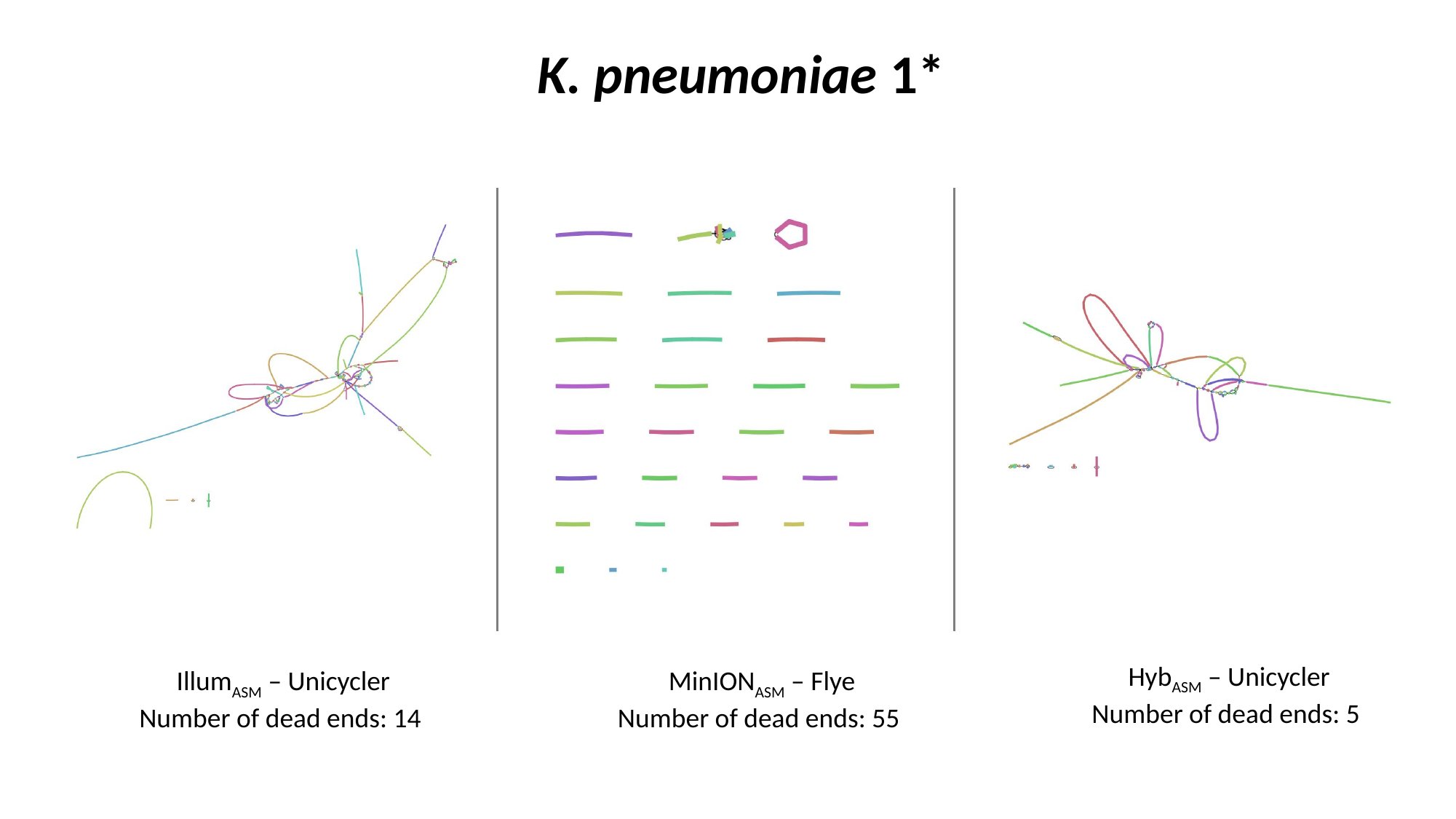

K. pneumoniae 1*
HybASM – Unicycler
Number of dead ends: 5
IllumASM – Unicycler
Number of dead ends: 14
MinIONASM – Flye
Number of dead ends: 55

## Slide 7
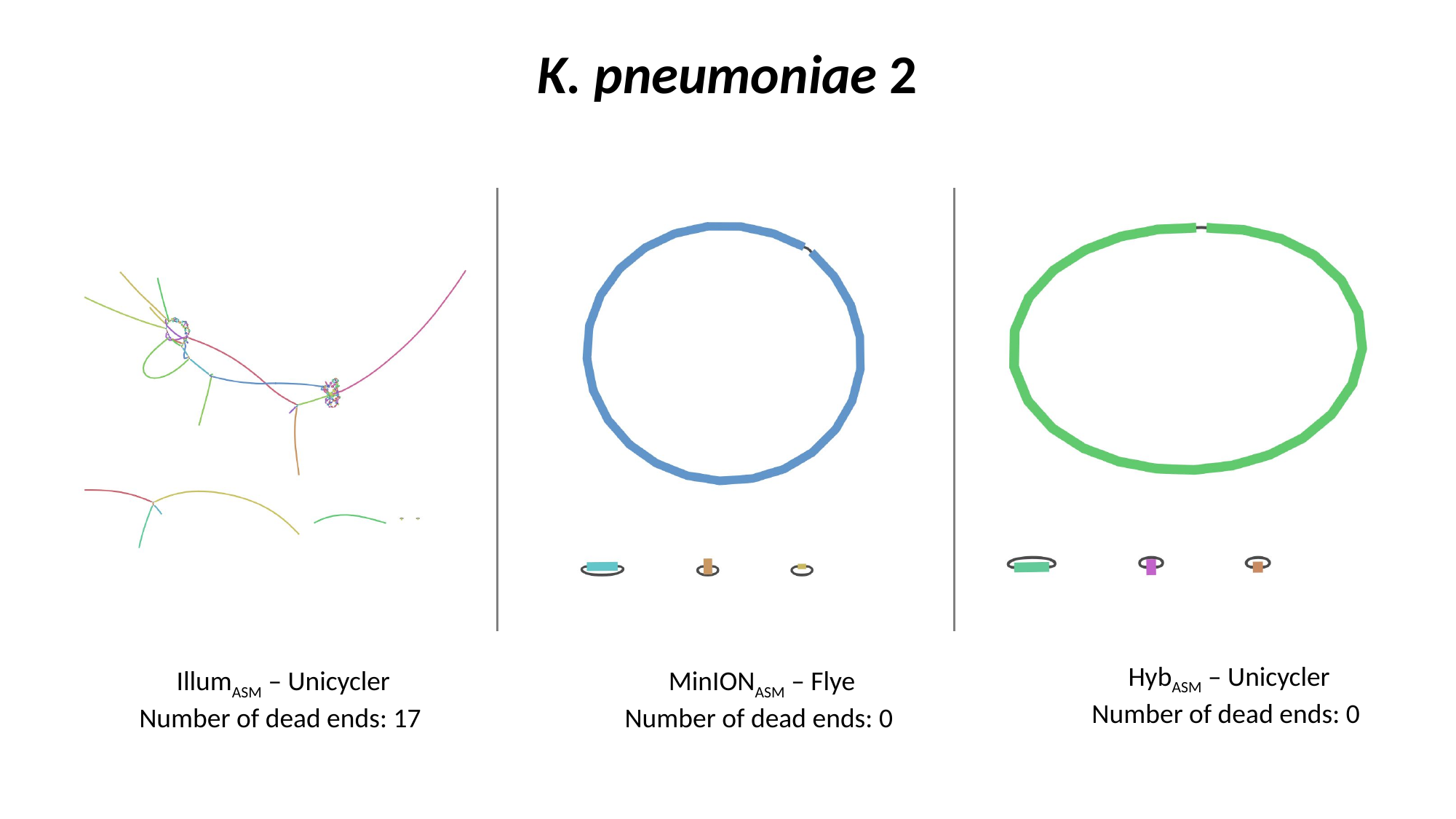

K. pneumoniae 2
HybASM – Unicycler
Number of dead ends: 0
IllumASM – Unicycler
Number of dead ends: 17
MinIONASM – Flye
Number of dead ends: 0

## Slide 8
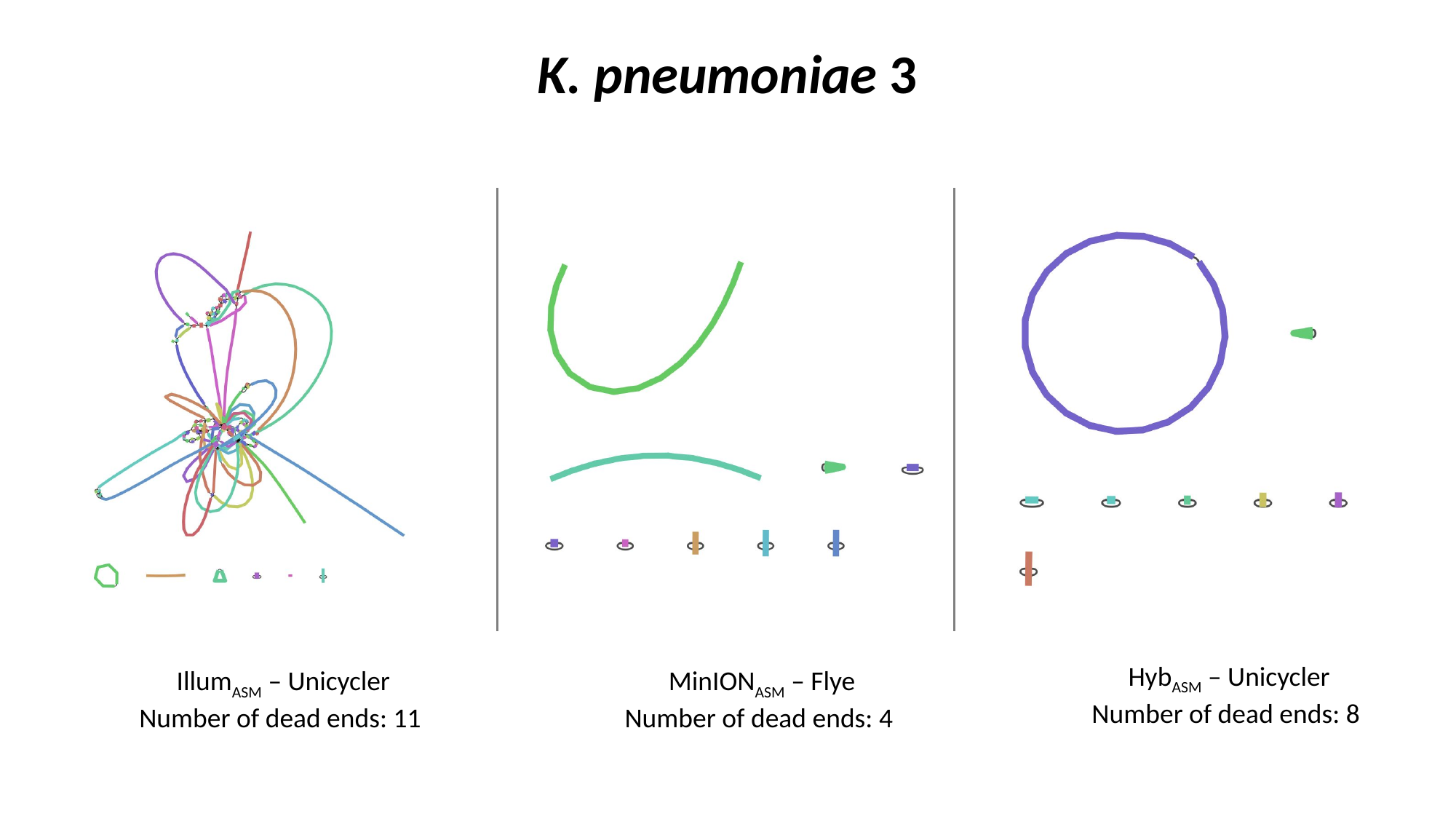

K. pneumoniae 3
HybASM – Unicycler
Number of dead ends: 8
IllumASM – Unicycler
Number of dead ends: 11
MinIONASM – Flye
Number of dead ends: 4

## Slide 9
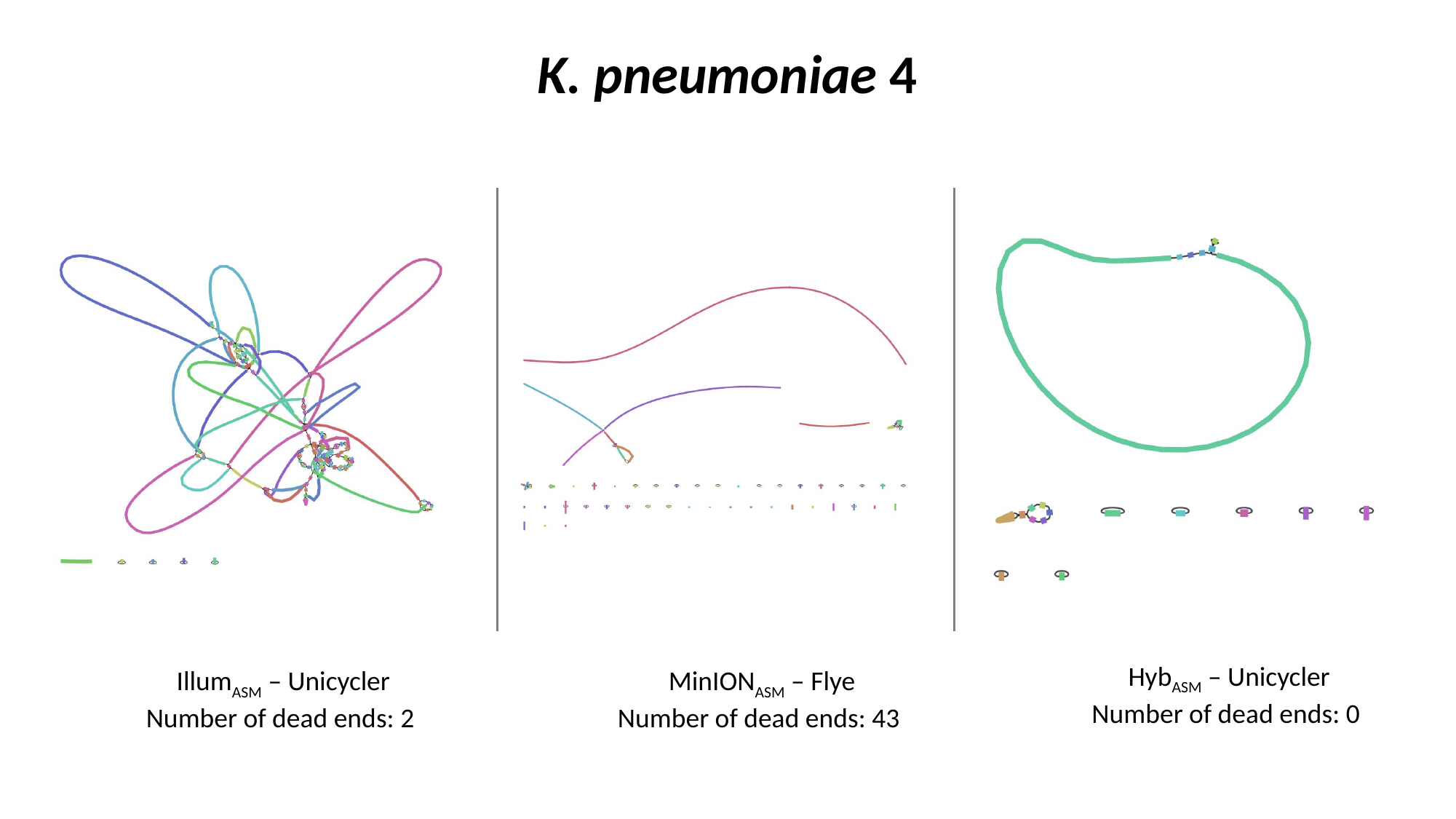

K. pneumoniae 4
HybASM – Unicycler
Number of dead ends: 0
IllumASM – Unicycler
Number of dead ends: 2
MinIONASM – Flye
Number of dead ends: 43

## Slide 10
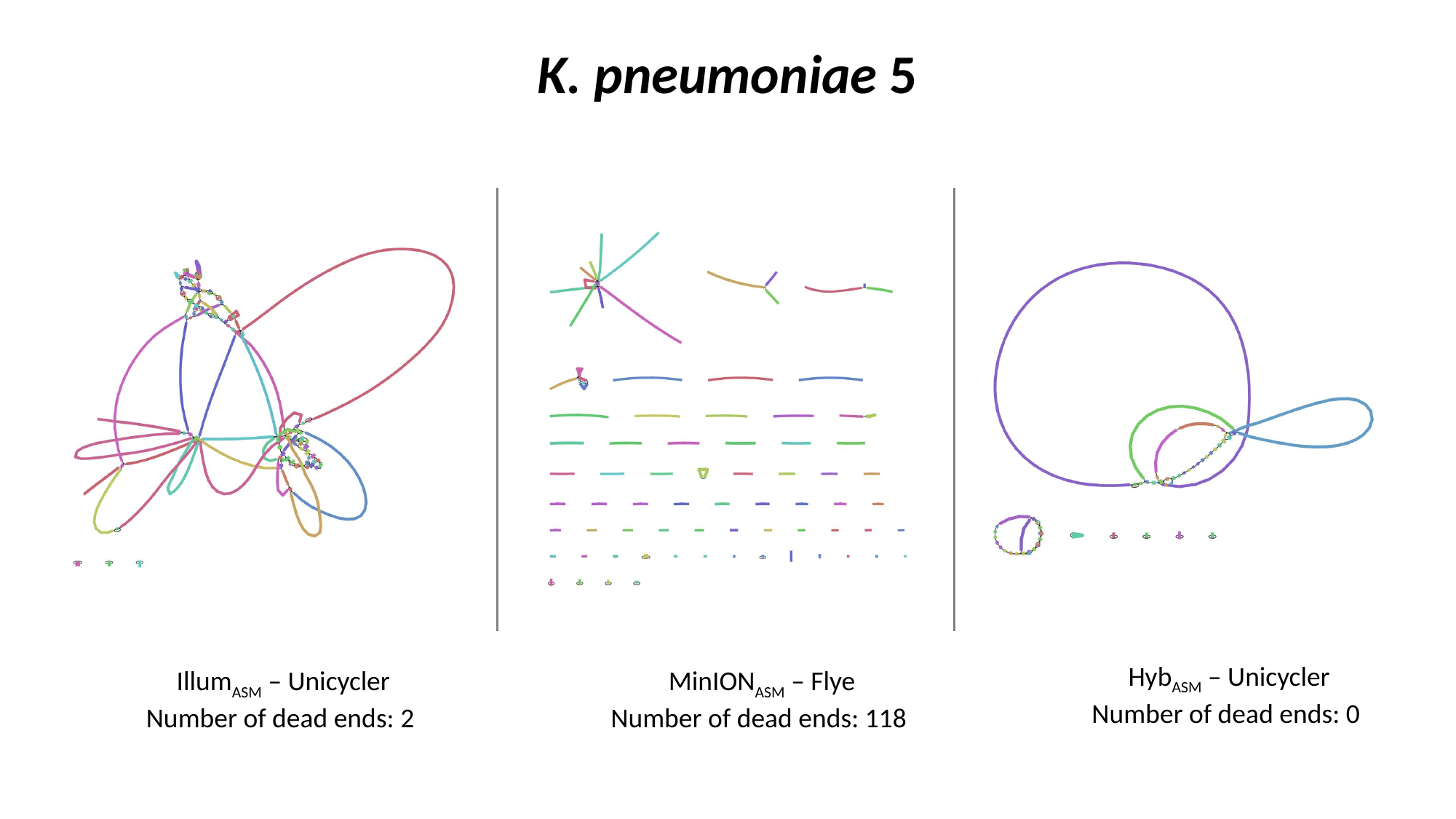

K. pneumoniae 5
HybASM – Unicycler
Number of dead ends: 0
IllumASM – Unicycler
Number of dead ends: 2
MinIONASM – Flye
Number of dead ends: 118

## Slide 11
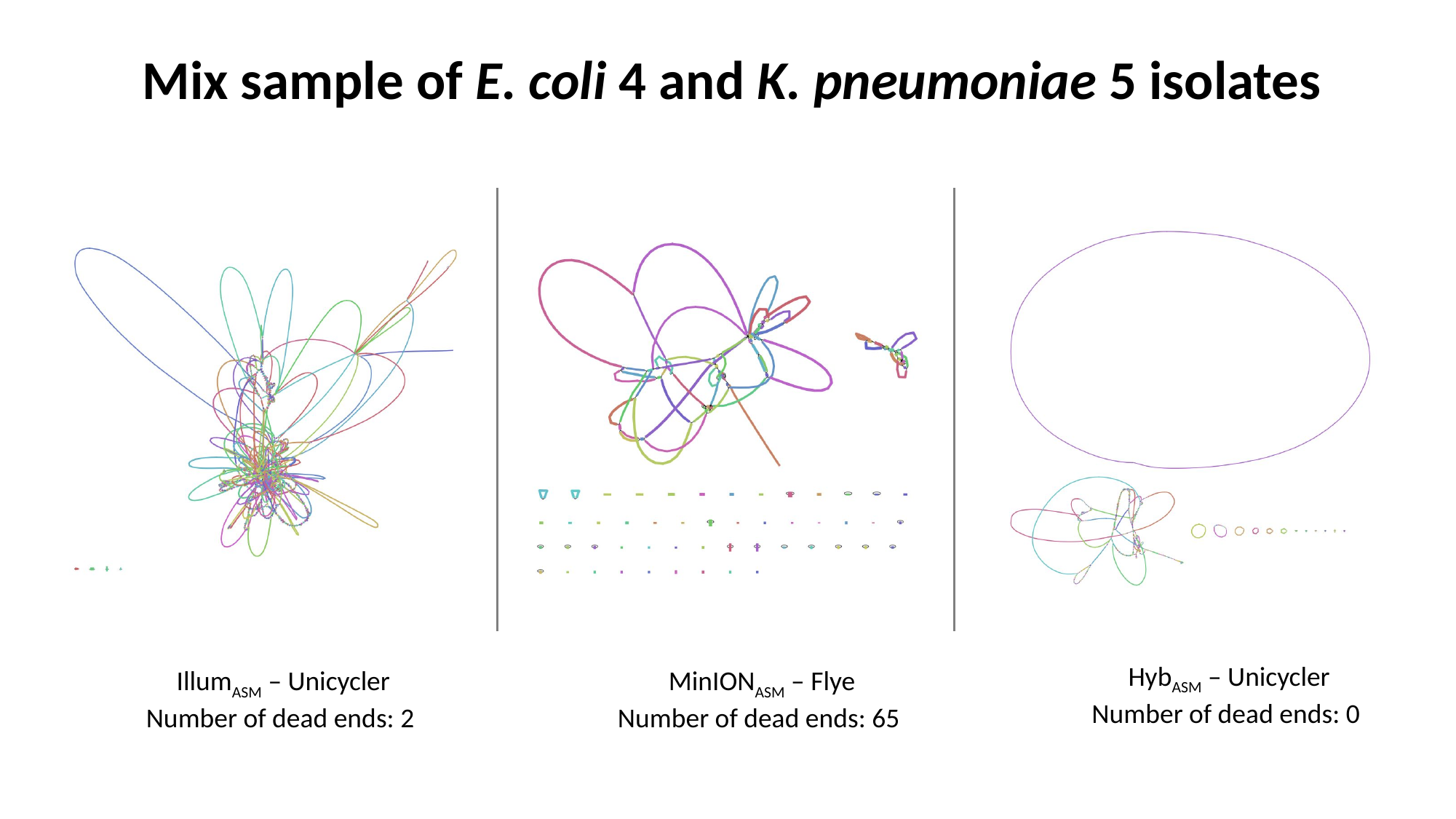

Mix sample of E. coli 4 and K. pneumoniae 5 isolates
HybASM – Unicycler
Number of dead ends: 0
IllumASM – Unicycler
Number of dead ends: 2
MinIONASM – Flye
Number of dead ends: 65
