# Supplementary material for: Hybrid Assembly Provides Improved Resolution of Plasmids, Antimicrobial Resistance Genes, and Virulence Factors in Escherichia coli and Klebsiella pneumoniae Clinical Isolates
Source: Microorganisms. 2021 Dec 10;9(12):2560. doi: 10.3390/microorganisms9122560 (PMC8704702; doi:10.3390/microorganisms9122560)
Supplement: Supplementary file 1 [file microorganisms-09-02560-s001.zip › Supplementary Figure S5_BUSCO graphs for all assemblies produced by different assemblers.pptx]

## Slide 1
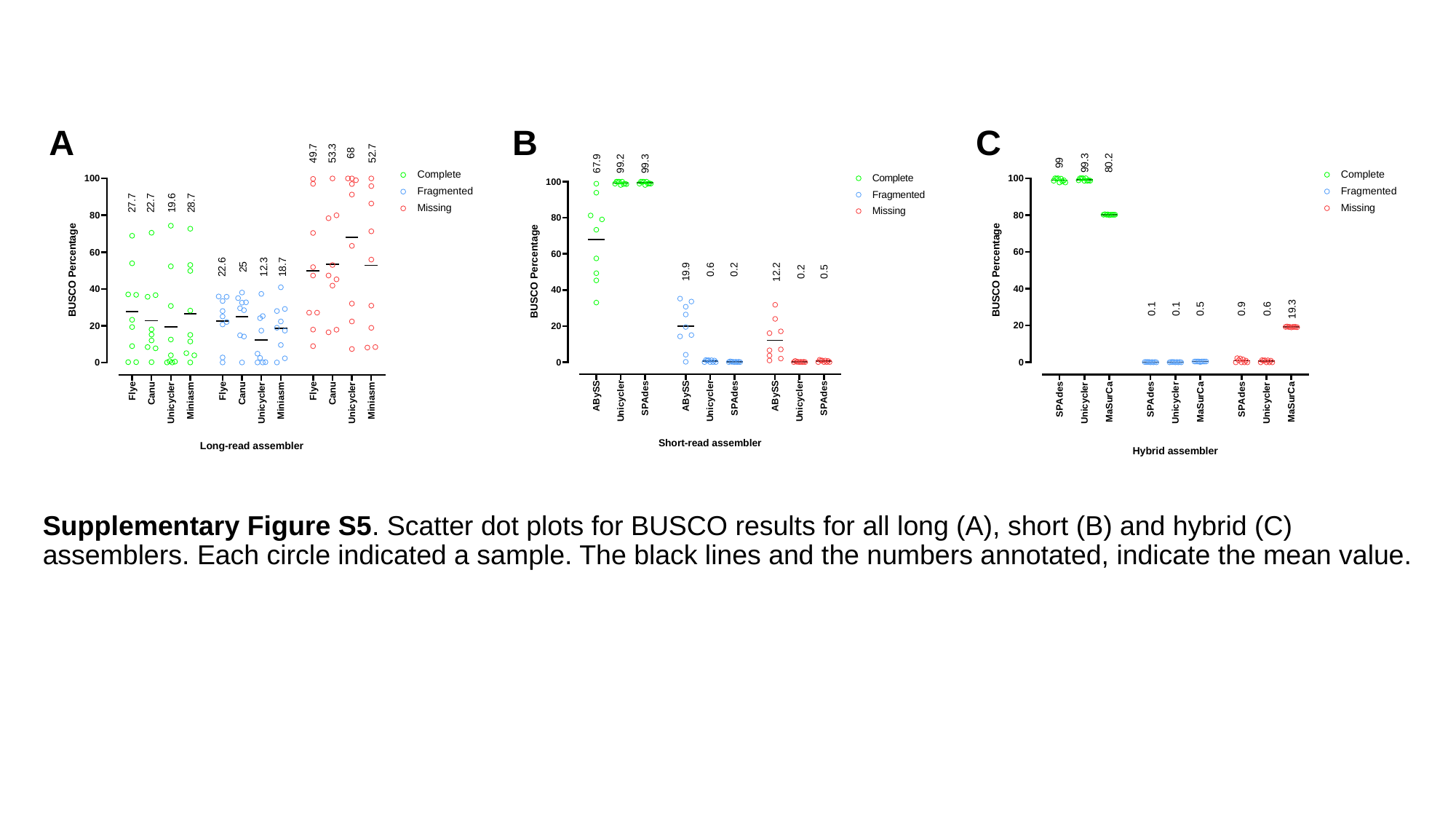

Supplementary Figure S5. Scatter dot plots for BUSCO results for all long (A), short (B) and hybrid (C) assemblers. Each circle indicated a sample. The black lines and the numbers annotated, indicate the mean value.
