# Supplementary material for: Hybrid Assembly Provides Improved Resolution of Plasmids, Antimicrobial Resistance Genes, and Virulence Factors in Escherichia coli and Klebsiella pneumoniae Clinical Isolates
Source: Microorganisms. 2021 Dec 10;9(12):2560. doi: 10.3390/microorganisms9122560 (PMC8704702; doi:10.3390/microorganisms9122560)
Supplement: Supplementary file 1 [file microorganisms-09-02560-s001.zip › Supplementary Table S4_ Assembly statestics for assemblies produced by top performing assemblers.pdf]

Supplementary Table S4. Basic assembly statistics for different assemblies of monocultures and mixed culture of *E. coli* and *K. pneumoniae* isolates. The mixed culture was obtained from co-culturing of *E. coli* 4 and *K. pneumoniae* 5 isolates. Average (SD)* values for MinION assembly were calculated after regarding the *E. coli* 3 and *K. pneumoniae* 1 isolate statistics as an outlier and discarding the corresponding data.

		Number of contigs	Largest contig (bp)	Total length (bp)	GC (%)	N50 (bp)
Illumina (Unicycler)	*E. coli* 1	100	765,416	5,052,925	50.57	254,457
	E. coli 2	68	761,444	5,077,031	50.58	281,982
	E. coli 3	115	620,652	5,066,581	50.79	261,702
	E. coli 4	269	323,518	5,735,389	50.42	102,833
	**Average (SD)**	**138 (90)**	**617,758 (207,392)**	**5,232,982 (335,084)**	**51 (1)**	**225,244 (82,435)**
MinION (Flye)	*E. coli* 1	50	570,493	5,073,148	50.49	222,053
	E. coli 2	113	128,944	4,225,573	50.37	47,034
	E. coli 3	5	17,872	49,176	39.56	16,045
	E. coli 4	27	2,970,614	6,134,097	50.77	1,087,804
	**Average (SD)**	**49 (47)**	**921,981 (1,386,454)**	**3,870,499 (2,664,510)**	**48 (6)**	**343,234 (504,598)**
	**Average * (SD) ***	**64 (45)**	**1,223,351 (1,529,196)**	**5,144,273 (956,248)**	**51 (1)**	**452,297 (557,279)**
Hybrid (Unicycler)	*E. coli* 1	30	1,929,634	5,121,243	50.61	1,392,053
	E. coli 2	25	1,352,860	5,102,867	50.59	1,296,669
	E. coli 3	82	636,775	5,088,863	50.79	336,032
	E. coli 4	62	275,7897	5,956,168	50.51	996,338
	**Average (SD)**	**50 (28)**	**1,669,292 (897,972)**	**5,317,286 (426,129)**	**51 (1)**	**1,005,273 (476,961)**
Illumina (Unicycler)	*K. pneumoniae* 1	82	653,447	5,374,307	57.35	251,480
	K. pneumoniae 2	57	815,437	5,465,685	57.10	348,149
	K. pneumoniae 3	84	479,668	5,797,728	56.67	200,979
	K. pneumoniae 4	88	862,848	5,737,695	56.82	350,054
	K. pneumoniae 5	77	927,949	5,510,849	57.64	311,143
	**Average (SD)**	**78 (13)**	**747,870 (181,013)**	**5,577,253 (181,931)**	**58 (1)**	**247,095 (138,114)**
MinION (Flye)	*K. pneumoniae* 1	30	53,004	715,682	55.53	33,058
	K. pneumoniae 2	3	5,315,630	5,519,519	57.09	5,315,630
	K. pneumoniae 3	9	3,308,867	5,906,847	56.61	3,308,867
	K. pneumoniae 4	54	2,675,543	5,907,851	56.73	1,219,455
	K. pneumoniae 5	78	326,735	5,424,990	57.67	103,491
	**Average (SD)**	**35 (32)**	**2,335,956 (2,190,273)**	**4,694,978 (2,235,357)**	**57 (1)**	**1,996,101 (2,279,327)**
	**Average * (SD) ***	**36 (37)**	**2,906,694 (2,055,427)**	**5,689,802 (254,150)**	**58 (1)**	**2,486,861 (2,306,831)**
Hybrid (Unicycler)	*K. pneumoniae* 1	43	866,749	5,411,988	57.33	499,601
	K. pneumoniae 2	4	5,321,058	5,530,587	57.06	5,321,058
	K. pneumoniae 3	8	5,319,674	5,907,459	56.60	5,319,674
	K. pneumoniae 4	14	5,308,446	5,832,296	56.75	5,308,446
	K. pneumoniae 5	28	2,952,457	5,558,222	57.59	2,952,457
	**Average**	**20 (17)**	**3,953,677 (2,006,405)**	**5,648,111 (211,443)**	**58 (1)**	**3,880,248 (2,149,256)**
Illumina (Unicycler)	Mix culture	371	927,949	11,193,506	53.97	147,235
MinION (Flye)	Mix culture	120	1,141,119	11,827,293	53.99	344,695
Hybrid (Unicycler)	Mix culture	117	5,202,502	11,495,693	53.93	1,245,846
