# Supplementary material for: Hybrid Assembly Provides Improved Resolution of Plasmids, Antimicrobial Resistance Genes, and Virulence Factors in Escherichia coli and Klebsiella pneumoniae Clinical Isolates
Source: Microorganisms. 2021 Dec 10;9(12):2560. doi: 10.3390/microorganisms9122560 (PMC8704702; doi:10.3390/microorganisms9122560)
Supplement: Supplementary file 1 [file microorganisms-09-02560-s001.zip › Supplementary Table S5_Summary of prokka annotation results for top performing assemblers.pdf]

Supplementary Table S5. An overview of values for annotation of genomic features of different assemblies from monocultures and mixed culture of *E. coli* and *K. pneumoniae* isolates. Average (SD)* were calculated after regarding the *E. coli* 3 and *K. pneumoniae* 1 isolate statistics as an outlier and discarding the corresponding data.

	Isolates ID	CDS	rRNA	tRNA	tmRNA	Putative proteins	Hypothetical proteins	Repeat regions
Illumina (Unicycler)	*E. coli 1*	4739	4	83	1	13	1149	0
	E. coli 2	4751	6	79	1	12	1149	2
	E. coli 3	4778	4	78	1	11	1191	0
	E. coli 4	5538	5	89	1	10	1789	2
	**Average (SD)**	**4952 (392)**	**5 (1)**	**83 (5)**	**1 (0)**	**12 (2)**	**1320 (314)**	**1 (2)**
MinION (Flye)	*E. coli 1*	8594	13	88	1	13	2814	0
	E. coli 2	7551	11	67	1	8	2701	2
	E. coli 3	67	0	0	0	0	47	0
	E. coli 4	10648	22	95	1	15	4451	2
	**Average (SD)**	**6715 (4615)**	**12 (10)**	**63 (44)**	**1 (1)**	**9 (7)**	**2504 (1823)**	**1 (2)**
	**Average * (SD) ***	**8931 (1576)**	**16 (6)**	**84 (15)**	**1 (0)**	**12 (4)**	**3322 (980)**	**2 (2)**
Hybrid (Unicycler)	*E. coli 1*	4794	22	95	1	13	1177	0
	E. coli 2	4722	11	80	1	12	1155	2
	E. coli 3	4813	4	78	1	11	1202	0
	E. coli 4	5837	22	98	1	11	1948	2
	**Average (SD)**	**5042 (532)**	**15 (9)**	**88 (11)**	**1 (0)**	**12 (1)**	**1371 (386)**	**1 (2)**
Illumina (Unicycler)	*K. pneumoniae 1*	4992	3	78	1	17	1283	2
	K. pneumoniae 2	5092	3	78	1	16	1250	0
	K. pneumoniae 3	5379	4	79	1	17	1528	2
	K. pneumoniae 4	5411	4	78	1	17	1608	0
	K. pneumoniae 5	5130	5	79	1	15	1275	0
	**Average (SD)**	**5201 (185)**	**4 (1)**	**79 (1)**	**1 (0)**	**17 (1)**	**1389 (167)**	**1 (2)**
MinION (Flye)	*K. pneumoniae 1*	1466	4	3	0	0	928	0
	K. pneumoniae 2	7082	25	86	1	17	2227	0
	K. pneumoniae 3	8576	25	88	1	17	3230	2
	K. pneumoniae 4	11847	25	82	1	17	5696	1
	K. pneumoniae 5	11625	19	73	1	7	6107	0
	**Average (SD)**	**8120 (3933)**	**20 (10)**	**67 (36)**	**1 (1)**	**12 (8)**	**3638 (2227)**	**1 (1)**
	**Average * (SD) ***	**7826 (2339)**	**19 (3)**	**66 (7)**	**1 (0)**	**12 (5)**	**3452 (1885)**	**1 (1)**
Hybrid (Unicycler)	*K. pneumoniae 1*	5026	9	79	1	17	1293	2
	K. pneumoniae 2	5130	25	86	1	16	1258	0
	K. pneumoniae 3	5487	25	87	1	17	1553	2
	K. pneumoniae 4	5494	25	85	1	17	1625	0
	K. pneumoniae 5	5166	17	84	1	15	1285	0
	**Average (SD)**	**5261 (217)**	**21 (8)**	**85 (4)**	**1 (0)**	**17 (1)**	**1403 (173)**	**1 (2)**
Illumina (Unicycler)	Mixed culture	10660	8	164	2	26	3114	2
MinION (Flye)	Mixed culture	20158	47	181	2	30	8174	1
Hybrid (Unicycler)	Mixed culture	10995	44	184	2	27	3261	2
